# Outcomes after right ventricular outflow tract reconstruction with valve substitutes: A systematic review and meta-analysis

**DOI:** 10.3389/fcvm.2022.897946

**Published:** 2022-09-07

**Authors:** Xu Wang, Wouter Bakhuis, Kevin M. Veen, Ad J. J. C. Bogers, Jonathan R. G. Etnel, Carlijn C. E. M. van Der Ven, Jolien W. Roos-Hesselink, Eleni-Rosalina Andrinopoulou, Johanna J. M. Takkenberg

**Affiliations:** ^1^Department of Cardiothoracic Surgery, Erasmus Medical Center, University Medical Center Rotterdam, Rotterdam, Netherlands; ^2^Department of Cardiology, Erasmus Medical Center, University Medical Center Rotterdam, Rotterdam, Netherlands; ^3^Department of Biostatistics, Erasmus Medical Center, University Medical Center Rotterdam, Rotterdam, Netherlands; ^4^Department of Epidemiology, Erasmus Medical Center, University Medical Center Rotterdam, Rotterdam, Netherlands

**Keywords:** right ventricular outflow tract reconstruction, Tetralogy of Fallot, truncus arteriosus, ross procedure, xenograft, homograft

## Abstract

**Introduction:**

This study aims to provide an overview of outcomes after right ventricular outflow tract (RVOT) reconstruction using different valve substitutes in different age groups for different indications.

**Methods:**

The literature was systematically searched for articles published between January 2000 and June 2021 reporting on clinical and/or echocardiographic outcomes after RVOT reconstruction with valve substitutes. A random-effects meta-analysis was conducted for outcomes, and time-related outcomes were visualized by pooled Kaplan–Meier curves. Subgroup analyses were performed according to etiology, implanted valve substitute and patient age.

**Results:**

Two hundred and seventeen articles were included, comprising 37,078 patients (age: 22.86 ± 11.29 years; 31.6% female) and 240,581 patient-years of follow-up. Aortic valve disease (Ross procedure, 46.6%) and Tetralogy of Fallot (TOF, 27.0%) were the two main underlying etiologies. Homograft and xenograft accounted for 83.7 and 32.6% of the overall valve substitutes, respectively. The early mortality, late mortality, reintervention and endocarditis rates were 3.36% (2.91–3.88), 0.72%/y (95% CI: 0.62–0.82), 2.62%/y (95% CI: 2.28–3.00), and 0.38%/y (95%CI: 0.31–0.47) for all patients. The early mortality for TOF and truncus arteriosus (TA) were 1.95% (1.31–2.90) and 10.67% (7.79–14.61). Pooled late mortality and reintervention rate were 0.59%/y (0.39–0.89), 1.41%/y (0.87–2.27), and 1.20%/y (0.74–1.94), 10.15%/y (7.42–13.90) for TOF and TA, respectively. Endocarditis rate was 0.21%/y (95% CI: 0.16–0.27) for a homograft substitute and 0.80%/y (95%CI: 0.60–1.09) for a xenograft substitute. Reintervention rate for infants, children and adults was 8.80%/y (95% CI: 6.49–11.95), 4.75%/y (95% CI: 3.67–6.14), and 0.72%/y (95% CI: 0.36–1.42), respectively.

**Conclusion:**

This study shows RVOT reconstruction with valve substitutes can be performed with acceptable mortality and morbidity rates for most patients. Reinterventions after RVOT reconstruction with valve substitutes are inevitable for most patients in their life-time, emphasizing the necessity of life-long follow-up and multidisciplinary care. Follow-up protocols should be tailored to individual patients because patients with different etiologies, ages, and implanted valve substitutes have different rates of mortality and morbidity.

**Systematic review registration:**

[www.crd.york.ac.uk/prospero], identifier [CRD42021271622].

## Background

Right ventricular outflow tract (RVOT) reconstruction is one of the most common surgical procedures in patients with congenital heart disease (CHD) (1), and a valve substitute is often used in this reconstruction to restore the connection between the pulmonary artery and right ventricle. Many underlying etiologies, e.g., Tetralogy of Fallot (TOF), truncus arteriosus (TA), and aortic valve disease (AVD: Ross procedure), are treated with RVOT reconstruction with valve substitutes (2–4). Patients with different etiologies have different RVOT anatomies, and this could impact patient prognosis. From this standpoint, exploring outcomes after RVOT reconstruction should be done by taking different etiologies into account. Additionally, several types of valve substitutes are available nowadays, whereas the optimal valve substitute is still subject to debate despite many studies have been performed on this topic (5–7). Furthermore, patient age should be considered when exploring the outcomes after RVOT reconstruction with valve substitutes (8, 9).

So far, there have been many publications concerning outcomes after RVOT reconstruction with valve substitutes. Age and types of valve substitutes have been explored. Some factors, for example, young age and small conduit size, are considered as risk factors for early conduit degenerations. However, most of the published studies on this topic are small and single-center, and provide fragmented information, only concerning a specific group of patients (10–12). Compared with one single original research, systematic review and meta-analysis can provide higher level of evidence. Therefore, a systematic review and meta-analysis was performed to provide a contemporary overview of outcomes after RVOT reconstruction with different valve substitutes, in different etiologies and different age groups.

## Methods

The protocol of this study was reviewed and approved by the Medical Ethics Review Committee of the Erasmus University Medical Center (MEC 2015–170). To establish an overview of reported outcomes, we conducted a systematic review and meta-analysis according to the Preferred Reporting Items for Systematic Reviews and Meta-Analyses (PRISMA) guidelines (13). Only surgical RVOT reconstruction with valve substitutes were included for analyses. This study was registered in the PROSPERO registry (CRD42021271622).

### Literature search strategy

The “population, intervention, comparison, outcome and study design” (PICOS) strategy was used to define our research question. The detailed description of PICOS strategy are presented in Supplementary Text 1.

The systematic literature search was conducted on June 28, 2021, in PubMed, MEDLINE, Embase, Web of Science, Cochrane, and Google Scholar by a biomedical information specialist (W.M.B). Search terms are available in Supplementary Text 2.

Original studies written in English reporting outcomes after surgical pulmonary valve replacement in human subjects were included. Studies had to report on more than 20 patients and be published after January 1, 2000. Focus of this study was mid-to long-term outcomes and only studies with follow-up longer than 1 year were included. Since homografts, xenografts and mechanical valves (MV) are the main types of grafts used in RVOT reconstruction with valve substitutes, we only included studies using these three valve substitutes. Exclusion criteria were non-original research, percutaneous procedures, follow-up complete <90%, no relevant outcome information, lacking essential information (length of follow-up, institutions), valve substitutes other than homograft, xenograft, and MV (i.e., polytetrafluoroethylene valve, autologous pericardial conduits).

Two reviewers (XW and WB) independently screened all publications from the systematic literature search. In the case of multiple publications on overlapping study populations, the publication with the longest total follow-up in patient-years or best overall completeness of data was included for each outcome of interest separately. In case of disagreement, an agreement was negotiated with a third independent reviewer (J.J.M.T.).

### Data extraction

Microsoft Office Excel 2016 (Microsoft Corp., Redmond, WA, United States) was used for data extraction. Data were extracted independently by 2 reviewers (X. W. and W. B.). Recorded study characteristics, baseline patient and operative characteristics, and outcome events are listed in the Supplementary Table 1.

Morbidity and mortality were documented according to the Akins guidelines for reporting mortality and morbidity after cardiac valve interventions (14). Early mortality was defined as either operative, within 30 days post-surgery or within initial hospital stay. Late mortality was any death beyond this period. Only very few studies reported events concerning structural valve deterioration (SVD) or non-structural valve dysfunction (NSVD) directly. Many articles only provided the information on conduit/valve dysfunction, without specifying it as SVD or NSVD, according to echocardiographic parameters. So, we decided to use valve dysfunction to describe valve substitute status. The definition of valve dysfunction varies among studies as well, and we unified it by selecting the definition that most studies adopted after reviewing all included studies. Conduit/valve dysfunction was defined according to echocardiographic parameters with one of the following descriptions: a. peak pulmonary valve (PV) gradient >36 or 40 mmHg; b. peak Doppler velocity >3 m/s; c. moderate or severe stenosis/regurgitation. We only extracted the information on dysfunction in line with the above definition. If total follow-up duration in patient-years was not reported, it was calculated by multiplying the number of patients with the mean follow-up duration of that study.

### Statistical analysis

Pooled baseline patient characteristics were calculated with the use of sample size weighting. If variable means and/or standard deviations were not reported, we used medians and range or interquartile range to calculate them by the method proposed by Luo et al. (15), Wan et al. (16), and Walter et al. (17). Early mortality and linearized occurrence rates of late morbidity and mortality were calculated for each study and pooled with the use of inverse variance weighting on a logarithmic scale because Shapiro-Wilk test revealed a skewed distribution among the majority of outcomes. When the number of studies was sufficiently large to reliably estimate the tau-squared statistic (≥4 studies), which is the variance between studies, a random-effects model was used to estimate pooled effects. When estimating pooled effects from less than 4 studies, a fixed-effects model was used. In case a particular event was reported not to occur in an individual study, it was assumed that 0.5 patients experienced that event for pooling purposes (continuity correction). Subgroup analyses were conducted according to the age (infants, children, and adults), indications (Ross procedure, right-sided conduit), and implanted valve substitutes (homograft, xenograft). We defined “right-sided conduit” as RVOT reconstruction with valve substitutes for abnormal RVOT anatomy, e.g., TOF and TA. Within the right-sided conduit subgroup, studies concerning TOF and TA were pooled separately to give a more specific overview of different diagnostic groups. Heterogeneity between studies was assessed with the Cochran Q and I^2^-statistic. Potential causes of heterogeneity in early/late mortality and rates of reintervention, reoperation, dysfunction, and endocarditis were explored utilizing univariable random-effects meta-regression. Sensitivity analyses were performed by temporarily excluding the smallest quartile (by sample size) or leave-one-out sensitivity analysis. Twenty percent was selected as the cut-off value of “major change” in the sensitivity analysis. Statistical analyses were performed in Microsoft Office Excel 2016 and the R statistical software (Version 3.3.3, R development Core Team, R Foundation for Statistical Computing, Vienna, Austria) using the metaphor package. A *P*-value < 0.05 was considered significant.

The pooling of Kaplan-Meier curves was done for survival probability, freedom from reintervention, and freedom from endocarditis by using the method described by Guyot (18). Published Kaplan–Meier curves were digitized and an estimate of the individual patient time-to-event data was then extrapolated from the digitized curve coordinates, assuming a constant rate of censorship between each time point at which the number of patients at risk was specified (19). We used Engauge Digitizer 9.7 to create a list of coordinates of the KM curve and employed an in-house developed algorithm written in R language (Version 4.1.2, R Development Core Team; R Foundation for Statistical Computing) to reconstruct the original patient data.

## Results

**FIGURE 1:**
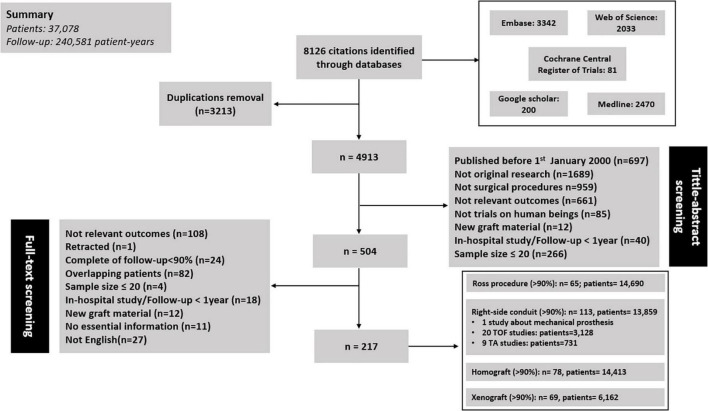
A flowchart of included studies.

**FIGURE 2:**
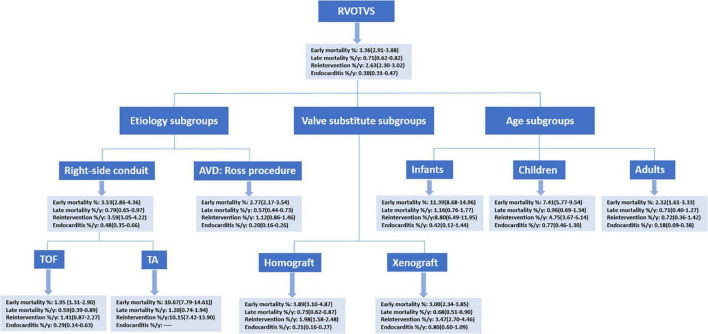
A summary of pooled estimates. RVOTVS, right ventricular outflow tract reconstruction with valve substitutes.

The literature search resulted in 8126 publications, of which 217 studies were included for analysis: 65 concerning RVOT reconstruction in Ross procedure, 113 concerning right-sided conduit (20 about TOF; 9 about TA), 78 concerning RVOT reconstruction with a homograft, and 69 concerning RVOT reconstruction with a xenograft (Figure 1). These 217 articles included 37,078 patients encompassing 240,581 patient-years of follow-up. All of the included studies were cohort studies. One study concerning MV was identified (20), and excluded from pooling analyses given the marked differences in characteristics between MV and biological valve substitutes. The summary of pooled outcomes in the overall group and different subgroups is shown in Figure 2.

### Overall group analyses

Baseline characteristics of the overall group and the subgroups are shown in Table 1. Pooled mean follow-up time was 6.49 ± 4.92 years. The most common etiology was AVD (Ross procedure, 46.6%), and TOF accounted for 27.0% of all included patients. Individual study characteristics are presented in Supplementary Table 2.

**TABLE 1:** Pooled baseline characteristics.

Characteristics		Pooled estimates (range or mean ± SD)				
		Overall *N* = 217	Subgroups			
			Ross (*N* = 65)	Right-sided conduit (*n* = 112)	Homograft (*N* = 78)	Xenograft (*N* = 69)
Age (years)		22.86 ± 11.29	36.26 ± 11.76	13.79 ± 9.26	26.56 ± 12.03	14.26 ± 9.76
Female (%)		31.6 (8.3–62.1)	26.9 (8.3–45.3)	44.3 (26.1–62.1)	31.5 (8.3–58.3)	42.4 (26.1–60.0)
Implantation year		2002 (1965–2020)	2002 (1986–2020)	2002 (1981–2019)	2001 (1965–2019)	2007 (1986–2019)
Preoperative NYHA III-IV (%)		32.5 (6.0–100.0)	34.0 (6.0–76.7)	26.7 (6.3–100.0)	34.2 (12.9–76.7)	24.5 (6.3–50.0)
Patients with previous cardiac procedures (%)		49.8 (0.0–100.0)	24.9 (0.0–100.0)	76.8 (0.0–100.0)	34.7 (0.0–100.0)	80.4 (0.0–100.0)
Patients with concomitant procedures (%)*		51.2 (2.0–100.0)	50.1 (1.8–100.0)	65.6 (8.0–100.0)	69.9 (16.7–100.0)	64.0 (8.0–100.0)
Conduit diameter (mm)		22.28 ± 3.33	25.00 ± 2.51	20.99 ± 2.98	22.19 ± 3.45	22.57 ± 2.91
Follow-up (years)		6.49 ± 4.92	7.79 ± 5.99	5.85 ± 4.01	7.02 ± 5.73	4.50 ± 2.70
Causes [n (%)]	AVD	17261 (46.6%)	14690 (100.0)	188 (3.3)	10679 (74.1)	384 (6.2)
	TOF	9989 (27.0%)	−	7434 (55.1)	1247 (8.7)	3251 (52.8)
	TA	2813 (7.6%)	−	2086 (15.5)	677 (4.7)	475 (7.7)
	PA	2197 (5.9%)	−	1488 (11.0)	470 (3.3)	709 (11.5)
	TGA	1217 (3.3%)	−	806 (6.0)	315 (2.2)	287 (4.7)
	PS/PR	760 (2.1%)	−	451 (3.3)	40 (0.3)	266 (4.3)
	DORV	734 (2.0%)	−	408 (3.0)	50 (0.4)	207 (3.4)
	Others**	2092 (5.5%)	−	578 (2.8)	935 (6.3)	583 (9.4)
RVOT grafts	homograft (%)	61.1	83.7	36.5	−	−
	Xenograft (%)***	32.6	12.6	62.1	−	−
	Mechanical valves (%)	1.2	0.0	0.5	−	−

N, number of studies reporting on the variable; SD, standard deviation; NYHA, New York Heart Association; Ross, Ross procedure; TOF, Tetralogy of Fallot; TA, truncus arteriosus; PA, pulmonary atresia; TGA, transposition of the great arteries; PS/PR, pulmonary stenosis/regurgitation; DORV, Double outlet right ventricle; AVD, aortic valve disease.

*Aortic valve procedures were excluded for Ross procedure; **others include absent pulmonary valve, endocarditis, rheumatic disease, redo pulmonary valve replacement (unknown original diagnosis), unknown causes, etc. We also regarded the three studies without information on diagnoses as unknown causes; ***xenograft was defined as valves or valved conduits originating from non-human species.

Pooled outcomes were reported in two parts: early outcomes and late outcomes. Outcomes of the overall group are given in Table 2. Early mortality, perioperative bleeding, stroke, and MI were 3.36% (2.91–3.88), 5.70% (4.79–6.78), 1.22% (0.93–1.60), and 1.25% (0.81–1.92), respectively. Late mortality was 0.72%/y (95% CI: 0.62–0.82). The rates of late reintervention, endocarditis, PMI/ICD, stroke, and TE/VT were 2.62, 0.38, 0.43, 0.18, and 0.29%/y, respectively.

**TABLE 2:** Overall pooled outcomes.

Outcomes		Pooled estimate (95% CI)	Heterogeneity (I^2^)	*N* studies reported
**Early outcomes (%)**				
Early mortality	All cause	3.36 (2.91–3.88)	74.8%	190
	Cardiac	2.30 (1.92–2.76)	59.9%	152
	Valve-related	0.74 (0.60–0.91)	0.0%	144
	SUD	0.69 (0.56–0.86)	0.0%	142
Early PMI/ICD		2.53 (2.00–3.20)	51.9%	57
Re-exploration for bleeding		5.70 (4.79–6.78)	60.2%	53
Early stroke		1.22 (0.93–1.60)	0.0%	32
Early TE/VT		1.00 (0.57–1.74)	45.3%	27
Early MI		1.25 (0.81–1.92)	42.0%	26
AKI		3.28 (2.25–4.78)	49.2%	22
**Late outcomes (%/y)**				
Late mortality	All cause	0.71 (0.62–0.82)	79.1%	189
	Cardiac	0.43 (0.37–0.50)	38.5%	150
	Valve-related	0.25 (0.21–0.29)	0.0%	136
	SUD	0.20 (0.17–0.23)	0.0%	141
Late PMI/ICD		0.43 (0.24–0.78)	73.1%	22
Late stroke		0.18 (0.12–0.26)	34.0%	23
Late TE/VT		0.29 (0.21–0.42)	62.8%	45
Late MI		0.07 (0.04–0.14)	11.3%	11
**Overall (%/y)**				
Reintervention		2.63 (2.30–3.02)	95.8%	209
Reoperation		1.69 (1.45–1.97)	93.7%	178
Dysfunction		3.05 (2.25–4.14)	95.1%	45
Endocarditis		0.38 (0.31–0.47)	75.9%	111
Moderate to severe PS		1.89 (1.42–2.52)	90.8%	59
Moderate to severe PR		2.02 (1.60–2.54)	94.2%	99

Data expressed as percentage (95% CI). CI, confidential interval; SUD, sudden unexplained death; PMI, permanent pacemaker implantation; ICD, implantable cardioverter defibrillator; TE, thromboembolism event; VT, valve thrombosis; MI, myocardial infarction; AKI, acute kidney injury; PS, pulmonary valve stenosis; PR, pulmonary valve regurgitation.

**FIGURE 3:**
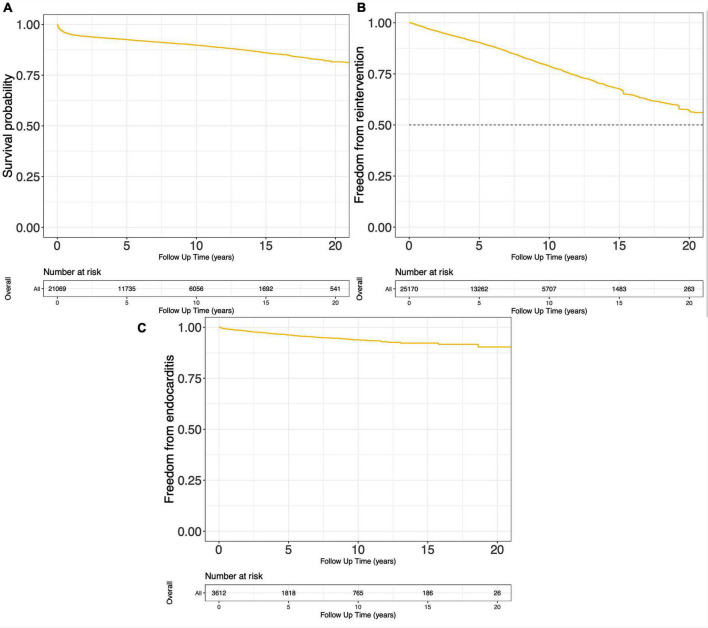
Pooled Kaplan-Meier curves of freedom death **(A)**, freedom from reintervention **(B)**, and freedom from endocarditis **(C)**.

Ninety-four studies reported the Kaplan-Meier curves on survival probability, encompassing 21,069 surgical cases in total. These curves were pooled, and the reconstructed Kaplan-Meier curve of the 94 studies is presented in Figure 3A. Survival probability at 5, 10, and 15 years was 92.6, 89.7, and 86.0%, respectively. Reconstructions of Kaplan-Meier curves on freedom from reintervention and endocarditis are displayed in Figures 3B,C. Freedom from reintervention and endocarditis at 5, 10, and 15 years were 90.4, 78.9, 67.6, and 96.2, 93.8, 91.9%, respectively (Supplementary Table 3).

### Subgroup analyses

#### Ross procedure

Sixty-five publications encompassing 14,690 patients reported relevant outcomes after Ross procedure. The pooled mean age in Ross procedure subgroup was 36.26 ± 11.76 years old, and 83.7% cases in this subgroup used homografts for RVOT reconstruction. The pooled baseline characteristics are presented in Table 1.

**FIGURE 4:**
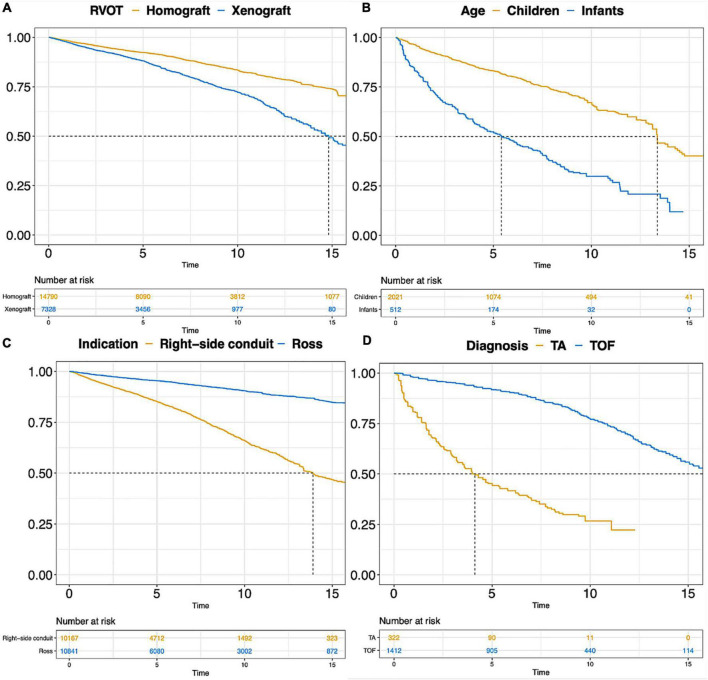
Pooled Kaplan-Meier curves of freedom reintervention in different subgroups. **(A)** Freedom from reintervention in subgroups of RVOT with homograft and RVOT with xenograft; **(B)** freedom from reintervention in subgroups of children and infants; **(C)** freedom from reintervention in subgroups of “Ross” and “Right-sided conduit”; **(D)** freedom from reintervention in subgroups of patients diagnosed with TA and TOF. TA, truncus arteriosus; TOF, Tetralogy of Fallot.

The pooled early mortality and late mortality in Ross procedure subgroup were 2.77% (2.17, 3.54) and 0.57% (0.44, 0.73). Reintervention rate (RVOT) and endocarditis rate (RVOT) were 1.12%/y (0.86, 1.46) and 0.20%/y (0.16, 0.26) after Ross procedure. More outcome information is shown in Table 3A. The pooled Kaplan-Meier curves of freedom from reintervention and death are shown in Figure 4C (blue line) and Supplementary Figure 1C (blue line).

**TABLE 3:** Pooled outcomes of different subgroups.

A							

		Indications					
		Ross procedure (≥90%, *N* = 65)			Right-sided conduit (≥90%, *N* = 112)		
Outcomes		Estimate (95% CI)	*N*	*I* ^2^	Estimate (95% CI)	*N*	*I* ^2^
**Early outcomes (%)**							
Early mortality (%)	All cause	2.77 (2.17, 3.54)	63	73.9%	3.53 (2.86, 4.36)	96	71.9%
	Cardiac	2.10 (1.66, 2.66)	50	29.7%	2.46 (1.89, 3.20)	80	61.6%
	Valve-related	0.79 (0.55, 1.13)	43	0.0%	0.79 (0.59, 1.06)	75	0.0%
	SUD	0.67 (0.46, 0.99)	41	0.0%	0.79 (0.59, 1.06)	76	0.0%
Early PMI/ICD		1.98 (1.48, 2.65)	29	39.9%	3.43 (2.33, 5.05)	24	57.0%
Re-exploration for bleeding		5.33 (4.24, 6.71)	28	64.8%	5.88 (4.30, 8.05)	21	57.9%
Early stroke		1.11 (0.80, 1.53)	20	0.0%	1.56 (0.93, 2.59)	12	0.0%
Early TE/VT		0.79 (0.41, 1.51)	13	0.0%	1.43 (0.58, 3.52)	12	61.5%
Early MI		1.03 (0.59, 1.79)	16	37.5%	1.37 (0.74, 2.53)	7	0.0%
AKI		2.64 (1.74, 4.01)	14	40.4%	7.13 (3.52, 14.44)	6	35.1%
**Late outcomes (%/y)**							
Late mortality	All cause	0.57 (0.44, 0.73)	59	83.5%	0.79 (0.65, 0.97)	96	75.1%
	Cardiac	0.34 (0.28, 0.41)	53	29.1%	0.55 (0.44, 0.68)	77	33.9%
	Valve-related	0.20 (0.16, 0.26)	48	0.0%	0.30 (0.24, 0.38)	74	0.0%
	SUD	0.17 (0.13, 0.23)	45	0.0%	0.23 (0.18, 0.30)	76	0.0%
Late PMI/ICD		0.31 (0.18, 0.52)	12	35.2%	1.02 (0.46, 2.27)	8	61.5%
Late stroke		0.17 (0.11, 0.26)	17	41.3%	0.17 (0.06, 0.49)	4	0.7%
Late TE/VT		0.21 (0.15, 0.29)	23	19.8%	0.57 (0.29, 1.15)	14	72.1%
Late MI		0.07 (0.03, 0.15)	6	0.0%	0.22 (0.05, 0.96)	3	42.1%
**Overall (%/y)**							
Reintervention		1.12 (0.86, 1.46)	64	94.0%	3.59 (3.05, 4.22)	107	94.3%
Reoperation		0.70 (0.51, 0.97)	53	92.3%	2.27 (1.91, 2.70)	95	90.9%
Endocarditis		0.20 (0.16, 0.26)	40	36.7%	0.48 (0.35, 0.66)	47	59.9%
Dysfunction		1.47 (0.85, 2.55)	13	92.7%	4.67 (3.43, 6.36)	23	88.1%
Moderate to severe PS		1.11 (0.68, 1.83)	21	89.2%	3.23 (2.43, 4.30)	30	79.6%
Moderate to severe PR		0.97 (0.60, 1.57)	28	92.4%	2.87 (2.29,3.59)	53	84.0%

**B**							

		**Indications**					
		**TOF (*N* = 20)**			**TA (*N* = 9)**		
**Outcomes**		**Estimate (95% CI)**	***N***	***I* ^2^**	**Estimate (95% CI)**	***N***	***I* ^2^**

**Early outcomes (%)**							
Early mortality	All cause	1.95 (1.31, 2.90)	19	38.1%	10.67 (7.79, 14.61)	9	39.3%
	Cardiac	1.47 (0.86, 2.52)	16	26.0%	8.59 (5.77, 12.80)	6	21.0%
	Valve-related	All zero event	14	−	1.40 (0.63, 3.10)	6	0.0%
	SUD	All zero event	14	−	0.99 (0.40, 2.49)	6	0.0%
Early PMI/ICD		2.14 (1.02, 4.49)	8	67.7%	−	1	−
Re-exploration for bleeding		6.23 (4.20, 9.26)	2	0.0%	13.09 (6.02, 28.49)	3	62.1%
Early stroke		−	1	−	−	1	−
Early TE/VT		−	1	−	−	0	−
Early MI		−	1	−	3.32 (0.85, 12.95%)	2	31.7%
AKI		−	1	−	−	1	−
**Late outcomes (%/y)**							
Late mortality	All cause	0.59 (0.39, 0.89)	18	63.3%	1.20 (0.74, 1.94)	9	66.8%
	Cardiac	0.53 (0.40, 0.70)	16	2.7%	0.36 (0.23, 0.56)	6	0.0%
	Valve-related	0.25 (0.15, 0.42)	12	0.0%	0.21 (0.11, 0.38)	5	0.0%
	SUD	0.25 (0.15, 0.42)	13	0.0%	0.21 (0.11, 0.39)	5	0.0%
Late PMI/ICD		1.69 (0.46, 6.24)	3	73.7%	−	0	−
Late stroke		−	0	−	−	1	−
Late TE/VT		−	1	−	−	0	−
Late MI		−	0	−	−	0	−
**Overall (%/y)**							
Reintervention		1.41 (0.87, 2.27)	17	92.7%	10.15 (7.42, 13.90)	9	91.4%
Reoperation		1.02 (0.74, 1.41)	15	58.6%	5.02 (3.83, 6.58)	6	73.3%
Endocarditis		0.29 (0.14, 0.63)	3	4.4%	−	0	−
Dysfunction		3.07 (1.60, 5.88)	5	87.9%	−	1	−
Moderate to severe PS		1.34 (0.64, 2.83)	4	33.1%	−	1	−
Moderate to severe PR		1.22 (0.52, 2.89)	7	68.7%	−	1	−

**C**							

		**Valve substitute**					
		**Homograft (≥90% of cases, *N* = 78)**			**Xenograft (≥90%, *N* = 69)**		
**Outcomes**		**Estimate (95% CI)**	***N***	***I* ^2^**	**Estimate (95% CI)**	***N***	***I* ^2^**

Early outcomes (%)							
Early mortality	All cause	3.89 (3.10, 4.87)	72	80.2%	3.00 (2.34, 3.85)	65	50.0%
	Cardiac	2.52 (1.85, 3.43)	52	64.6%	2.45 (1.84, 3.26)	58	37.7%
	Valve-related	0.69 (0.49, 0.97)	49	0.0%	0.82 (0.58, 1.17)	56	0.0%
	**SUD**	0.66 (0.44, 0.98)	46	0.0%	0.72 (0.50, 1.04)	57	0.0%
Early PMI/ICD		2.17 (1.46, 3.24)	21	45.5%	3.92 (2.95, 5.21)	19	0.0%
Re-exploration for bleeding		5.53 (4.17, 7.33)	22	63.7%	5.66 (4.20, 7.62)	16	24.4%
Early stroke		1.20 (0.74, 1.94)	13	0.0%	1.38 (0.71, 2.69)	10	0.0%
Early TE/VT		0.82 (0.38, 1.76)	9	0.0%	1.62 (0.58, 4.52)	10	60.8%
Early MI		1.74 (1.02, 2.98)	13	13.7%	2.51 (1.19, 5.29)	5	31.7%
AKI		2.87 (1.71, 4.80)	10	45.6%	4.89 (2.24, 10.68)	7	52.5%
**Late outcomes (%/y)**							
Late mortality	All cause	0.73 (0.62, 0.87)	71	64.6%	0.68 (0.51, 0.90)	63	53.9%
	Cardiac	0.39 (0.31, 0.48)	55	36.2%	0.54 (0.39, 0.74)	52	20.0%
	Valve-related	0.21 (0.17, 0.27)	55	0.0%	0.29 (0.21, 0.41)	51	0.0%
	SUD	0.16 (0.12, 0.21)	53	0.0%	0.28 (0.19, 0.40)	51	0.0%
Late PMI/ICD		0.33 (0.18, 0.59)	11	38.8%	0.94 (0.35, 2.56)	6	49.1%
Late stroke		0.26 (0.19, 0.36)	11	9.1%	0.32 (0.08, 1.21)	4	36.7%			
Late TE/VT		0.16 (0.10, 0.26)	15	17.8%	0.56 (0.24, 1.31)	14	64.7%			
Late MI		0.05 (0.02, 0.11)	7	0.0%	−	1	−			
**Overall (%/y)**										
Reintervention		1.98 (1.58, 2.48)	77	94.6%	3.47 (2.70, 4.46)	66	92.4%			
Reoperation		1.33 (1.00, 1.76)	61	92.8%	2.20 (1.71, 2.84)	63	86.3%			
Endocarditis		0.21 (0.16, 0.27)	36	30.3%	0.80 (0.60,1.09)	39	56.3%			
Dysfunction		3.06 (2.05, 4.57)	19	94.7%	2.46 (1.30, 4.68)	13	91.1%			
Moderate to severe PS		1.46 (0.95, 2.24)	22	89.3%	1.97 (1.17, 3.32)	20	82.0%			
Moderate to severe PR		1.63 (1.08, 2.47)	31	93.9%	2.67 (2.02, 3.52)	44	82.6%			

**D**										

		**Neonates/infants (*N* = 11)**			Children (*N* = 34)*			**Adult (*N* = 18)**		
**Outcomes**		**Estimate (95% CI)**	***N***	***I* ^2^**	**Estimate (95% CI)**	***N***	***I* ^2^**	**Estimate (95% CI)**	***N***	***I* ^2^**

**Early outcomes (%)**										
Early mortality	All cause	11.39 (8.68, 14.96)	11	37.56%	7.41 (5.77, 9.54)	31	64.55%	2.32 (1.61, 3.33)	16	53.24%
	Cardiac	8.37 (6.03, 11.63)	6	0.00%	5.53 (3.99, 7.66)	25	54.18%	1.65 (1.17, 2.33)	12	0.00%
	Valve-related	1.20 (0.48, 3.01)**	6	0.00%	1.36 (0.85, 2.16)	24	0.00%	0.59 (0.27, 1.32)**	11	0.00%
	SUD	1.20 (0.48, 3.01)**	6	0.00%	0.88 (0.51, 1.52)	23	0.00%	0.59 (0.27, 1.32)**	11	0.00%
Early PMI/ICD		3.13 (1.20, 8.21)	2	0.00%	3.53 (1.43, 8.70)	7	60.08%	1.40 (0.85, 2.29)	6	23.84%
Re-exploration for bleeding		13.08 (7.79, 21.98)	4	46.32%	4.48 (2.21, 9.05)	7	44.41%	6.61 (4.49, 9.73)	9	75.53%
Early stroke		1.32 (0.33, 5.25)	2	0.00%	1.25 (0.32, 4.85)	4	30.40%	1.14 (0.77, 1.69)	10	0.00%
Early TE/VT		0 event	2	−	−	1	−	1.54 (0.39, 6.07)	3	0.00%
Early MI		−	1	−	2.08 (0.88, 4.94)	5	0.00%	1.55 (0.36, 6.76)	3	66.43%
AKI		4.31 (1.66, 11.20)	2	0.00%	4.89 (1.47, 16.32)	2	33.37%	2.40 (0.58, 9.99)	3	66.16%
**Late outcomes (%/y)**										
Late mortality	All cause	1.16 (0.76, 1.77)	11	52.43%	0.96 (0.69, 1.34)	30	69.96%	0.71 (0.40, 1.27)	16	91.11%
	Cardiac	0.94 (0.40, 2.22)	5	6.14%	0.58 (0.37, 0.91)	21	48.29%	0.36 (0.28, 0.47)	16	23.93%
	Valve-related	0.38 (0.09, 1.52)**	3	0.00%	0.24 (0.16, 0.35)	24	0.00%	0.26 (0.17, 0.40)	13	0.00%
	SUD	0.38 (0.09, 1.52)**	3	0.00%	0.21 (0.13, 0.31)	24	0.00%	0.23 (0.15, 0.34)	14	0.00%
Late PMI/ICD		−	0	−	0.38 (0.23, 0.64)	6	0.00%	−	1	−
Late stroke		0.23 (0.06, 0.90)	2	0.00%	−	1	−	0.20 (0.12, 0.34)	6	45.47%
Late TE/VT		0.53 (0.20, 1.42)	2	0.00%	0.71 (0.34, 1.49)	5	6.72%	0.25 (0.16, 0.38)	6	0.00%
Late MI		−	0	−	−	1	−	−	0	−
**Overall (%/y)**										
Reintervention		8.80 (6.49, 11.95)	11	90.00%	4.75 (3.67, 6.14)	34	93.02%	0.72 (0.36, 1.42)	16	93.39%
Reoperation		4.48 (3.10, 6.48)	6	83.88%	2.98 (2.24, 3.95)	28	88.24%	0.65 (0.28, 1.50)	13	93.53%
Endocarditis		0.42 (0.12, 1.44)	3	9.55%	0.77 (0.46, 1.30)	17	66.51%	0.18 (0.09, 0.38)	12	71.96%
Dysfunction		−	0	−	4.97 (2.37, 10.45)	5	92.19%	0.97 (0.48, 1.97)	7	78.52%
Moderate to severe PS		−	1	−	5.55 (3.61, 8.55)	7	75.75%	0.45 (0.22, 0.92)	9	71.84%
Moderate to severe PR		−	1	−	3.64 (2.10, 6.31)	14	91.94%	0.62 (0.35, 1.08)	12	68.88%

Data expressed as percentage (95% CI). CI, confidential interval; SUD, sudden unexplained death; PMI, permanent pacemaker implantation; ICD, implantable cardioverter defibrillator; TE, thromboembolism event; VT, valve thrombosis; MI, myocardial infarction; AKI, acute kidney injury; PS, pulmonary valve stenosis; PR, pulmonary valve regurgitation. TOF, Tetralogy of Fallot; TA, truncus arteriosus; “−”, no attempt to pool outcomes. *Some studies contain a part of infants and the results about infants and non-infant-children are inseparable. These studies were attributed to “Children” group. **The two outcomes have exactly same publications reporting them and both have same number.

#### Right-sided conduit

In total, 113 publications encompassing 13,859 patients reported outcomes after right-sided conduit implantation. One of them was about mechanical prostheses and was excluded from the analyses. Finally, 112 studies comprising 13,495 patients with 79,143 patient-years were included for analyses in this subgroup.

The pooled mean age was 14.19 ± 9.35 years old, and the main valve substitutes implanted in RVOT were xenograft, which had a 62.1% proportion. Two main etiologies were TOF (55.8%) and TA (15.2%) in this subgroup. More details of the baseline characteristics are shown in Table 1.

The pooled early mortality was 3.53% (2.86, 4.36) after right-sided conduit implantation. Late mortality, reintervention rate and endocarditis rate were 0.79% (0.65, 0.97), 2.77% (2.17, 3.54), and 0.57% (0.44, 0.73). More outcome information is shown in Table 3A. The pooled Kaplan-Meier curves of freedom from reintervention and death are shown in Figure 4C (yellow line) and Supplementary Figure 1C (yellow line).

##### Tetralogy of Fallot

Twenty publications about TOF were pooled separately, encompassing 3,128 patients with 19,466 patient-years. The average age at RVOT reconstruction with valve substitutes was 24.95 ± 11.62 years. The pooled baseline characteristics of the TOF subgroup are presented in Supplementary Table 4. The pooled outcomes of the TOF subgroup are shown in Table 3.

##### Truncus arteriosus

Nine studies concerning TA were pooled. In total, 731 patients with 7,613 patient-years were encompassed in this subgroup. The pooled baseline characteristics are presented in Supplementary Table 4. The pooled age was 0.19 ± 0.92 years, and 79.0% of TA patients received the homograft as the valve substitute. The pooled outcomes of the TA subgroup are presented in Table 3. Early mortality was 10.67% and the rate of PV reintervention was 10.15%/y.

The Kaplan-Meier curves of freedom from death and reintervention of the two subgroups, TA and TOF, were pooled and are displayed in Figure 4D and Supplementary Figure 1D. More than half of repaired TA patients require RVOT reintervention within 5 years after the initial repair surgery. A rapid decline of survival probability in the first 1–2 years after RVOT reconstruction with valve substitutes was observed in the TA subgroup, which stabilized thereafter.

#### Homograft and xenograft

Sixty-nine studies encompassing 6,162 patients with 27,408 patient-years were pooled in xenograft subgroup analysis and 78 studies compromising 14,413 patients with 99,679 patients were pooled in homograft subgroup. Pooled baseline characteristics of the homograft and xenograft subgroups are shown in Table 2. The patient age at RVOT reconstruction with homograft and with xenograft was 26.56 ± 12.03 and 14.26 ± 9.76 years. Aortic valve disease was the main etiology for patients with homograft implantation while TOF was the dominant cause for xenograft implantation in RVOT.

**FIGURE 5:**
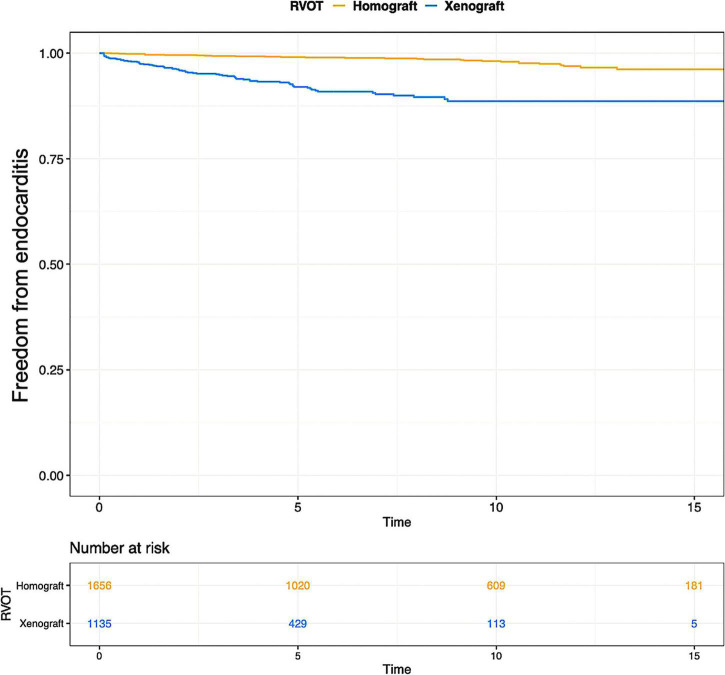
Pooled Kaplan-Meier curves of freedom from endocarditis of two subgroups: RVOT reconstruction with xenograft and homograft. The blue line stands for xenograft and the yellow line stands for homograft.

Various types of xenograft had been used, and the Contegra conduit accounted for 41.9% of the xenograft and was the mainly implanted conduit. The proportions of other commonly used types of xenograft were 18.9% for the Carpentier-Edwards aortic pericardial valve (including Perimount, Magna, and Magna Ease) and 11.4% for the Medtronic Freestyle. Pooled outcome estimates in the two groups are presented in Table 3. Rates of endocarditis in the homograft group and xenograft group were 0.21%/y (95% CI: 0.16–0.27%/y) and 0.80%/y (95% CI: 0.60–1.09%/y). Pooled Kaplan-Meier curves of freedom from reintervention and endocarditis are shown separately for the two subgroups in Figures 4A, 5.

The pooled results of studies concerning only the Contegra conduit are shown in Supplementary Tables 5, 6. Patients who received Contegra conduit were younger compared with the overall xenograft group (Contegra group: 7.41 ± 8.20 year-old; overall xenograft group: 14.26 ± 9.76 year-old). The endocarditis and reintervention rates were 1.17%/y (95% CI: 0.86, 1.59) and 5.74% (95% CI: 4.38, 7.52) for Contegra group, which were higher than the overall xenograft group (endocarditis: 0.80%/y, 95% CI: 0.60–1.09; reintervention: 3.47%/y, 95%CI: 2.70–4.46).

##### Ross procedure: Homograft and xenograft

Patients older than 16 years old in Ross procedure subgroup were pooled separately based on the type of implanted valve substitutes. Their respective pooled baseline characteristics are presented in Supplementary Table 7. Fourteen publications encompassing 5,040 patients reported the outcomes after Ross procedure with homografts and 5 studies compromising 1,618 patients reported outcomes of Ross procedure with xenografts. The outcomes of the two subgroups are displayed in Supplementary Table 8.

##### Right-sided conduit: Homograft and xenograft

Within the right-sided conduit group, there were 19 articles containing 1,960 surgical cases that concerned homograft implantations and 53 publications comprising 4,790 surgical cases with xenograft implantations.

Baseline characteristics of the two subgroups are displayed in Supplementary Table 9. TOF was the main etiology in both subgroups. TA accounted for 26.9% of cases in the homograft subgroup (right-sided conduit), and the corresponding proportion is 7.0% in the xenograft subgroup (right-sided conduit). For right-sided conduit surgical cases, the pooled mean age of homograft implantation and xenograft implantation is 9.35 ± 7.40 and 15.11 ± 9.62 years.

**FIGURE 6:**
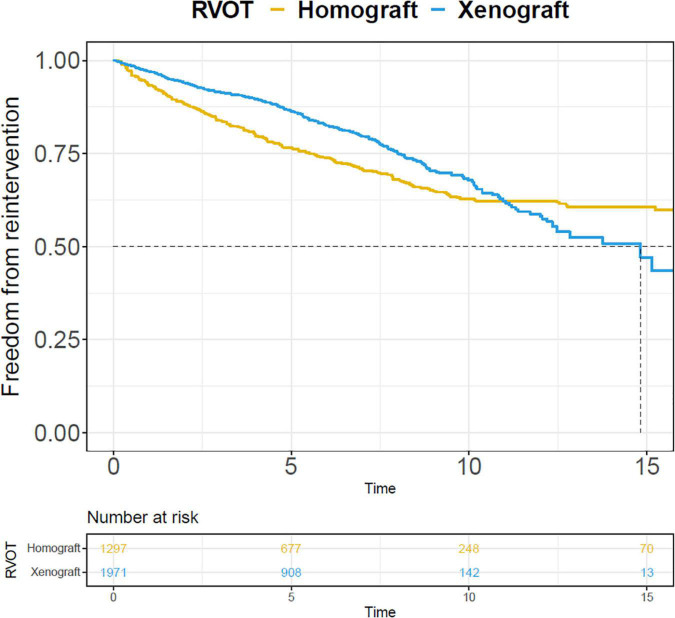
Pooled Kaplan-Meier curves of freedom from reintervention of two subgroups: right-sided conduit RVOT with xenograft and homograft. The blue line stands for xenograft and the yellow line stands for homograft.

Their respective pooled outcomes are presented in Supplementary Table 10. The rates of reintervention and endocarditis were 4.66, 0.24, and 3.23, 0.69%/y in the right-sided homograft conduit and right-sided xenograft conduit subgroup, respectively. The Kaplan-Meier curves of freedom from reintervention were pooled in the two subgroups (Figure 6).

#### Mechanical valve prostheses

Only one multi-center study with 364 patients and 1,705 patient-years was remained after full-text screening in mechanical valve subgroup. Pooling was not possible. In that MV study, its reoperation rate was 0.98%/y, lower than the rate in homograft (1.33%/y) and xenograft (2.20%/y) RVOT subgroup. The late mortality and thromboembolism events rates were 1.17 and 1.91%/y in the MV multicenter study.

#### Infants (≤1 y), children (<18 y), and adults (≥18 y)

The pooled baseline characteristics of the three age subgroups are shown in Supplementary Table 11. The main etiology was TA in infants (70.6%). As to adults who require RVOT reconstruction with valve substitutes, the main indication was aortic valve disease (89.3%).

Pooled outcomes of the three age subgroups are presented in Table 3D. The early mortality was 11.39% in infants, 7.41% in children, and 2.32% in adults. The rates of reintervention and reoperation were 8.80%/y (95% CI: 6.49–11.95%/y) and 4.48%/y (95% CI: 3.10–6.48%/y) in infants. The rates of reintervention and reoperation were 0.72%/y (95% CI: 0.36–1.42%/y) and 0.65%/y (95% CI: 0.28–1.50%/y) in adults. Pooled Kaplan-Meier curves of freedom from reintervention and death in infants and children subgroups are shown in Figure 4B and Supplementary Figure 1B.

## Heterogeneity and publication bias

### Meta-regression

Univariable meta-regression was done for early mortality, late mortality, reintervention, reoperation, dysfunction, and endocarditis, to identify sources of heterogeneity within overall group. Studies with younger age (−0.063, *p* < 0.001), higher female proportion (4.853, *p* < 0.001), smaller conduit diameter (−0.170, *p* < 0.001), right-sided conduit procedures (Ross: 1.177, *p* < 0.001), and xenograft implantation (0.562, *p* = 0.001) were inclined to report higher rate of reintervention. More details of meta-regression are displayed in Supplementary Table 12.

### Sensitivity analyses

The results of sensitivity analyses are presented in Supplementary Table 13. Major changes in pooled outcomes of early valve-related mortality, early sudden unexplained death (SUD), early and late permanent pacemaker implantation/Implantable cardioverter-defibrillator (PMI/ICD), early thromboembolism/valve thrombosis (TE/VT), and early MI were found, suggesting publication bias. Leave-one-out analyses were done on these “major-change” outcomes and the results are shown in Supplementary Table 14. Based on the “major-change” definition, which is higher than 20%, no major change was found in leave-one-out sensitivity analyses.

## Discussion

This study provides a comprehensive overview of contemporary early and late outcomes after RVOT reconstruction with valve substitutes for different etiologies, with different valve substitutes and at different patient ages. Patients operated for various etiologies have different outcomes, including mortality, reintervention, reoperation, and endocarditis rates. Within each etiology subgroup, age and valve substitutes have their roles in influencing patients’ outcomes.

According to the pooled baseline characteristics of the different subgroups in this study, there are strikingly different distributions of patients’ age and implanted valve substitutes, which are in accordance with the clinical indications for RVOT reconstruction. Most TA patients undergo RVOT reconstruction in their infancy while the majority of Ross procedures take place in young adulthood. More homografts than xenografts were implanted in younger patients based on the pooled baseline characteristics. Given these observations, it is challenging to discuss etiology, patients’ age, and valve substitutes separately. Therefore, the following discussion part focuses on different etiological settings and explores the role of patients’ age and valve substitutes within each etiological subgroup.

### Different etiologies

Various etiologies may require RVOT reconstruction with valve substitutes. The implantation positions of valve substitutes could be divided into two categories based on the RVOT anatomical structure: anatomical position and extra-anatomical position. The former one refers to normal RVOT structure, which commonly occurs in patients undergoing the Ross procedure; the later one refers to abnormal RVOT structure, which presents in patients requiring RVOT reconstruction for complex congenital heart disease, for example, TOF, TA (21). Some studies have tried to study whether the survival of conduits is different in the two positions (21–24). Despite existing disagreement, the accumulated evidence supports that anatomical position implantation is associated with longer valve substitutes survival compared to extra-anatomic implantation (21, 23, 24). Our pooled rates of reintervention, reoperation, and dysfunction are in favor of the better conduit survival in anatomical position. This may be because greater hemodynamic stress is imposed on the implanted conduits in patients with CHD, possibly due to high pulmonary vascular resistance and abnormal pulmonary vascular anatomy (25), despite the compression of the conduit in the extra-anatomical position against the sternum possibly being another reason for accelerated degradation.

Ross procedures (anatomical position) are more commonly performed in developed than developing countries. The quality of health care is different in the two types of country. To get more insights into the possible reasons for superior outcomes of Ross procedure patients, geographical distributions of included articles about Ross procedures and right-sided conduit RVOT (extra-anatomical position) were calculated (Supplementary Table 15). More Ross procedures were performed in Europe countries and more right-sided conduit implantations were done in Asia. This geographical differences could be another explanation for the superior outcomes of RVOT valve substitute in anatomical position compared with the one in extra-anatomical position.

#### Right-sided conduit: Tetralogy of Fallot

Tetralogy of Fallot is the most common form of cyanotic CHD (26). The improvements in surgical repair of TOF enable more patients to reach adulthood (27). The corrective surgery, however, is not curative and decades after repair, adults are often faced with the consequences of chronic pulmonary regurgitation (PR) that requires implanting valve substitutes in RVOT (28). Despite the necessity of redo-RVOT reconstruction as the sequelae of primary repair, the risk of it is quite low, with early and late patient mortality being 1.95 and 0.59%/y. On top of that, the reintervention rate after the redo-RVOT reconstruction is relatively low, and most patients can enjoy long-term reintervention-free survival. There is no consensus on which type of valve substitute is preferred over the other. Prior research suggests that homografts and xenografts have comparable performance (7, 29), but there are indications of a higher rate of endocarditis in xenografts compared with homografts (30, 31).

Within the TOF subgroup, no more specific analyses were done between homografts and xenografts to explore the differences in performance because of the limited number of relevant publications. However, there are major differences in the rates of mortality and reintervention between homografts and xenografts subgroups accounting for all etiologies (Table 3). The Kaplan-Meier curves of freedom from endocarditis are divergent between homografts and xenografts, as shown in Figure 5. It gives us insight into the possible higher hazard of endocarditis in xenografts compared with homografts. Various possible hypotheses have been proposed to explain the increased incidence of endocarditis among xenografts, e.g., bacterial adhesion, infiltration, collagenization, and inflammation (32). Besides, some types of xenografts are only available in small sizes, e.g., Contegra. Small-sized conduits could have more turbulent flow, which could lead to higher endocarditis risk. However, the pooled size of xenografts is larger than homografts in Supplementary Table 9, but the pooled endocarditis risk is higher in xenograft than homograft. In Table 1, patients receiving xenografts were more frequently diagnosed with right-sided CHDs which have more complex RVOT abnormalities. These abnormal anatomies could impose more mechanical shear stress or compression on implanted valve grafts, thus resulting in early deterioration and a higher endocarditis rate. Intravenous drug abuse is widely accepted as a prominent risk factor for right-sided infected endocarditis (IE) (33). Commonly, the tricuspid valve is more likely to be infected compared with the pulmonary valve in this situation (33). However, the majority of the reported IE in our included studies concerns the pulmonary valve with or without the aortic valve, not the tricuspid valve. Moreover, patients’ conditions of drug use have not been clarified in most of the included studies. It is therefore difficult to assess whether the high rate of IE in xenografts is associated with intravenous drug use or not.

Most TOF patients who underwent RVOT reconstruction with conduits after total correction are young adults. It indicates their lower probabilities of a hyperactive immune response, fibrosis, and somatic growth causing consequent patient-conduit size mismatch in contrast with infants and young children (34). In our subgroup analyses regarding patient age, adults have the longest valve substitutes durability as opposed to infants and children. Therefore, the relatively low rates of RVOT reintervention and dysfunction in the TOF subgroup could be partially due to its age demographics.

#### Right-sided conduit: Truncus arteriosus

Truncus arteriosus is a rare CHD, with a considerable early perioperative mortality, ranging from 3 to 20%, and a high rate of reintervention after initial repair (2). Hitherto, most studies concerning TA have a small sample size and relatively short follow-up. Furthermore, there is no published systematic review and meta-analysis regarding outcomes after TA repair. For this reason, our TA subgroup pooling results provide valuable insights into the prognosis of this group of patients.

Different from TOF patients, most TA patients have their right-sided conduit implanted in their neonatal period. The effect of an immune response resulting in stenosis is still being debated, but patient-conduit size mismatch would definitively occur as a consequence of somatic growth (34). All of these aspects could result in a high rate of conduit reintervention after TA repair, which is more than 10%/y in this study. More than half of TA patients may expect at least one RVOT reintervention within 5 years after the initial TA repair operation. The hazard is higher in the first 4–5 years than in the phase afterward. It suggests that strict follow-up should be given to infants after TA repair, especially in the first 4–5 years. Of note, many TA patients died peri-operatively and early post-operatively, especially in the first 1–2 years after TA repair. This may result in the underestimation of the true reintervention rate since some patients died before they needed a reintervention. Most TA patients received homograft conduits between the right ventricle and pulmonary artery in our study. Both homografts and xenografts are prone to structural deterioration, especially in infants and young children (35, 36). There have been no perfect valve substitutes so far. Recently, many heart valve substitutes that are manufactured with new technologies are becoming available, for example, tissue-engineered heart valves and decellularized heart valves. Some studies have proven the satisfactory duration of these new valve substitutes (37, 38). It could be possible in the future that xenografts produced with new technologies have a performance comparable with homografts when it come s to durability and endocarditis.

Young patients are growing, which adds difficulties to the long survival of implanted conduits. More studies are required in the future to explore the optimal valve substitute for young babies with TA since the number of studies focusing on investigating this issue is quite small.

#### Aortic valve disease: Ross procedure

Patients undergoing the Ross procedure have normal anatomy on the RVOT. This allows for a conduit in anatomical position to sustain less hemodynamic stress, thus having longer survival than the conduit of extra-anatomic implantation (21, 23, 24). This is also clearly reflected by our results, with a significantly lower RVOT reintervention rate in the Ross procedure subgroup compared with the right-sided conduit subgroup.

Patients’ age plays an important role in affecting the outcomes of the Ross procedure, with younger age being associated with a higher rate of valve degeneration (39, 40). Our group has studied the associations between age and outcomes after the Ross procedure by performing age-subgroup analyses (39, 40). According to their findings, adults have the lowest RVOT reintervention rate and infants have the highest. Age-related differences in calcium metabolism, immune activity, somatic growth, and hemodynamics are hypothesized to play a role in the mechanism behind the phenomenon (39).

Homografts are the first choice in RVOT reconstruction for the Ross procedure. Despite many new valve substitutes being available, homograft utilization is still very high according to our findings (83.7%). Due to the limited availability of homografts, potential comparable alternatives have been proposed, for example, xenograft bioprostheses. Some studies have found xenografts to be inferior to homografts regarding RVOT conduit deterioration after the Ross procedure (41, 42). In this systematic review and meta-analysis, no analysis was attempted in this regard owing to the limited number of relevant studies and sample size. To provide more convincing evidence, research with a large sample size concerning the comparison of different RVOT valve substitutes in the Ross procedure is necessary for the future. Moreover, new approaches have been employed in manufacturing valve substitutes nowadays, for example, decellularization. They could prolong the survival of traditional conduits (43). Future studies should also take these new technologies into account.

### Future perspectives

Some innovative methods are available to provide more patient-oriented information, and the microsimulation model is one of them (44). The microsimulation model translates the aggregated data to individual patients’ information. Both estimated outcomes from meta-analyses and primary datasets can be used to fit this model. Microsimulation in conjunction with datasets of large sample size or meta-analytical studies can provide robust long-term outcome estimates that allow for the age- and sex-specific insights into what patients can be expected to face during their lives after undergoing a certain intervention, for example, pulmonary valve replacement (44). Valuable information to patients and clinicians can be represented in a meaningful format by using microsimulation. The results of this study can be used to inform patients and clinicians of the information relating to the expected outcome after RVOT reconstruction with valve substitutes in different settings and serve as input for novel microsimulation models.

## Limitations

This is a systematic review and meta-analysis of mainly retrospective observational studies. Therefore, the inherent limitations of pooling such studies apply to this study (45). Secondly, publication bias may be present which can potentially lead to underestimation of the estimates. We did not assess publication bias using funnel plots, as funnel plots do not allow for meaningful interpretation in case of absolute risk outcomes because of substantial methodological limitations, which may in itself give rise to funnel plot asymmetry (46). Thirdly, heterogeneity was present in most outcomes which may lead to inaccurate results. Nevertheless, we conducted a thorough examination of heterogeneity by meta-regression. Linearized occurrence rates assume a constant hazard over time, while most of the distribution of events may be time-related in fact (47). Therefore, Kaplan-Meier curves were pooled, illustrating the distribution of time-to-event. Furthermore, some outcomes showed major changes after studies with a sample size lower than the 25th percentile were eliminated. It indicates the possibility of publication bias in these “major-change” outcomes. Since the number of publications that reported “major-change” outcomes is much smaller compared with the whole group (n = 217), excluding many studies could inappropriately magnify the sensitivity of detecting publication bias, which could explain why excluding the lowest quartile has a substantial impact on estimates. That is why leave-one-out sensitivity analysis was done as well, and no major change was found in this analysis, indicating it is not a single study driving the found major change. Besides, the definition of RVOT conduit dysfunction differs from study to study: some studies only reported dysfunction at the time of reoperation or death, while others also employed echocardiographic criteria. The definition adopted in this study is based on echocardiographic parameters because most information could be retained by handling it in this way. Only a small proportion (45 out of 217) of studies were utilized regarding conduit dysfunction. This processing step leads to the loss of information and may diminish the power of finally pooled estimates. However, ensuring the same criterion is essential to obtaining accurate estimates. All valve substitutes from animal tissues were named “xenografts.” However, different types of xenograft have different properties and it would be better to do subgroup analyses more specifically, e.g., Contegra vs. Medtronic Freestyle. Due to the limited publications, only studies about Contegra conduits were pooled independently from other xenograft studies. Suboptimal results of Contegra compared with other types of xenograft were noticed. It might be due to the younger recipients of Contegra conduits than other xenografts. Besides, more xenografts that have been manufactured with new technologies are available nowadays, for example, decellularized xenografts. In the future, more studies as to new xenograft conduits need to be initiated and included in meta-analysis to compare their respective performance in the future. Studies concerning trans-catheter pulmonary valve replacement (TPVR) were eliminated from analyses because the focus of this meta-analysis is surgical pulmonary valve replacement (SPVR). However, more and more TPVRs have been performed in the past decade. There could be a high proportion of patients with previously implanted trans-catheter pulmonary valves at the time of SPVR. This ever-changing situation could have an impact on the outcomes of SPVR in the near future. Our systematic review and meta-analysis may need renewal in order to provide a more real-world overview of SPVR outcomes. Since there are no studies regarding MV being included for analysis, the findings in this research are not applied to patients undergoing RVOT reconstruction with mechanical valves. Since only a small number of studies included in the systematic review have a mean follow-up beyond 10 years, our conclusions may limit to the first postoperative decade. Lastly, not all relevant papers may have been included in this systematic review, even though an extensive search strategy was pursued. However, it may be possible some relevant published articles are missed due to uncommon keyword assignments or filing in medical databases (48). The results of one missed article were compared with our pooling results, and similarities were found in mortality and homograft reintervention rates (48). Additionally, almost 15 thousand patients undergoing the Ross procedure have been included and analyzed in this meta-analysis. With such a large sample size, the results and conclusions of our study should be robust and reliable enough for guiding clinical decision-making.

## Conclusion

This systematic review with meta-analysis provides a comprehensive overview of outcomes after RVOT reconstruction in different etiologies, with different valve substitutes and in different patient age groups. Follow-up should be tailored to patients’ characteristics because patients with different etiologies, ages, and implanted valve substitutes have different mortality and morbidity rates. Reinterventions after RVOT reconstruction are inevitable for most patients in their lifetime, emphasizing the necessity of life-long follow-up and multidisciplinary care.

## Data availability statement

The original contributions presented in the study are included in the article/Supplementary material, further inquiries can be directed to the corresponding author/s.

## Author contributions

XW and WB were responsible for the coordination and acquisition of the data. XW and CV performed the statistical analysis. XW drafted and revised the manuscript. JT conceived and designed the study and assist in drafting the manuscript. E-RA and KV assisted us in performing the statistical analysis. AB, JE, JR-H, KV, and JT contributed to the preparation and critical review of the manuscript. All authors approved the final manuscript.

## Abbreviations

RVOT: right ventricular outflow tract
CHD: congenital heart disease
TOF: Tetralogy of Fallot
TA: truncus arteriosus
AVD: aortic valve disease
PRISMA: Systematic Reviews and Meta-Analyses
SVD: structural valve deterioration
NSVD: non-structural valve dysfunction
PV: pulmonary valve
MV: mechanical valves
CI: confidence interval
NYHA: New York Heart Association
SUD: sudden unexplained death
PMI: permanent pacemaker implantation
ICD: Implantable cardioverter-defibrillator
TE: thromboembolism
VT: valve thrombosis
PR: pulmonary regurgitation
IE: infected endocarditis.

## References

[1] Mitchell MB. Pulmonary valve replacement for congenital heart disease: What valve substitute should we be using? *J Thorac Cardiovasc Surg.* (2016) 152:1230–2. 10.1016/j.jtcvs.2016.07.031 27637425

[2] Naimo PS Konstantinov IE. Surgery for truncus arteriosus: Contemporary practice. *Ann Thorac Surg.* (2020) 111:1442–50. 10.1016/j.athoracsur.2020.06.036 32828754

[3] Lee C Choi ES Lee CH. Long-term outcomes of pulmonary valve replacement in patients with repaired tetralogy of Fallot. *Eur J Cardiothorac Surg.* (2020) 58:246–52. 10.1093/ejcts/ezaa030 32047919

[4] Ross DN. Replacement of aortic and mitral valves with a pulmonary autograft. *Lancet.* (1967) 2:956–8. 10.1016/S0140-6736(67)90794-5 4167516

[5] Christenson JT Sierra J Colina Manzano NE Jolou J Beghetti M Kalangos A. Homografts and xenografts for right ventricular outflow tract reconstruction: Long-term results. *Ann Thorac Surg.* (2010) 90:1287–93. 10.1016/j.athoracsur.2010.06.078 20868830

[6] Homann M Haehnel JC Mendler N Paek SU Holper K Meisner H Reconstruction of the RVOT with valved biological conduits: 25 Years experience with allografts and xenografts. *Eur J Cardio Thoracic Surg.* (2000) 17:624–30. 10.1016/S1010-7940(00)00414-0 10856850

[7] Falchetti A Demanet H Dessy H Melot C Pierrakos C Wauthy P. Contegra versus pulmonary homograft for right ventricular outflow tract reconstruction in newborns. *Cardiol Young.* (2019) 29:505–10. 10.1017/S1047951119000143 30942148

[8] Breymann T Blanz U Wojtalik MA Daenen W Hetzer R Sarris G European Contegra multicentre study: 7-year results after 165 valved bovine jugular vein graft implantations. *Thorac Cardiovasc Surg.* (2009) 57:257–69. 10.1055/s-0029-1185513 19629887

[9] Brown JW Ruzmetov M Rodefeld MD Vijay P Turrentine MW. Right ventricular outflow tract reconstruction with an allograft conduit in non-ross patients: Risk factors for allograft dysfunction and failure. *Ann Thorac Surg.* (2005) 80:655–63; discussion 663–654. 10.1016/j.athoracsur.2005.02.053 16039222

[10] Vitanova K Cleuziou J Hörer J Kasnar-Samprec J Vogt M Schreiber C Which type of conduit to choose for right ventricular outflow tract reconstruction in patients below 1 year of age? *Eur J Cardio Thorac Surg.* (2014) 46:961–6. 10.1093/ejcts/ezu080 24616389

[11] Fiore AC Ruzmetov M Huynh D Hanley S Rodefeld MD Turrentine MW Comparison of bovine jugular vein with pulmonary homograft conduits in children less than 2 years of age. *Eur J Cardio Thorac Surg.* (2010) 38:318–25. 10.1016/j.ejcts.2010.01.063 20356755

[12] Sharifulin R Bogachev-Prokophiev A Demin I Afanasyev A Ovcharov M Pivkin A Allografts and xenografts for right ventricular outflow tract reconstruction in Ross patients. *Eur J Cardiothorac Surg.* (2020) 59:162–9. 10.1093/ejcts/ezaa244 32864698

[13] Moher D Shamseer L Clarke M Ghersi D Liberati A Petticrew M Preferred reporting items for systematic review and meta-analysis protocols (PRISMA-P) 2015 statement. *Syst Rev.* (2015) 4:1. 10.1186/2046-4053-4-1 25554246 PMC4320440

[14] Akins CW Miller DC Turina MI Kouchoukos NT Blackstone EH Grunkemeier GL Guidelines for reporting mortality and morbidity after cardiac valve interventions. *Ann Thorac Surg.* (2008) 85:1490–5. 10.1016/j.athoracsur.2007.12.082 18355567

[15] Luo D Wan X Liu J Tong T. Optimally estimating the sample mean from the sample size, median, mid-range, and/or mid-quartile range. *Stat Methods Med Res.* (2018) 27:1785–805. 10.1177/0962280216669183 27683581

[16] Wan X Wang W Liu J Tong T. Estimating the sample mean and standard deviation from the sample size, median, range and/or interquartile range. *BMC Med Res Methodol.* (2014) 14:135. 10.1186/1471-2288-14-135 25524443 PMC4383202

[17] Walter SD Yao X. Effect sizes can be calculated for studies reporting ranges for outcome variables in systematic reviews. *J Clin Epidemiol.* (2007) 60:849–52. 10.1016/j.jclinepi.2006.11.003 17606182

[18] Guyot P Ades AE Ouwens MJNM Welton NJ. Enhanced secondary analysis of survival data: Reconstructing the data from published Kaplan-Meier survival curves. *BMC Med Res Methodol.* (2012) 12:9. 10.1186/1471-2288-12-9 22297116 PMC3313891

[19] Borenstein M Hedges LV Higgins JP Rothstein HR. A basic introduction to fixed-effect and random-effects models for meta-analysis. *Res Synth Methods.* (2010) 1:97–111. 10.1002/jrsm.12 26061376

[20] Pragt H van Melle JP Javadikasgari H Seo DM Stulak JM Knez I Mechanical valves in the pulmonary position: An international retrospective analysis. *J Thorac Cardiovasc Surg.* (2017) 154:1371–8.e1371. 10.1016/j.jtcvs.2017.04.072 28697893

[21] Gerestein CG Takkenberg JJ Oei FB Cromme-Dijkhuis AH Spitaels SE van Herwerden LA Right ventricular outflow tract reconstruction with an allograft conduit. *Ann Thorac Surg.* (2001) 71:911–7; discussion 917–918. 10.1016/S0003-4975(00)02440-1 11269473

[22] Ruzmetov M Geiss DM Shah JJ Fortuna RS Welke KF. Does the homograft for RVOT reconstruction in ross: Patients fare better than for non-ross patients? A single-center experience. *J Heart Valve Dis.* (2015) 24:478–83. 26897820

[23] Selamet Tierney ES Gersony WM Altmann K Solowiejczyk DE Bevilacqua LM Khan C Pulmonary position cryopreserved homografts: Durability in pediatric Ross and non-Ross patients. *J Thorac Cardiovasc Surg.* (2005) 130:282–6. 10.1016/j.jtcvs.2005.04.003 16077388

[24] Forbess JM Shah AS St Louis JD Jaggers JJ Ungerleider RM. Cryopreserved homografts in the pulmonary position: Determinants of durability. *Ann Thorac Surg.* (2001) 71:54–9; discussion 59–60. 10.1016/S0003-4975(00)01788-4 11216810

[25] Yap CH Yii M. Factors influencing late allograft valve failure. *Scand Cardiovasc J.* (2004) 38:325–33. 10.1080/14017430410016387 15804797

[26] Bhagra CJ Hickey EJ Van De Bruaene A Roche SL Horlick EM Wald RM. Pulmonary valve procedures late after repair of tetralogy of fallot: Current perspectives and contemporary approaches to management. *Can J Cardiol.* (2017) 33:1138–49. 10.1016/j.cjca.2017.06.011 28843325

[27] Marelli AJ Ionescu-Ittu R Mackie AS Guo L Dendukuri N Kaouache M. Lifetime prevalence of congenital heart disease in the general population from 2000 to 2010. *Circulation.* (2014) 130:749–56. 10.1161/CIRCULATIONAHA.113.008396 24944314

[28] Woo JP McElhinney DB Lui GK. The challenges of an aging tetralogy of Fallot population. *Expert Rev Cardiovasc Ther.* (2021) 19:581–93. 10.1080/14779072.2021.1940960 34102942

[29] Marathe SP Bell D Betts K Sayed S Dunne B Ward C Homografts versus stentless bioprosthetic valves in the pulmonary position: A multicentre propensity-matched comparison in patients younger than 20 years. *Eur J Cardio-thorac Surg.* (2019) 56:377–84. 10.1093/ejcts/ezz021 30753373

[30] Sharma A Cote AT Hosking MCK Harris KC. A systematic review of infective endocarditis in patients with bovine jugular vein valves compared with other valve types. *JACC Cardiovasc Interv.* (2017) 10:1449–58. 10.1016/j.jcin.2017.04.025 28728659

[31] Mery CM Guzmán-Pruneda FA De León LE Zhang W Terwelp MD Bocchini CE Risk factors for development of endocarditis and reintervention in patients undergoing right ventricle to pulmonary artery valved conduit placement. *J Thorac Cardiovasc Surg.* (2016) 151:432–9, 441.e431–432. 10.1016/j.jtcvs.2015.10.069 26670191

[32] Beckerman Z De León LE Zea-Vera R Mery CM Fraser CD Jr. High incidence of late infective endocarditis in bovine jugular vein valved conduits. *J Thorac Cardiovasc Surg.* (2018) 156:728–34.e722. 10.1016/j.jtcvs.2018.03.156 29753513

[33] Syed IM Yanagawa B Jeyaganth S Verma S Cheema AN. Injection drug use endocarditis: An inner-city hospital experience. *CJC Open.* (2021) 3:896–903. 10.1016/j.cjco.2021.02.015 34401696 PMC8347875

[34] Holmes A Co S Human D LeBlanc J Campbell A. The Contegra conduit: Late outcomes in right ventricular outflow tract reconstruction. *Ann Pediatr Cardiol.* (2012) 5:27–33. 10.4103/0974-2069.93706 22529597 PMC3327010

[35] Luciani GB Santini F Mazzucco A. Autografts, homografts, and xenografts: Overview on stentless aortic valve surgery. *J Cardiovasc Med (Hagerstown).* (2007) 8:91–6. 10.2459/01.JCM.0000260208.98246.10 17299289

[36] Manji RA Lee W Cooper DKC. Xenograft bioprosthetic heart valves: Past, present and future. *Int J Surg.* (2015) 23(Pt B):280–4. 10.1016/j.ijsu.2015.07.009 26190838

[37] Durko AP Yacoub MH Kluin J. Tissue engineered materials in cardiovascular surgery: The surgeon’s perspective. *Front Cardiovasc Med.* (2020) 7:55. 10.3389/fcvm.2020.00055 32351975 PMC7174659

[38] Emmert MY Hoerstrup SP. Next generation heart valve substitutes. *Eur Heart J.* (2017) 38:617–8. 10.1093/eurheartj/ehx074 29121257

[39] Etnel JRG Grashuis P Huygens SA Pekbay B Papageorgiou G Helbing WA The ross procedure: A systematic review, meta-analysis, and microsimulation. *Circ Cardiovasc Qual Outcomes.* (2018) 11:e004748. 10.1161/CIRCOUTCOMES.118.004748 30562065

[40] Etnel JR Elmont LC Ertekin E Mokhles MM Heuvelman HJ Roos-Hesselink JW Outcome after aortic valve replacement in children: A systematic review and meta-analysis. *J Thorac Cardiovasc Surg.* (2016) 151:143–152.e141–143. 10.1016/j.jtcvs.2015.09.083 26541831

[41] Penov K Haugen MA Radakovic D Hamouda K Gorski A Leyh R Decellularized pulmonary xenograft matrix PplusN versus cryopreserved homograft for RVOT reconstruction during ross procedure in adults. *Thorac Cardiovasc Surg.* (2021): [Epub ahead of print]. 10.1055/s-0041-1740539 34972237

[42] Flynn CD De Bono JH Muston B Rattan N Tian DH Larobina M Systematic review and meta-analysis of long-term outcomes in adults undergoing the Ross procedure. *Ann Cardiothorac Surg.* (2021) 10:411–9. 10.21037/acs-2021-rp-30 34422553 PMC8339630

[43] Boethig D Horke A Hazekamp M Meyns B Rega F Van Puyvelde J A European study on decellularized homografts for pulmonary valve replacement: Initial results from the prospective ESPOIR Trial and ESPOIR Registry data†. *Eur J Cardiothorac Surg.* (2019) 56:503–9. 10.1093/ejcts/ezz054 30879050 PMC6735763

[44] Huygens SA Rutten-van Mölken MP Bekkers JA Bogers AJ Bouten CV Chamuleau SA Conceptual model for early health technology assessment of current and novel heart valve interventions. *Open Heart.* (2016) 3:e000500. 10.1136/openhrt-2016-000500 27843569 PMC5073474

[45] Ioannidis JP Lau J. Pooling research results: Benefits and limitations of meta-analysis. *Jt Comm J Qual Improv.* (1999) 25:462–9. 10.1016/S1070-3241(16)30460-6 10481815

[46] Sterne JA Egger M. Funnel plots for detecting bias in meta-analysis: Guidelines on choice of axis. *J Clin Epidemiol.* (2001) 54:1046–55. 10.1016/S0895-4356(01)00377-8 11576817

[47] Blackstone EH Naftel DC Turner ME. The decomposition of time-varying hazard into phases, each incorporating a separate stream of concomitant information. *J Am Stat Assoc.* (1986) 81:615–24. 10.1080/01621459.1986.10478314

[48] Luciani GB Lucchese G Carotti A Brancaccio G Abbruzzese P Caianiello G Two decades of experience with the Ross operation in neonates, infants and children from the Italian Paediatric Ross Registry. *Heart.* (2014) 100:1954–9. 10.1136/heartjnl-2014-305873 25056868

