# Supplementary material for: Outcomes after right ventricular outflow tract reconstruction with valve substitutes: A systematic review and meta-analysis
Source: Front Cardiovasc Med. 2022 Sep 7;9:897946. doi: 10.3389/fcvm.2022.897946 (PMC9489846; doi:10.3389/fcvm.2022.897946)
Supplement: Supplementary file 1 [file Data_Sheet_1.docx]

Supplementary Text S1: PICOS strategy descriptions of meta-analysis

P: Patients with heart valve diseases”.

I: Surgical RVOT reconstruction with valve substitutes .

C: No comparison was attempted.

O: Outcomes were defined by the Akins guidelines[1], including early and late outcomes. More information on extracted outcomes was mentioned in the “Data Extraction” part.

S: All types of studies were included as long as they contain relevant outcome information after surgical RVOT reconstruction with valve substitutes , regardless of their design.

Supplementary Text S2: Literature search query

**Embase.com**

('right ventricular outflow tract reconstruction'/de OR 'heart right ventricle outflow tract reconstruction'/de OR 'heart right ventricle outflow tract obstruction'/de/dm_su OR 'pulmonary valve replacement'/exp OR 'pulmonary valve prosthesis'/exp OR (('heart right ventricle outflow tract'/de OR 'heart right ventricle outflow tract obstruction'/de OR 'right ventricular outflow tract dysfunction'/de OR 'pulmonary valve'/de) AND ('surgery'/de OR reoperation/de OR 'surgical technique'/de OR 'postoperative period'/de OR 'heart surgery'/de OR 'thorax surgery'/de OR 'heart valve surgery'/exp OR 'heart valve replacement'/de OR 'heart valve bioprosthesis'/de)) OR ((right-ventric*-outflow-tract* OR rv-outflow-tract* OR rvot* OR pulmon*-valve* OR pulmon*-position*) NEAR/6 (reintervention* OR reconstruct* OR surger* OR prosthe* OR repair* OR implantation* OR reoperat* OR re-operat* OR postoperat* OR post-operat* OR conduit* OR replacement* OR mechanical-valve* OR prosthe*)):ab,ti,kw) AND ('congenital heart disease'/exp OR 'heart disease'/exp/dm_co OR 'pulmonary valve atresia'/exp OR 'pulmonary valve insufficiency'/de OR 'Fallot tetralogy'/de OR 'arterial trunk'/de OR 'Ross procedure'/de OR 'pulmonary valve stenosis'/exp OR (((congenital* OR malform*) NEAR/3 (heart OR cardiac)) OR ((pulmonary OR lung) NEAR/3 atresia*) OR fallot OR tof OR (arter* NEAR/3 trun*) OR (Ross NEAR/3 (procedure* OR surgery OR operat*)) OR ((pulmonar* OR lung) NEAR/3 valve* NEAR/6 (stenos* OR regurgitat* OR insuffic* OR dysfunction*)) OR double-chamber* OR (sept* NEAR/3 defect*)):ab,ti,kw) NOT ('case report'/de OR (case-report):ti) NOT ([animals]/lim NOT [humans]/lim) NOT ([Conference Abstract]/lim AND [1800-2019]/py) AND [English]/lim

**Medline Ovid**

(((Ventricular Outflow Obstruction/ OR Pulmonary Valve/) AND (Surgical Procedures, Operative/ OR Reoperation/ OR Postoperative Period/ OR Cardiac Surgical Procedures/ OR Thoracic Surgery/ OR Heart Valve Prosthesis Implantation/)) OR ((right-ventric*-outflow-tract* OR rv-outflow-tract* OR rvot* OR pulmon*-valve* OR pulmon*-position*) ADJ6 (reintervention* OR reconstruct* OR surger* OR prosthe* OR repair* OR implantation* OR reoperat* OR re-operat* OR postoperat* OR post-operat* OR conduit* OR replacement* OR mechanical-valve* OR prosthe*)).ab,ti,kf.) AND (exp Heart Diseases/co OR Pulmonary Atresia/ OR Pulmonary Valve Insufficiency/ OR Tetralogy of Fallot/ OR Ross procedure/ OR Pulmonary Valve Stenosis/ OR (((congenital* OR malform*) ADJ3 (heart OR cardiac)) OR ((pulmonary OR lung) ADJ3 atresia*) OR fallot OR tof OR (arter* ADJ3 trun*) OR (Ross ADJ3 (procedure* OR surgery OR operat*)) OR ((pulmonar* OR lung) ADJ3 valve* ADJ6 (stenos* OR regurgitat* OR insuffic* OR dysfunction*)) OR double-chamber* OR (sept* ADJ3 defect*)).ab,ti,kf.) NOT (case report/ OR (case-report).ti.) NOT (exp animals/ NOT humans/) AND english.la.

**Web of Science Core Collection**

TS=((((right-ventric*-outflow-tract* OR rv-outflow-tract* OR rvot* OR pulmon*-valve* OR pulmon*-position*) NEAR/5 (reintervention* OR reconstruct* OR surger* OR prosthe* OR repair* OR implantation* OR reoperat* OR re-operat* OR postoperat* OR post-operat* OR conduit* OR replacement* OR mechanical-valve* OR prosthe*))) AND ((((congenital* OR malform*) NEAR/2 (heart OR cardiac)) OR ((pulmonary OR lung) NEAR/2 atresia*) OR fallot OR tof OR (arter* NEAR/2 trun*) OR (Ross NEAR/2 (procedure* OR surgery OR operat*)) OR ((pulmonar* OR lung) NEAR/2 valve* NEAR/5 (stenos* OR regurgitat* OR insuffic* OR dysfunction*)) OR double-chamber* OR (sept* NEAR/2 defect*)))) NOT TI=(case-report) AND DT=(Article OR Review OR Letter OR Early Access) AND LA=(english)

**Cochrane Central Register of Trials**

(((right-ventric* NEXT outflow-tract* OR rv-outflow-tract* OR rvot* OR pulmon* NEXT valve* OR pulmon* NEXT position*) NEAR/6 (reintervention* OR reconstruct* OR surger* OR prosthe* OR repair* OR implantation* OR reoperat* OR re-operat* OR postoperat* OR post-operat* OR conduit* OR replacement* OR mechanical-valve* OR prosthe*)):ab,ti,kw) AND ((((congenital* OR malform*) NEAR/3 (heart OR cardiac)) OR ((pulmonary OR lung) NEAR/3 atresia*) OR fallot OR tof OR (arter* NEAR/3 trun*) OR (Ross NEAR/3 (procedure* OR surgery OR operat*)) OR ((pulmonar* OR lung) NEAR/3 valve* NEAR/6 (stenos* OR regurgitat* OR insuffic* OR dysfunction*)) OR double-chamber* OR (sept* NEAR/3 defect*)):ab,ti,kw)

**Google scholar**

"Right ventricular outflow tract reintervention|reconstruction|surgery|repair|implantation|conduit" "congenital heart|cardiac"|"heart|cardiac malformation"|"pulmonary|lung atresia"|fallot|"Ross procedure|surgery|operation"|"pulmonar|lung valve stenosis"

'Right ventricular outflow tract reintervention|reconstruction|surgery|repair|implantation|conduit' 'congenital heart|cardiac'|'heart|cardiac malformation'|'pulmonary|lung atresia'|fallot|'Ross procedure|surgery|operation'|'pulmonar|lung valve stenosis'

Supplementary Table 1: Extracted outcome and baseline variables

| Baseline variables | Outcomes | |
| --- | --- | --- |
| Implantation period | Early outcomes | |
| Participated institutions | -Early mortality | -Cardiac |
|  |  | -Valve-related |
|  |  | -SUD |
| Age | -Early pacemaker implantation/implantable cardioverter defibrillator | |
| Sex | -Early stroke | |
| NYHA class | -Early myocardial infarction | |
| PVR prosthesis | -Early thromboembolism/valve thrombosis | |
| -homograft | -Re-exploration for bleeding | |
| -xenograft | -Acute kidney injury | |
| -mechanical prosthesis | Late outcomes | |
| Study type | -Late mortality | -Cardiac |
|  |  | -Valve-related |
|  |  | -SUD |
| -retrospective | -Late pacemaker implantation/implantable cardioverter defibrillator | |
| -prospective | -Late stroke | |
| Sample size | -Late myocardial infarction | |
| Follow-up length | -Late thromboembolism/valve thrombosis | |
| Conduit diameter | -Late pacemaker implantation/implantable cardioverter defibrillator | |
| Original diagnoses | Overall | |
| Previous cardiac intervention | -Reintervention | |
| Concomitant procedures | -Reoperation | |
|  | -Dysfunction | |
|  | -Endocarditis | |
|  | -Moderate to severe pulmonary stenosis | |
|  | -Moderate to severe pulmonary regurgitation | |

^NYHA: New York heart association; PVR: pulmonary valve replacement; SUD: sudden unexplained death.^

Supplementary Table 2: Baseline characteristics of individual studies

| Author (year) | Inclusion period | Sample size (n) | Follow up (mean, year) | Age at PVR (year) | Female (%) | PVR grafts l | Prior cardiac procedures (%) | Main diagnosis |
| --- | --- | --- | --- | --- | --- | --- | --- | --- |
| Caldarone (2000) [2] | 1966-1996 | 726 | 8.51 | 6.93 | NA | mixed | NA | PA |
| V. Sirvydis(2000)[3] | 1993-1994 | 45 | 2.50 | 15.00 | 17.78% | homograft | NA | ROSS |
| Linden (2001)[4] | 1993-2000 | 40 | 2.83 | 33.30 | 30.00% | homograft | 7.50% | ROSS |
| Corno (2002)[5] | 1999-2001 | 26 | 1.23 | 13.50 | NA | xenograft | NA | ROSS |
| Bansi Koul(2002)[6] | 1996-2000 | 23 | 1.58 | 42.00 | 17.39% | homograft | NA | ROSS |
| Sakaguchi(2003)[7] | 1986-2000 | 399 | 4.50 | 24.19 | 24.06% | homograft | 45.11% | ROSS |
| Kenneth (2003)[8] | NA | 36 | 6.72 | 15.20 | 36.11% | mixed | 100.00% | TOF |
| Alphonso (2004)[9] | 1991-2002 | 60 | 5.78 | 17.93 | 36.67% | homograft | 40.00% | ROSS |
| Hraska (2004)[10] | 1997-2003 | 66 | 2.47 | 12.70 | NA | homograft | 56.06% | ROSS |
| Oosterhof (2006)[11] | 1986-2005 | 158 | 4.52 | NA | 40.51% | homograft | 100.00% | TOF |
| Jae(2010)[12] | 2000-2007 | 103 | 3.00 | 12.80 | NA | xenograft | 100.00% | TOF |
| Lindsey (2010)[13] | 1996-2008 | 42 | 2.17 | 8.00 | 35.71% | xenograft | 100.00% | TOF |
| Shinkawa (2010)[14] | 2002-2009 | 73 | 2.82 | 19.50 | 39.73% | xenograft | 94.52% | TOF |
| Jain(2012)[15] | 1996-2010 | 153 | 8.50 | 34.09 | 52.94% | mixed | 100.00% | TOF |
| Jang (2012)[16] | 2001-2010 | 131 | 4.20 | 14.80 | 32.06% | xenograft | 100.00% | TOF |
| Vohra(2012)[17] | 1999-2010 | 37 | 3.91 | 25.84 | NA | xenograft | 94.59% | TOF |
| Babu-Narayan (2014)[18] | 1993-2010 | 220 | 3.14 | 32.35 | 41.82% | mixed | 100.00% | TOF |
| Rotes (2014)[19] | 1973-2012 | 278 | 7.30 | 31.40 | 46.76% | mixed | 100.00% | TOF |
| Bokma (2015)[20] | 1986-2013 | 153 | 9.60 | 30.80 | 37.91% | homograft | 100.00% | TOF |
| Escarain (2015)[21] | 1995-2012 | 263 | 7.50 | 42.00 | 28.14% | homograft | 7.98% | ROSS |
| Oliver (2015)[22] | 1990-2013 | 114 | 7.35 | 23.35 | 40.35% | mixed | 100.00% | TOF |
| Dunne (2016)[23] | 2000-2014 | 114 | 5.93 | 22.41 | 38.60% | xenograft | 88.60% | TOF |
| Kwak (2016)[24] | 1998-2014 | 108 | 7.73 | 19.30 | NA | xenograft | 100.00% | TOF |
| Haas (2018)[25] | 2010-2015 | 166 | 2.81 | 15.90 | 40.96% | mixed | NA | TOF |
| Li (2018)[26] | 2011-2014 | 30 | 2.01 | 21.45 | 46.67% | NA | 100.00% | TOF |
| Gentian Lluri (2018)[27] | 2010-2016 | 134 | 2.55 | 23.30 | 46.27% | xenograft | NA | TOF |
| He (2019)[28] | 2014-2018 | 41 | 2.81 | 21.00 | 56.10% | mixed | 100.00% | TOF |
| Marathe (2019)[29] | 2000-2017 | 215 | 7.70 | 13.20 | 38.14% | mixed | NA | TOF |
| Wijayarathne (2019)[30] | 1993-2017 | 150 | 3.11 | 33.82 | 43.33% | mixed | 100.00% | TOF |
| Bove (2020)[31] | 1991-2019 | 137 | 10.08 | 7.64 | 29.20% | homograft | 59.85% | ROSS |
| Feins (2020)[32] | 2009-2019 | 23 | 3.88 | 2.08 | 26.09% | xenograft | NA | TOF |
| Lee(2020)[33] | 1998-2015 | 190 | 9.84 | 18.54 | 40.53% | xenograft | 100.00% | TOF |
| Patel(2020)[34] | 1998-2016 | 30 | 4.92 | 4.10 | 40.00% | mixed | NA | ROSS |
| Pragt(2020)[35] | 2003-2013 | 45 | 5.80 | 31.26 | 55.56% | xenograft | 97.78% | TOF |
| Benjacholamas(2008)[36] | 1996-2005 | 24 | 3.63 | 0.57 | 58.33% | homograft | 0.00% | TA |
| Fiore(2008)[37] | 1995-2006 | 82 | 3.63 | 22.23 | 52.44% | mixed | NA | NA |
| Tokunaga(2008)[38] | 1977-2004 | 24 | 12.10 | 27.50 | 41.67% | mixed | 66.67% | TOF |
| Dave(2011)[39] | 2001-2007 | 170 | 5.36 | 11.14 | 45.29% | xenograft | 67.65% | ROSS |
| Chen(2013)[40] | 1992-2008 | 161 | 3.75 | 19.56 | 34.16% | xenograft | NA | TOF |
| Voges(2013)[41] | 2006-2008 | 26 | 3.63 | 14.30 | 42.31% | xenograft | NA | TOF |
| Ramanan(2015)[42] | 2002-2012 | 115 | 4.38 | 37.00 | 37.39% | xenograft | NA | ROSS |
| Shinkawa(2015)[43] | 1992-2013 | 123 | 7.55 | 13.00 | 44.72% | xenograft | 100.00% | TOF |
| Mitropoulos(2017)[44] | 2005-2013 | 99 | 3.60 | 38.00 | 28.28% | xenograft | 100.00% | TOF |
| Seilani(2017)[45] | 2001-2012 | 189 | 10.42 | 19.44 | 55.03% | xenograft | NA | TOF |
| Bell(2018)[46] | 1995-2015 | 188 | 7.26 | 15.13 | 43.09% | mixed | NA | TOF |
| Robichaud(2018)[47] | 1975-2016 | 924 | 5.15 | 5.96 | 36.36% | mixed | NA | TOF |
| Cocomello(2019)[48] | 1995-2018 | 209 | 6.13 | 24.67 | 43.54% | mixed | 100.00% | TOF |
| Nichay(2020)[49] | 2002-2017 | 58 | 4.25 | 10.88 | 36.21% | xenograft | 55.17% | ROSS |
| Spigel(2020)[50] | 2008-2019 | 79 | 3.21 | 19.50 | 48.10% | xenograft | 81.01% | TOF |
| Wasilewski(2020)[51] | 2012-2018 | 27 | 4.46 | 29.00 | 48.15% | xenograft | 100.00% | TOF |
| Al-Halees(2002)[52] | 1990-2000 | 53 | 4.00 | 8.00 | 45.28% | homograft | 54.72% | ROSS |
| Sievers(2003)[53] | 1994-2002 | 245 | 2.45 | 45.70 | 22.04% | homograft | 9.39% | ROSS |
| Matalanis(2004)[54] | 1994-2002 | 31 | 2.40 | 42.00 | 12.90% | homograft | 9.68% | ROSS |
| Raja(2004)[55] | 1996-2003 | 38 | 2.77 | 13.10 | 26.32% | mixed | 84.21% | ROSS |
| Williams(2005)[56] | 1991-2004 | 27 | 6.22 | 0.54 | 22.22% | homograft | 96.30% | ROSS |
| Alessandro(2008)[57] | 1994-2007 | 110 | 6.83 | 30.20 | 19.09% | homograft | 10.91% | ROSS |
| Morales(2008)[58] | 1996-2006 | 55 | 3.00 | 6.80 | NA | homograft | 76.36% | ROSS |
| Brown(2011)[59] | 1994-2010 | 230 | 7.80 | 42.20 | 36.09% | homograft | 24.35% | ROSS |
| Ryan(2011)[60] | 1994-2008 | 160 | 5.37 | 42.00 | 26.88% | homograft | NA | ROSS |
| Xu(2014)[61] | 1994-2009 | 58 | 8.20 | 28.30 | 27.59% | homograft | NA | ROSS |
| Christ(2017)[62] | 1995-2002 | 45 | 14.00 | 39.60 | 15.56% | mixed | 0.00% | ROSS |
| Martin(2017)[63] | 1990-2014 | 310 | 12.89 | 40.80 | 39.68% | homograft | 15.16% | ROSS |
| Pardo González(2017)[64] | 1997-2009 | 107 | 10.65 | 30.00 | 30.84% | homograft | 22.43% | ROSS |
| Alassas(2018) [65] | 1990-2015 | 41 | 7.42 | 27.00 | 36.59% | homograft | 100.00% | ROSS |
| David(2019)[66] | 1990-2004 | 212 | 17.93 | 34.00 | 33.96% | homograft | 20.28% | ROSS |
| Guerreiro(2019)[67] | 1992-1999 | 56 | 20.71 | 44.00 | 44.64% | homograft | NA | ROSS |
| Riggs(2020)[68] | 2005-2018 | 40 | 3.50 | 17.31 | 30.00% | homograft | 57.50% | ROSS |
| Sharifulin(2020)[69] | 1998-2015 | 244 | 5.99 | 43.30 | 26.64% | mixed | 1.23% | ROSS |
| Skillington(2013)[70] | 1992-2012 | 310 | 9.40 | 39.30 | 30.32% | NA | 9.68% | ROSS |
| Salehi(2007)[71] | 2001-2004 | 80 | 1.42 | 27.60 | NA | homograft | 12.50% | ROSS |
| Juthier(2012)[72] | 1992-2010 | 336 | 6.20 | 29.40 | 21.13% | mixed | 41.07% | ROSS |
| Mastrobuoni(2016)[73] | 1991-2014 | 306 | 9.86 | 41.70 | 25.16% | homograft | 9.15% | ROSS |
| Miskovic(2016)[74] | 1996-2014 | 209 | 7.90 | 43.00 | 23.92% | mixed | 8.13% | ROSS |
| Schneider(2017)[75] | 1994-2016 | 154 | 10.70 | 13.01 | 25.32% | mixed | 66.23% | ROSS |
| Van Dijck(2015)[76] | 1989-2013 | 570 | 6.52 | 13.79 | 32.63% | mixed | NA | TOF |
| Urso(2011)[77] | 1989-2003 | 347 | 5.90 | 13.85 | 36.89% | mixed | 46.97% | TOF |
| Ugaki(2015)[78] | 2000-2012 | 379 | 3.54 | 5.11 | 41.42% | mixed | 65.44% | PA |
| Tweddell(2000)[79] | 1985-1999 | 205 | 3.60 | 6.90 | NA | homograft | 66.34% | TOF |
| Tierney(2005)[80] | 1989-2003 | 98 | 5.10 | 3.18 | NA | homograft | NA | TOF |
| Thompson(2001)[81] | 1992-1999 | 65 | 2.88 | 0.03 | 40.00% | homograft | 0.00% | TA |
| Sinzobahamvya(2008)[82] | 1987-2007 | 35 | 6.17 | 0.06 | 45.71% | mixed | 2.86% | TA |
| Sinzobahamvya(2007)[83] | 2002-2006 | 38 | 1.83 | 0.44 | NA | mixed | NA | PA |
| Shih(2010)[84] | 1998-2008 | 98 | 2.69 | 2.90 | 35.71% | homograft | NA | ROSS |
| Shebani(2006)[85] | 2000-2003 | 62 | 1.23 | 2.54 | 46.77% | xenograft | 19.35% | PA |
| Sharma(2018)[86] | 2010-2015 | 100 | 2.63 | 12.00 | 46.00% | xenograft | 94.00% | NA |
| Sfyridis(2011)[87] | 1999-2010 | 34 | 7.10 | 10.90 | 26.47% | xenograft | 82.00% | TOF |
| Sekarski(2007)[88] | 1999-2005 | 133 | 2.68 | 3.22 | NA | xenograft | NA | TOF |
| Schubmehl(2017)[89] | 2000-2010 | 100 | 6.90 | 24.28 | 41.00% | xenograft | 100.00% | TOF |
| Schoenhoff(2011)[90] | 1999-2008 | 84 | 3.79 | 12.00 | NA | xenograft | NA | TOF |
| Bielefeld(2001)[91] | 1985-1999 | 223 | 6.00 | 2.80 | 47.53% | homograft | 0.00% | TOF |
| Schiralli(2011)[92] | 2011-2009 | 218 | 3.26 | 14.30 | 41.74% | xenograft | 83.49% | TOF |
| Saritas(2017)[93] | 2009-2016 | 76 | 5.00 | 11.40 | 55.26% | xenograft | 100.00% | TOF |
| Sarikouch(2016)[94] | 2005-2015 | 93 | 4.60 | 15.60 | 37.63% | homograft | 82.80% | TOF |
| Ruzmetov(2012)[95] | 2000-2005 | 100 | 5.76 | 18.60 | 42.00% | homograft | NA | ROSS |
| Ruzmetov(2013)[96] | 2000-2011 | 49 | 4.00 | 22.40 | 40.82% | xenograft | 100.00% | TOF |
| Rastan(2006)[97] | 2000-2005 | 72 | 2.58 | 9.20 | 52.78% | xenograft | 77.78% | TOF |
| Prior(2011)[98] | 1999-2009 | 193 | 4.60 | 6.70 | 43.52% | xenograft | 71.50% | TOF |
| Pragt(2017)[99] | 1965-2014 | 364 | 4.68 | 27.16 | 36.54% | mechanical | 97.25% | TOF |
| Perri(2012)[100] | 2006-2010 | 93 | 1.13 | 2.89 | 49.46% | xenograft | 60.22% | TO |
| Bibevski(2017)[101] | 2000-2010 | 287 | 5.02 | 15.28 | 38.68% | homograft | 40.77% | ROSS |
| Pawelec-Wojtalik(2005)[102] | 1999-2004 | 43 | 2.01 | 2.88 | 39.53% | xenograft | 30.23% | ROSS |
| Ong(2013)[103] | 1974-2011 | 49 | 19.35 | 5.42 | 36.73% | mixed | 79.59% | TOF |
| Nordmeyer(2009)[104] | 2004-2007 | 60 | 3.33 | 21.00 | 58.33% | homograft | 90.00% | TOF |
| Niemantsverdriet(2008)[105] | 1987-2003 | 194 | 4.94 | 1.63 | 48.97% | mixed | 41.75% | TOF |
| Morales(2006)[106] | 2001-2005 | 76 | 1.64 | 2.40 | NA | xenograft | 80.26% | PA |
| Morales(2007)[107] | 2002-2005 | 26 | 1.55 | 20.30 | 30.77% | xenograft | 100.00% | TOF |
| Mokhles(2011)[108] | 1986-2009 | 463 | 9.00 | 19.00 | 41.04% | homograft | NA | ROSS |
| Meyns(2004)[109] | 2000-2002 | 57 | 1.89 | 9.00 | NA | xenograft | 53.45% | TOF |
| Batlivala(2012)[110] | 1990-2009 | 254 | 4.73 | 15.60 | 35.83% | mixed | 93.70% | TOF |
| Mercer(2018)[111] | 2004-2014 | 26 | 2.70 | 0.40 | 42.31% | homograft | NA | TA |
| Alsoufi(2009)[112] | 1992-2005 | 36 | 8.30 | 2.81 | 41.67% | homograft | 77.78% | TGA |
| Amalia(2011)[113] | 1989-2008 | 156 | 7.39 | 0.05 | NA | homograft | 0.00% | TA |
| Louis(2018)[114] | 1992-2013 | 54 | 9.44 | 0.06 | 50.00% | homograft | 0.00% | TA |
| Alsoufi(2010)[115] | 1991-2007 | 151 | 6.14 | 9.27 | 35.10% | homograft | 68.21% | ROSS |
| Martin(2014)[116] | 1991-2011 | 246 | 9.00 | 28.60 | 22.36% | homograft | 25.20% | ROSS |
| Bansal(2015)[117] | 1992-2012 | 305 | 9.22 | 13.10 | 30.82% | homograft | 56.72% | ROSS |
| Bechtel(2008)[118] | 2000-2002 | 72 | 4.39 | 43.40 | 22.22% | homograft | NA | ROSS |
| Boethig(2007)[119] | 1985-2004 | 188 | 5.67 | 24.80 | 29.79% | homograft | 43.09% | ROSS |
| Boethlg(2009)[120] | 2003-2008 | 60 | 2.90 | 26.00 | 51.56% | xenograft | 90.63% | TOF |
| Sander(2011)[121] | 1994-2009 | 40 | 8.84 | 0.38 | 32.50% | homograft | NA | TA |
| Breymann(2009)[122] | 1999-2006 | 165 | 4.15 | 3.90 | 52.73% | xenograft | 50.30% | TOF |
| Brown(2010)[123] | 2000-2005 | 1588 | 3.76 | 20.54 | 31.80% | homograft | 20.47% | ROSS |
| Brown(2011)[124] | 1999-2010 | 216 | 4.84 | 9.10 | 44.44% | mixed | 56.94% | TA |
| Brown(2005)[125] | 1985-2003 | 117 | 6.10 | 4.30 | 51.28% | homograft | 58.97% | TA |
| Burch(2010)[126] | 2000-2005 | 94 | 5.34 | 9.93 | NA | homograft | 74.47% | NA |
| Chen(2019)[127] | 2008-2018 | 128 | 3.00 | 2.08 | NA | xenograft | 23.44% | PA |
| Christenson(2010)[128] | 1993-2009 | 205 | 7.82 | 5.74 | 48.78% | mixed | 40.49% | TOF |
| Cleuziou(2016)[129] | 1994-2013 | 152 | 9.99 | 1.53 | NA | homograft | 74.00% | TOF |
| Concha(2003)[130] | 1992-2002 | 169 | 3.01 | 29.93 | 20.71% | homograft | 36.09% | ROSS |
| Corno(2017)[131] | 2012-2016 | 71 | 2.08 | 24.00 | NA | xenograft | 100.00% | NA |
| Dekens(2019)[132] | 1990-2016 | 197 | 8.50 | 9.00 | 39.09% | homograft | NA | TOF |
| Dohmen(2007)[133] | 2000-2003 | 23 | 3.83 | 44.00 | 21.74% | mixed | 4.35% | ROSS |
| Elder(2013)[134] | 1991-2010 | 34 | 10.08 | 0.56 | 23.53% | homograft | 97.06% | ROSS |
| Etnel(2017)[135] | 1995-2017 | 260 | 8.60 | 29.43 | 29.62% | homograft | 21.15% | ROSS |
| Falchetti(2019)[136] | 1992-2014 | 53 | 10.08 | 0.04 | 43.40% | mixed | 0.00% | TA |
| Fiore(2011)[137] | 1999-2010 | 232 | 4.00 | 8.00 | NA | xenograft | 55.17% | TOF |
| Fiore(2010)[138] | 1998-2009 | 84 | 4.97 | 0.70 | 55.95% | mixed | 35.71% | TA |
| Francoisa(2018)[139] | 1993-2015 | 88 | 7.60 | 2.20 | 40.86% | mixed | 60.22% | ROSS |
| Fullerton(2003)[140] | 1997-2002 | 44 | 3.17 | 49.00 | 38.64% | homograft | NA | ROSS |
| Giamberti(2013)[141] | 2001-2011 | 76 | 4.33 | 36.00 | 38.16% | xenograft | 100.00% | TOF |
| Gröning(2019)[142] | 1977-2016 | 322 | 7.58 | 18.23 | 50.93% | mixed | NA | TOF |
| Holmes(2012)[143] | 2002-2009 | 45 | 4.20 | 3.50 | 38.78% | xenograft | 34.69% | TOF |
| Homann(2000)[144] | 1974-1999 | 505 | 6.09 | 4.42 | NA | mixed | NA | PA |
| Hoxha(2016)[145] | 1991-2015 | 58 | 8.26 | 9.34 | 44.83% | mixed | 86.21% | TOF |
| Hunter(2016)[146] | 2000-2013 | 81 | 5.85 | 11.92 | 44.44% | mixed | NA | TOF |
| Jussli-Melchers(2019)[147] | 2009-2016 | 53 | 4.53 | 9.72 | NA | xenograft | 94.34% | TOF |
| Kalavrouziotis(2006)[148] | 1993-2005 | 29 | 6.17 | 0.10 | 62.07% | mixed | 0.00% | TAC |
| Kalfa(2011)[149] | 1993-2009 | 107 | 5.70 | 22.00 | 26.17% | homograft | 39.25% | ROSS |
| Kalfa(2012)[150] | 1993-2010 | 115 | 5.30 | 5.48 | 54.78% | homograft | NA | PA |
| Kallio(2015)[151] | 1994-2009 | 51 | 11.65 | 5.14 | 39.22% | homograft | 80.39% | ROSS |
| Kanter(2002)[152] | 1989-2000 | 93 | 4.90 | 10.10 | NA | mixed | 100.00% | TOF |
| Kanter(2003)[153] | 1998-2002 | 56 | 2.50 | 11.80 | NA | xenograft | 98.21% | TOF |
| Karaskov(2016)[154] | 1998-2014 | 741 | 5.80 | 47.40 | 24.97% | mixed | 5.80% | ROSS |
| Kim(2001)[155] | 1997-2000 | 50 | 2.04 | 3.32 | NA | xenograft | 84.00% | PA |
| Konertz(2011)[156] | 2006-2008 | 61 | 2.58 | 9.79 | 39.34% | xenograft | 50.82% | TOF |
| Kuo(2019)[157] | 2002-2015 | 166 | 5.44 | 11.64 | 42.77% | xenograft | 96.99% | NA |
| Lo Rito(2014)[158] | 1991-2011 | 140 | 10.82 | 10.59 | 35.71% | homograft | 57.14% | ROSS |
| McBrien(2012)[159] | 1997-2010 | 101 | 4.70 | 24.80 | 31.68% | homograft | 48.51% | ROSS |
| Mokhles(2013)[160] | 1988-2011 | 1775 | 7.20 | 43.70 | 25.30% | mixed | 9.07% | ROSS |
| Mookhoek(2015)[161] | 1990-2013 | 76 | 4.26 | 0.27 | 21.05% | mixed | 76.32% | ROSS |
| Nelson(2015)[162] | 1991-2013 | 240 | 11.27 | 8.36 | 29.58% | homograft | 73.75% | ROSS |
| Palma(2011)[163] | 2000-2008 | 156 | 4.83 | 5.40 | 45.51% | xenograft | 62.82% | TOF |
| Pasquali(2007)[164] | 1995-2004 | 121 | 5.66 | 14.39 | 32.23% | homograft | 75.21% | ROSS |
| Perri(2015)[165] | 2000-2014 | 53 | 3.22 | 4.03 | 50.94% | homograft | NA | PA |
| Ruffer(2012)[166] | 2000-2010 | 63 | 3.50 | 15.91 | 34.92% | xenograft | 82.54% | PA |
| Ruzmetov(2015)[167] | 1993-2012 | 136 | 10.60 | 10.60 | 41.91% | homograft | 77.21% | ROSS |
| Sandica(2016)[168] | 1985-2012 | 711 | 5.16 | 14.34 | 42.76% | mixed | 69.48% | TOF |
| Schmid(2002)[169] | 1997-2002 | 51 | 3.04 | 43.15 | 31.37% | mixed | NA | ROSS |
| Takkenberg(2002)[170] | 1988-2000 | 343 | 4.00 | 26.00 | 32.36% | homograft | 43.15% | NA |
| Yang(2010)[171] | 1997-2007 | 45 | 4.12 | 1.67 | 35.56% | homograft | 77.78% | TOF |
| Yuen(2019)[172] | 2004-2016 | 82 | 4.45 | 28.70 | 35.37% | xenograft | 100.00% | TOF |
| Zhang(2017)[173] | 2002-2013 | 53 | 6.62 | 3.50 | 37.74% | xenograft | 58.49% | PA |
| Zimmermann(2018)[174] | 1993-2011 | 100 | 5.68 | 16.77 | 24.00% | mixed | 60.00% | ROSS |
| Zubairi(2011)[175] | 1987-2007 | 169 | 8.00 | 15.40 | 44.97% | mixed | 100.00% | TOF |
| Cho(2002)[176] | 1977-2000 | 335 | 11.40 | 11.30 | 52.24% | mixed | 71.34% | TOF |
| Christ(2019)[177] | 2002-2010 | 492 | 7.70 | 56.70 | 23.17% | mixed | NA | ROSS |
| Erez(2006)[178] | 2002-2005 | 31 | 1.08 | 14.50 | NA | xenograft | 100.00% | TOF |
| Lee(2012)[179] | 1998-2011 | 170 | 5.98 | 2.00 | 39.41% | xenograft | 100.00% | TOF |
| Pratap(2010)[180] | 2000-2009 | 34 | 1.92 | 3.20 | 38.24% | xenograft | NA | TOF |
| Allen(2002)[181] | 1993-1999 | 48 | 3.58 | 11.00 | NA | xenograft | 97.92% | TOF |
| Kumar(2005)[182] | 1993-2003 | 153 | 6.42 | 28.00 | 32.68% | homograft | NA | ROSS |
| Adamson(2019)[183] | 2010-2017 | 54 | 4.59 | 18.00 | 48.15% | xenograft | 100.00% | TOF |
| Albanesi(2014)[184] | 1999-2010 | 106 | 7.56 | 17.40 | 29.25% | xenograft | NA | ROSS |
| Baskett(2003)[185] | 1990-2001 | 83 | 4.58 | 5.10 | 46.99% | homograft | 65.06% | PA |
| Buchholz(2016)[186] | 2000-2015 | 52 | 7.90 | 25.92 | 44.23% | xenograft | 84.62% | TOF |
| Danton(2001)[187] | 1988-2000 | 38 | 4.17 | 0.13 | 50.82% | mixed | 5.26% | TA |
| Fan(2017)[188] | 2007-2015 | 40 | 5.38 | 1.65 | 60.00% | xenograft | 0.00% | PA |
| Hartz(2003)[189] | 1999-2002 | 47 | 1.83 | 14.20 | 34.04% | xenograft | 100.00% | TOF |
| Horer(2007)[190] | 1977-2004 | 39 | 9.63 | 6.51 | 46.15% | mixed | 58.97% | TGA |
| Javadpour(2002)[191] | 1990-2000 | 60 | 3.25 | 6.47 | 43.33% | homograft | 65.00% | PA |
| Raju(2015)[192] | 1997-2013 | 26 | 3.79 | 5.30 | 38.46% | mixed | 57.69% | TGA |
| Sievers(2016)[193] | 1990-2013 | 1779 | 8.30 | 44.70 | 24.73% | homograft | 7.81% | ROSS |
| Talwar(2012)[194] | 1992-2009 | 36 | 7.92 | 11.30 | 8.33% | homograft | NA | ROSS |
| Vitanova(2014)[195] | 1994-2011 | 145 | 8.42 | 0.32 | NA | mixed | 44.14% | PA |
| Timo(2014)[196] | 1995-2012 | 645 | 8.40 | 42.30 | 24.50% | mixed | 14.11% | ROSS |
| Dang van(2021)[197] | 2000-2017 | 107 | 4.7 | 26.1 | 44.86% | homograft | 94.39% | TOF |
| Schlein(2021)[198] | 1991-2020 | 124 | 10.98 | 10.61 | 29.03% | mixed | 66.13% | ROSS |
| Ghiselli(2021)[199] | 2007-2019 | 85 | 4.8 | 26.70 | 35.29% | xenograft | 94.12% | TOF |
| Romeo(2021)[200] | 1991-2018 | 1431 | 9.13 | 47.70 | 25.72% | homograft | NA | ROSS |
| Lenoir(2021)[201] | 2010-2018 | 86 | 4.52 | 36.18 | 37.21% | Homograft | NA | TOF |
| Neil(2020)[202] | 2000-2018 | 75 | 10.6 | 0.12 | 54.67% | Mixed | NA | TA |
| Hoashi(2021)[203] | 2013-2019 | 178 | 3.17 | 1.36 | 50.56% | Xenograft | NA | PA |
| Bove(2021)[204] | 1991-2019 | 137 | 9.92 | 7.88 | 29.20% | Homograft | 67.88% | ROSS |
| Chauvette(2020)[205] | 2000-2019 | 31 | 2.93 | 43 | 16.13% | Homograft | 19.35% | ROSS |
| Fernandez-Carbonell(2021)[206] | 1997-2017 | 142 | 14.9 | 28 | 30.99% | Homograft | 23.94% | ROSS |
| Murin(2021)[207] | 2013-2019 | 50 | 2.69 | 7.15 | 42% | Mixed | 80% | ROSS |
| Georgiev(2020)[208] | 2006-2018 | 211 | 6.4 | 19.15 | NA | Mixed | 100% | NA |
| Arribas-Leal(2021)[209] | 1986-2019 | 21 | 1.45 | 31 | 33.33% | Xenograft | 85.71% | TOF |
| Callahan(2021)[210] | 2002-2016 | 355 | 4.48 | 4.48 | 49.58% | Mixed | 100% | TA |
| Maeda(2021)[211] | 2002-2019 | 203 | 6.39 | 24.51 | NA | Xenograft | NA | TOA |
| Naimo(2021)[212] | 1979-2018 | 255 | 17 | 0.37 | 45.49% | NA | 7.84% | TA |
| Raja(2021)[213] | 2012-2017 | 98 | 4.2 | 7.95 | 39.80% | Homograft | NA | PA |
| Selcuk(2021)[214] | 2018-2019 | 48 | 1.00 | 3.24 | NA | Xenograft | 60.42% | PA |
| Dorobantu(2020)[215] | 2000-2013 | 707 | 3.64 | 28.26 | 45.54% | NA | 100% | TOF |
| Kim(2021)[216] | 2004-2017 | 131 | 7.4 | 21.64 | 31.30% | Mixed | 100% | TOF |
| Tominaga(2021)[217] | 2003-2019 | 46 | 6.46 | 35.94 | 52.17% | Mixed | 100% | TOF |
| Yasukawa(2021)[218] | 2010-2019 | 30 | 2.76 | 32.71 | 33.33% | Xenograft | 100% | TOF |

Supplementary Table 3: Pooled Kaplan-Meier estimates for probability of survival and freedom from reintervention

|  |  | Overall | Ross | TOF | TA | Infants | Children | Homograft | Xenograft |
| --- | --- | --- | --- | --- | --- | --- | --- | --- | --- |
| Survival | 5-year | 92.6% | 94.8% | 94.9% | 82.3% | 84.8% | 86.5% | 93.1% | 89.5% |
|  | 10-year | 89.7% | 91.5% | 90.7% | 81.1% | 82.3% | 85.8% | 90.8% | 87.8% |
|  | 15-year | 86.0% | 87.3% | 87.8% | 79.0% | 80.3% | 83.3% | 86.8% | 84.6% |
| Reintervention | 5-year | 90.4% | 95.7% | 91.7% | 44.3% | 52.0% | 83.2% | 92.4% | 88.1% |
|  | 10-year | 78.9% | 90.2% | 77.1% | 22.5% | 29.8% | 67.1% | 83.5% | 72.2% |
|  | 15-year | 67.6% | 83.3% | 55.4% | —— | 11.9% | 40.1% | 73.9% | 49.2% |

TOF: Tetralogy of Fallot; TA: truncus arteriosus.

Supplementary Table 4: Baseline characteristics for TOF and TA subgroups

| Characteristics | | Pooled estimates (range or mean ± SD) | |
| --- | --- | --- | --- |
|  |  | TOF, N=20 | TA, N=9 |
| Age(years) | | 24.95±11.62 | 0.19±0.92 |
| Female (%) | | 43.74(28.28-56.10) | 48.17(40.00-62.07) |
| Implantation period | | 2002(1973-2019) | 1999(1979-2018) |
| Preoperative NYHA III-IV (%) | | 25.44(6.32-43.88) | —— |
| Patients with previous cardiac procedures (%) | | 96.90(71.34-100.00） | 4.00(0-7.84) |
| Patients with concomitant procedures (%) | | 68.63(20.26-100) | —— |
| Conduit diameter (mm) | | 25.24±2.28 | 11.87±2.80 |
| Follow-up (years) | | 6.22±4.45 | 10.42±6.74 |
| RVOT grafts | homograft (%) | 27.90 | 78.99 |
|  | Xenograft (%) | 66.78 | 20.80 |
|  | Mechanical valves (%) | 2.97 | —— |

SD: standard deviation; TOF: Tetralogy of Fallot; TA: truncus arteriosus; NYHA: New York heart association.

Supplementary Table 5: Baseline characteristics for xenograft group and Contegra® (bovine jugular vein) conduit group

| Characteristics | | Pooled estimates (range or mean ± SD) | |
| --- | --- | --- | --- |
|  |  | All xenograft (N=69) | Contegra® conduit (N=36) |
| Age(years) | | 14.26±9.76 | 7.41±8.20 |
| Female (%) | | 42.4(26.1-60.0) | 45.4(26.5-61.5) |
| Patients with previous cardiac procedures (%) | | 80.4(0.0-100.0) | 62.4 (0-100) |
| Conduit diameter (mm) | | 22.57±2.91 | 18.46 ±4.08 |
| Follow-up (years) | | 4.50±2.70 | 4.29±2.95 |
| Etiology  [n (%)] | AVD(ROSS) | 384(6.2) | 327(10.0) |
|  | TOF | 3251(52.8) | 1018(31.1) |
|  | TA | 475(7.7) | 477(14.6) |
|  | PA | 709(11.5) | 524(16.0) |
|  | TGA | 287(4.7) | 248(7.6) |
|  | Others** | 1056(17.1) | 678(20.7) |

Supplementary Table 6: Comparison between pooled outcomes for all xenograft studies and studies with Contegra® conduit implantation

|  | | All xenograft (N=69) | | | Contegra® conduit (N=36) | | |
| --- | --- | --- | --- | --- | --- | --- | --- |
| Outcomes | | Estimate  (95%CI) | N | I^2^ | Estimate  (95%CI) | N | I^2^ |
| Early mortality (%) | All cause | 3.00(2.34, 3.85) | 65 | 50.0% | 4.19 (2.86, 6.13) | 32 | 70.3% |
|  | Cardiac | 2.45 (1.84, 3.26) | 58 | 37.7% | 3.07 (1.74, 5.39) | 24 | 71.2% |
| Re-exploration for bleeding (%) | | 5.66(4.20, 7.62) | 16 | 24.4% | 5.06 (2.27, 11.28) | 11 | 82.84% |
| Late mortality(%/y) | All cause | 0.68(0.51, 0.90) | 63 | 53.9% | 1.10 (0.78, 1.54) | 30 | 53.3% |
|  | Cardiac | 0.54(0.39, 0.74) | 52 | 20.0% | 0.56(0.37, 0.86) | 22 | 0.0% |
| Reintervention(%/y) | | 3.47(2.70, 4.46) | 66 | 92.4% | 5.74 (4.38, 7.52) | 32 | 91.1% |
| Endocarditis(%/y) | | 0.80(0.60,1.09) | 39 | 56.3% | 1.17 (0.86, 1.59) | 25 | 54.7% |
| Dysfunction (%/y) | | 2.46(1.30, 4.68) | 13 | 91.1% | 5.46(2.96, 10.06) | 7 | 89.5% |

Supplementary Table 7: Baseline characteristics for aortic valve disease patients older than 16 years old

| Characteristics | Pooled estimates (range or mean ± SD) | |
| --- | --- | --- |
|  | Aortic valve disease: Ross procedure | |
|  | Homograft, N=14 | Xenograft , N=5 |
| Age(years) | 43.11±11.45 | 51.2 ±10.7 |
| Female (%) | 27.2(12.9-44.6) | 23.0(15.6-23.2) |
| Implantation period | 2001(1988-2015) | 2008(1995-2015) |
| Preoperative NYHA III-IV (%) | 31.1(17.0-61.3) | 67.3(4.0-68.9) |
| Patients with previous cardiac procedures (%) | 10.0(0-24.3） | 2.4(0-2.5) |
| Patients with concomitant procedures (%) * | 41.8 (17.6-54.8) | 31.1(30.4-31.1) |
| Conduit diameter (mm) | 26.89±2.13 | 22.10±2.72 |
| Follow-up (years) | 8.52 ± 5.36 | 6.77±3.66 |

Supplementary Table 8: Pooled results for aortic valve disease patients older than 16 years old

|  | | Aortic valve disease: homograft (N=14) | | | Aortic valve disease: xenograft (N=5) | | |
| --- | --- | --- | --- | --- | --- | --- | --- |
| Outcomes | | Estimate  (95%CI) | N | I^2^ | Estimate  (95%CI) | N | I^2^ |
| Early mortality (%) | All cause | 1.81 (1.18, 2.77) | 11 | 35.17% | 3.25 (2.26, 4.67) | 2 | 46.43% |
|  | Cardiac | 1.08 (0.61, 1.89) | 9 | 0.00% | —— | 0 | —— |
| Re-exploration for bleeding (%) | | 7.45 (3.68, 15.23) | 6 | 75.20% | 8.41(6.30, 11.21) | 2 | 0.46% |
| Late mortality(%/y) | All cause | 0.70(0.61, 0.80) | 11 | 0.00% | 0.73 (0.12, 4.34) | 2 | 95.30% |
|  | Cardiac | 0.33(0.19, 0.56) | 9 | 45.03% | 0.44(0.28, 0.71) | 2 | 33.02% |
| Reintervention(%/y) | | 0.52(0.42, 0.63) | 14 | 27.73% | 0.70 (0.17, 2.83) | 4 | 96.96% |
| Endocarditis(%/y) | | 0.13(0.10, 0.17) | 11 | 0.00% | 0.16 (0.09, 0.29) | 4 | 11.15% |
| Dysfunction (%/y) | | 1.09(0.62, 1.90) | 7 | 73.98% | —— | 1 | —— |

Supplementary Table 9: Baseline characteristics for right-sided conduit RVOTVS with homograft and xenograft

| Characteristics | | Pooled estimates (range or mean ± SD) | |
| --- | --- | --- | --- |
|  |  | Right-sided conduit | |
|  |  | Homograft(>90%), N=19 | Xenograft (>90%), N=53 |
| Age(years) | | 9.35±7.40 | 15.11±9.62 |
| Female (%) | | 44.85(35.56-58.33) | 42.73(26.09-60) |
| Implantation period | | 2000(1985-2018) | 2007(1986-2019) |
| Preoperative NYHA III-IV (%) | | —— | 23.82(6.32-100) |
| Patients with previous cardiac procedures (%) | | 54.80(0-100） | 83.48(0-100) |
| Patients with concomitant procedures (%) * | | 69.92(16.67-100) | 66.15(8.00-100) |
| Conduit diameter (mm) | | 19.03±2.64 | 22.94±2.72 |
| Follow-up (years) | | 5.82±3.60 | 4.65±2.87 |
| Etiology  [n (%)] | AVD(ROSS) | 25(1.29) | 82(1.71) |
|  | TOF | 772(39.79) | 2899(60.52) |
|  | TA | 522(26.91) | 336(7.01) |
|  | PA | 250(12.89) | 529(11.04) |
|  | TGA | 153(7.89) | 189(3.95) |
|  | PS/PR | 16(0.82) | 237(4.95) |
|  | DORV | 42(2.16) | 146(3.05) |
|  | Others** | 105(5.41) | 363(7.58) |

*Aortic valve procedures were excluded; **: others include absent pulmonary valve, endocarditis, rheumatic disease, redo RVOTVS (without original diagnosis), unknown causes, etc.

Supplementary Table 10: Pooled outcomes of right-sided conduit RVOTVS with homograft and xenograft

|  | | Right-sided conduit: homograft (N=19) | | | Right-sided conduit: xenograft (N=53) | | |
| --- | --- | --- | --- | --- | --- | --- | --- |
| Outcomes | | Estimate  (95%CI) | N | I^2^ | Estimate  (95%CI) | N | I^2^ |
| Early outcomes (%) | | | | |  | | |
| Early mortality | All cause | 8.22 (5.95, 11.34) | 17 | 64.62% | 2.38 (1.73, 3.28) | 49 | 50.81% |
|  | Cardiac | 5.84 (3.72, 9.16) | 13 | 63.19% | 2.01 (1.43, 2.83) | 44 | 29.94% |
|  | Valve-related | 0.89 (0.42, 1.92) | 12 | 0.00% | 0.75 (0.49, 1.14) | 41 | 0.00% |
|  | SUD | 0.67 (0.29, 1.55) | 11 | 0.00% | All zero | 38 | 0.00% |
| Early PMI/ICD | | 10.01 (4.73, 21.20) | 2 | 76.11% | 4.30(3.13, 5.90) | 15 | 5.77% |
| Re-exploration for bleeding | | 9.05 (6.23, 13.14) | 2 | 37.80% | 5.00(3.57, 7.02) | 12 | 15.75% |
| Early stroke | | —— | 0 | —— | 1.38(0.71, 2.67) | 10 | 0.00% |
| Early TE/VT | | —— | 0 | —— | 1.91(0.68, 5.36) | 9 | 59.05% |
| Early MI | | —— | 1 | —— | 1.89(0.76, 4.72) | 2 | 0.00% |
| AKI | | —— | 0 | —— | 6.65(2.75, 16.11) | 5 | 47.07% |
| Late outcomes (%/y) | | | | | | | |
| Late mortality | All cause | 1.14(0.78, 1.65) | 18 | 69.26% | 0.63(0.46, 0.87) | 48 | 50.82% |
|  | Cardiac | 0.58(0.37, 0.92) | 14 | 43.47% | 0.52(0.35, 0.76) | 40 | 26.49% |
|  | Valve-related | 0.30 (0.19, 0.48) | 15 | 0.00% | 0.26(0.17, 0.40) | 39 | 0.00% |
|  | SUD | 0.25(0.15, 0.41) | 15 | 0.00% | 0.26(0.17, 0.40) | 39 | 0.00% |
| Late PMI/ICD | | —— | 1 | —— | 0.86(0.26, 2.87) | 5 | 59.25% |
| Late stroke | | —— | 0 | —— | 0.19(0.06, 0.66) | 3 | 31.01% |
| Late TE/VT | | —— | 0 | —— | 0.64(0.17, 2.34) | 8 | 73.62% |
| Late MI | | —— | 1 | —— | —— | 1 | —— |
| Overall (%/y) | | | | | | | |
| Reintervention | | 4.66(3.30, 6.60) | 17 | 92.01% | 3.23 (2.34, 4.47) | 40 | 91.51% |
| Reoperation | | 3.05 (2.07, 4.49) | 17 | 89.84% | 1.76 (1.34, 2.30) | 47 | 79.19% |
| Endocarditis | | 0.24(0.14, 0.41) | 9 | 0.00% | 0.69(0.46, 1.02) | 26 | 57.65% |
| Dysfunction | | 5.65(3.41, 9.38) | 6 | 90.99% | 2.55(1.56, 4.15) | 10 | 74.51% |
| Moderate to severe PS | | 2.01 (1.43, 2.84) | 3 | 34.43% | 2.13(1.38, 3.29) | 16 | 64.48% |
| Moderate to severe PR | | 1.81 (0.63, 5.26) | 6 | 90.99% | 2.89 (2.10, 3.97) | 33 | 84.34% |

Supplementary Table 11: Baseline characteristics for infants, children and adults subgroups

| Characteristics | | Pooled estimates (range or mean ± SD) | | |
| --- | --- | --- | --- | --- |
|  |  | Infants, N=11 | Children, N=35 | Adults, N=18 |
| Age(years) | | 0.15±0.02 | 4.74±4.06 | 45.07±10.54 |
| Female (%) | | 42.80(21.05-62.07) | 41.88(8.33-58.33) | 28.79(12.90-52.94) |
| Implantation period | | 2001(1987-2018) | 2003(1979-2019) | 2004(1990-2018) |
| Preoperative NYHA III-IV (%) | | —— | —— | 48.01(13.41-67.75) |
| Patients with previous cardiac procedures (%) | | 19.20(0-76.32） | 53.10(0-100) | 26.71(0.00-100.00) |
| Patients with concomitant procedures (%) * | | 99.81(97.14-100) | —— | 34.31(8.00-77.63) |
| Conduit diameter (mm) | | 11.96±1.97 | 17.71±3.11 | 26.10±1.88 |
| Follow-up (years) | | 7.35±4.46 | 7.33±6.68 | 7.98±5.73 |
| Etiology  [n (%)] | AVD(ROSS) | 88(11.49) | 1008(31.18) | 3961(89.31) |
|  | TOF | 49(6.40) | 629(19.46) | 405(9.13) |
|  | TA | 541(70.63) | 611(18.90) | 8(0.18) |
|  | PA | 56(7.31) | 381(11.78) | 23(0.52) |
|  | TGA | 6(0.78) | 254(7.86) | 8(0.18) |
|  | PS/PR | —— | 47(1.45) | 13(0.29) |
|  | DORV | —— | 99(3.06) | 4(0.09) |
|  | Others** | 26(3.39) | 204(6.31) | 13(0.03) |
| RVOT grafts | homograft (%) | 67.36 | 59.44 | 58.75 |
|  | Xenograft (%) | 32.25 | 40.56 | 40.89 |
|  | Mechanical valves (%) | 0 | 0 | 0.36 |

*Aortic valve procedures were excluded; **: others include absent pulmonary valve, endocarditis, rheumatic disease, redo PVR (without original diagnosis), unknown causes, etc.

Supplementary Table 12: Meta-regression for early and late mortality and overall reintervention, dysfunction and endocarditis within overall group

| **Covariates** | | **Outcome measurements** | | |
| --- | --- | --- | --- | --- |
|  |  | Early mortality  Coefficient (95%CI), P-value | Late mortality Coefficient (95%CI), P-value | Reintervention  Coefficient (95%CI), P-value |
| Age | | -0.037 (-0.044, -0.029), P<0.001 | -0.013(-0.023, -0.003), p=0.009 | -0.063 (-0.071, -0.054), p<0.001 |
| Study size | | -0.001(-0.001,0.000), P=0.006 | -0.000(-0.001, 0.001), p=0.836 | -0.001 (-0.002, -0.001), p<0.001 |
| Study design(retrospective) | | -0.137(-0.733,0.459), P=0.652 | -0.171 (-0.831, 0.490), P=0.612 | 0.742(0.104, 1.380), p=0.023 |
| Sex (female %) | | 1.662(0.152, 3.171), P=0.031 | 1.276(-0.128, 2.680), p=0.075 | 4.853(3.475, 6.231), p<0.001 |
| Follow-up | | —— | -0.084(-0.124, -0.043), p<0.001 | -0.092(-0.133, -0.051), p<0.001 |
| Mean implantation period | | -0.038(-0.063, -0.014), p=0.002 | -0.017 (-0.042, 0.007), p=0.168 | 0.007(-0.016, 0.031), p=0.543 |
| NYHA III-IV (%) | | 1.271(0.458,2.084), p=0.002 | 1.280(0.269, 2.290), P=0.013 | -0.829(-2.104, 0.447), p=0.203 |
| Conduit diameters (mm) | | -0.117(-0.143, -0.091), p<0.001 | -0.071(-0.103, -0.039), p<0.001 | -0.170(-0.193, -0.147), p<0.001 |
| Prior cardiac procedures (%) | | -0.760(-1.233, -0.286), p=0.002 | -0.243(-0.578, 0.091), p= 0.154 | 0.088(-0.401, 0.577), p=0.724 |
| Concomitant procedures (%) * | | 1.283(0.354, 2.211), p=0.007 | 0.833(-0.012, 1.678), p=0.053 | 1.383 (0.533, 2.233), p=0.001 |
| Surgical procedure (Ross) | | -0.301(-0.592, -0.010), p=0.043 | -0.337 (-0.633, -0.041), p=0.026 | -1.177(-1.449, -0.905), p<0.001 |
| RVOT grafts  (homograft) | Xenograft | -0.321(-0.668, 0.027), p=0.071 | -0.005(-0.346, 0.336), P=0.977 | 0.562(0.231, 0.893), p=0.001 |
|  | Mixed | -0.187(-0.542, 0.168), p=0.302 | 0.189(-0.128, 0.506), P=0.242 | 0.436(0.109, 0.764), p=0.009 |

Continue

| **Covariates** | | **Outcome measurements (overall)** | | |
| --- | --- | --- | --- | --- |
|  |  | Reoperation  Coefficient (95%CI), P-value | Dysfunction  Coefficient (95%CI), P-value | Endocarditis  Coefficient (95%CI), P-value |
| Age | | -0.055(-0.065,-0.045), p<0.001 | -0.046 (-0.065, -0.028), p<0.001 | -0.031(-0.043, -0.018), p<0.001 |
| Study size | | -0.001(-0.002,-0.000),p<0.001 | -0.003(-0.004, -0.002), p<0.001 | -0.001(-0.002, -0.001), p<0.001 |
| Study design(retrospective) | | 0.416(-0.312,1.144),p=0.263 | Only 9 prospective studies | 0.574(-0.293, 1.441), p=0.195 |
| Sex(female) | | 4.636(3.091, 6.180), p<0.001 | 5.923 (2.719, 9.128), p<0.001 | 2.107(-0.013, 4.226), p=0.051 |
| Follow-up | | -0.068(-0.114,-0.022),p=0.004 | -0.123(-0.188, -0.058), p<0.001 | -0.139(-0.197, -0.081), p<0.001 |
| Mean implantation period | | -0.012(-0.038,0.014),p=0.364 | 0.065(0.007, 0.122), p= 0.028 | 0.066(0.027, 0.104), p<0.001 |
| NYHA III-IV | | -0.152(-1.532,1.228),p=0.829 | -2.189(-4.431, 0.054), p= 0.056 | -0.326 (-2.427, 1.775), p=0.761 |
| Conduit diameters | | -0.151(-0.178,-0.125),p<0.001 | -0.129(-0.181, -0.077), p<0.001 | -0.067(-0.125, -0.009), p= 0.024 |
| Prior cardiac procedures | | 0.192(-0.362,0.747),p=0.496 | 0.498 (-0.404, 1.399), p= 0.279 | 0.978(0.267, 1.689), p=0.007 |
| Concomitant procedures* | | 0.487(-0.360,1.333),p=0.260 | 0.253(-1.200, 1.705), p=0.733 | 1.068(-0.487, 2.624), p=0.178 |
| Surgical procedure (Ross) | | -1.202(-1.510,-0.893),p<0.001 | -1.045(-1.628, -0.463), p<0.001 | -0.979(-1.358, -0.600), p<0.001 |
| RVOT grafts  (homograft) | Xenograft | 0.468(0.099, 0.838),p=0.013 | -0.156(-0.926, 0.613), p=0.691 | 1.313(0.851, 1.774), p<0.001 |
|  | Mixed | 0.274(-0.110, 0.658), p=0.719 | 0.162 (-0.581, 0.905), p= 0.668 | 0.457(-0.007, 0.921), p=0.053 |

^NYHA: New York heart association; Ross: Ross procedure; RVOT: right ventricular outflow tract. *Studies exclusively about Ross procedure were eliminated.^

Supplementary Table 13: Outcomes after Sensitivity analyses in which studies with sample size below 25^th^ quantile were excluded

| **Outcomes** | | | **Pooled estimate**  **(95%CI)** | | **Heterogeneity**  **(I^2^)** | | **N studies reported** | |
| --- | --- | --- | --- | --- | --- | --- | --- | --- |
|  |  |  | **Before (216)** | **After (162)** | **Before** | **After** | **Before** | **After** |
| ***Early outcomes (%)*** | | | | | | | | |
| **Early mortality** | **All cause** | | 3.36(2.91-3.88) | 3.06(2.60-3.60) | 74.83% | 78.74% | 190 | 140 |
|  | **Cardiac** | | 2.30(1.92-2.76) | 2.02(1.63-2.51) | 59.90% | 67.67% | 152 | 106 |
|  | **Valve-related** | | 0.74(0.60-0.91) | 0.55(0.43-0.70) | 0.00% | 0.00% | 144 | 100 |
|  | **SUD** | | 0.69(0.56-0.86) | 0.50(0.39-0.65) | 0.00% | 0.00% | 142 | 98 |
| **Early PMI/ICD** | | | 2.53(2.00-3.20) | 1.90(1.48-2.43) | 51.91% | 42.93% | 57 | 37 |
| **Re-exploration for bleeding** | | | 5.70(4.79-6.78) | 5.01(4.13-6.07) | 60.17% | 63.09% | 53 | 35 |
| **Early stroke** | | | 1.22(0.93-1.60) | 1.09(0.81-1.46) | 0.00% | 0.00% | 32 | 23 |
| **Early TE/VT** | | | 1.00(0.57-1.74) | 0.70(0.43-1.13) | 45.28% | 0.00% | 27 | 20 |
| **Early MI** | | | 1.25(0.81-1.92) | 0.88(0.51-1.53) | 41.98% | 55.88% | 26 | 17 |
| **Early AKI** | | | 3.28(2.25-4.78) | 2.64(1.71-4.09) | 49.15% | 48.88% | 22 | 12 |
| ***Late outcomes (%/y)*** | | | | | | | | |
| **Late mortality** | | **All cause** | 0.71(0.62-0.82) | 0.65(0.55-0.76) | 79.11% | 83.54% | 189 | 139 |
|  |  | **Cardiac** | 0.43(0.37-0.50) | 0.38(0.32-0.45) | 38.48% | 52.22% | 150 | 105 |
|  |  | **Valve-related** | 0.25(0.21-0.29) | 0.22(0.19-0.26) | 0.00% | 0.00% | 136 | 93 |
|  |  | **SUD** | 0.20(0.17-0.23) | 0.16(0.13-0.19) | 0.00% | 0.00% | 141 | 94 |
| **Late PMI/ICD** | | | 0.43(0.24-0.78) | 0.22(0.13-0.38) | 73.13% | 42.14% | 22 | 13 |
| **Late stroke** | | | 0.18(0.12-0.26) | 0.17(0.12-0.25) | 34.02% | 36.03% | 23 | 21 |
| **Late TE/VT** | | | 0.29(0.21-0.42) | 0.26(0.17-0.38) | 62.82% | 65.12% | 45 | 36 |
| **Late MI** | | | 0.07(0.04-0.14) | 0.07(0.04-0.12) | 11.30% | 0.00% | 11 | 10 |
| ***Overall*** | | | | | | | | |
| **Reintervention** | | | 2.63(2.30-3.02) | 2.41(2.07-2.80) | 95.80% | 96.49% | 209 | 156 |
| **Reoperation** | | | 1.69(1.45-1.97) | 1.59(1.34-1.89) | 93.65% | 95.00% | 178 | 131 |
| **Dysfunction** | | | 3.05(2.25-4.14) | 2.73(1.92, 3.89) | 95.12% | 96.32% | 45 | 30 |
| **Endocarditis** | | | 0.38(0.31-0.47) | 0.34(0.27, 0.43) | 75.91% | 78.60% | 111 | 92 |
| **Moderate to severe PS** | | | 1.89(1.42-2.52) | 1.69(1.20, 2.38) | 90.79% | 93.28% | 59 | 37 |
| **Moderate to severe PR** | | | 2.02(1.60-2.54) | 1.92(1.47, 2.50) | 94.16% | 95.60% | 99 | 72 |

Data expressed as percentage(95%CI). CI: confidential interval; SUD: sudden unexplained death; PMI: permanent pacemaker implantation; ICD: implantable cardioverter defibrillator; TE: thromboembolism event; VT: valve thrombosis; MI: myocardial infarction; AKI: acute kidney injury; PS: pulmonary valve stenosis; PR: pulmonary valve regurgitation.

Supplementary Table 14: Leave-one-out sensitivity analyses on outcomes with “major changes”*

| Outcomes | Pooled estimate  (95%CI) | | Heterogeneity  (I2) | | N studies reported | |
| --- | --- | --- | --- | --- | --- | --- |
|  | Before | After | Before | After | Before | After |
| Early valve related mortality | 0.74(0.60-0.91) | 0.78(0.63-0.96)[123] | 0.00% | 0.00% | 144 | 143 |
| Early SUD | 0.69(0.56-0.86) | 0.66(0.53-0.83)[72] | 0.00% | 0.00% | 142 | 141 |
| Early PMI/ICD | 2.53(2.00-3.20) | 2.64(2.10-3.32) [215] | 51.91% | 48.22% | 57 | 56 |
| Early TE/VT | 1.00(0.57-1.74) | 0.84(0.55-1.30)[87] | 45.28% | 0.00% | 27 | 26 |
| Early MI | 1.25(0.81-1.92) | 1.14(0.77-1.68)[184] | 41.98% | 20.21% | 26 | 25 |
| Late PMI/ICD | 0.43(0.24-0.78) | 0.50(0.29-0.88) [123] | 73.13% | 69.20% | 22 | 21 |

SUD: sudden unexplained death; PMI: permanent pacemaker implantation; ICD: implantable cardioverter defibrillator; TE: thromboembolism event; VT: valve thrombosis; MI: myocardial infarction; AKI: acute kidney injury. All references were the excluded study in leave-one-out sensitivity analyses for each outcome. *By excluding the studies which have the biggest influence on pooled estimates. The number in [] stands for the reference that has been excluded in leave-one-out sensitivity analysis.

Supplementary Table 15: geographical distribution of included articles about Ross procedures and right-sided conduit RVOT

|  | Ross procedure  (n=14,690) | Right-sided conduit RVOT  (n=13,859) |
| --- | --- | --- |
| **Europe** | 10,995 | 4767 |
| **Asia** | 572 | 2174 |
| **New Zealand, Australia** | 341 | 922 |
| **North America** | 2,259 | 5556 |
| **Latin America** | 523 | NA |
| **Unclear** | NA | 440 |

Supplementary Figure 1: Pooled Kaplan-Meier curves of freedom from death in different subgroups. A: accumulative survival probability in subgroups of PVR with homograft and PVR with xenograft; B: accumulative survival probability in subgroups of children and infants; C: accumulative survival probability in subgroups of Ross PVR and right-sided conduit PVR; D: accumulative survival probability in subgroups of patients diagnosed with TA and TOF. TA: truncus arteriosus; TOF: Tetralogy of Fallot.


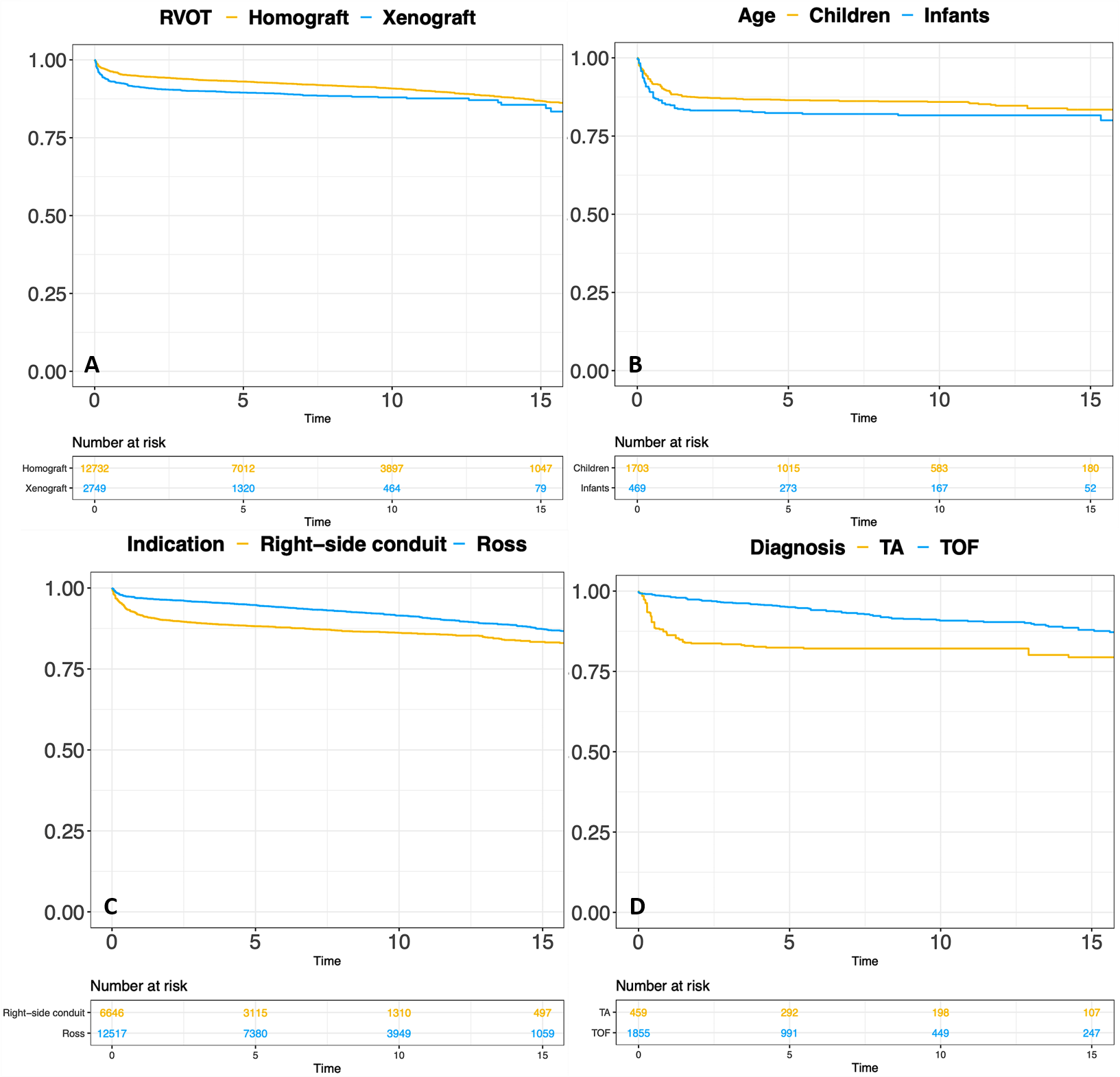


D

C

**References:**

1. Akins CW, Miller DC, Turina MI, Kouchoukos NT, Blackstone EH, Grunkemeier GL, Takkenberg JJ, David TE, Butchart EG, Adams DH *et al*: **Guidelines for reporting mortality and morbidity after cardiac valve interventions**. *Ann Thorac Surg* 2008, **85**(4):1490-1495.

2. Caldarone CA, McCrindle BW, Van Arsdell GS, Coles JG, Webb G, Freedom RM, Williams WG: **Independent factors associated with longevity of prosthetic pulmonary valves and valved conduits**. *J Thorac Cardiovasc Surg* 2000, **120**(6):1022-1031.

3. Sirvydis V, Sudikiene R, Lebetkevicius V: **Ross operation--immediate and mid-term results**. *Cardiovasc Surg* 2000, **8**(7):555-560.

4. Linden PA, Cohn LH: **Medium-term follow up of pulmonary autograft aortic valve replacement: Technical advances and echocardiographic follow up**. *J Heart Valve Dis* 2001, **10**(1):35-42.

5. Corno AF, Hurni M, Griffin H, Galal OM, Payot M, Sekarski N, Tozzi P, von Segesser LK: **Bovine jugular vein as right ventricle-to-pulmonary artery valved conduit**. *J Heart Valve Dis* 2002, **11**(2):242-248.

6. Koul B, Lindholm CJ, Koul M, Roijer A: **Ross operation for bicuspid aortic valve disease in adults: Is it a valid surgical option?** *Scand Cardiovasc J* 2002, **36**(1):48-52.

7. Sakaguchi H, Elkins RC, Lane MM, McCue C: **Effect of prior aortic valve intervention on results of the Ross operation**. *J Heart Valve Dis* 2003, **12**(4):423-429.

8. Warner KG, O'Brien PKH, Rhodes J, Kaur A, Robinson DA, Payne DD: **Expanding the indications for pulmonary valve replacement after repair of tetralogy of Fallot**. *Ann Thorac Surg* 2003, **76**(4):1066-1071.

9. Alphonso N, Baghai M, Dhital K, Mood G, Tulloh R, Austin C, Anderson D: **Midterm results of the Ross procedure**. *Eur J Cardiothorac Surg* 2004, **25**(6):925-930.

10. Hraska V, Krajci M, Haun C, Ntalakoura K, Razek V, Lacour-Gayet F, Weil J, Reichenspurner H: **Ross and Ross-Konno procedure in children and adolescents: mid-term results**. *Eur J Cardiothorac Surg* 2004, **25**(5):742-747.

11. Oosterhof T, Meijboom FJ, Vliegen HW, Hazekamp MG, Zwinderman AH, Bouma BJ, Van Dijk APJ, Mulder BJM: **Long-term follow-up of homograft function after pulmonary valve replacement in patients with tetralogy of Fallot**. *Eur Heart J* 2006, **27**(12):1478-1484.

12. Kwak JG, Lee JR, Kim WH, Kim YJ: **Mid-term Results of the Hancock II Valve and Carpentier-Edward Perimount Valve in the Pulmonary Portion in Congenital Heart Disease**. *Heart Lung Circul* 2010, **19**(4):243-246.

13. Lindsey CW, Parks WJ, Kogon BE, Sallee Iii D, Mahle WT: **Pulmonary Valve Replacement After Tetralogy of Fallot Repair in Preadolescent Patients**. *Ann Thorac Surg* 2010, **89**(1):147-151.

14. Shinkawa T, Anagnostopoulos PV, Johnson NC, Watanabe N, Sapru A, Azakie A: **Performance of bovine pericardial valves in the pulmonary position**. *Ann Thorac Surg* 2010, **90**(4):1295-1300.

15. Jain A, Oster M, Kilgo P, Grudziak J, Jokhadar M, Book W, Kogon BE: **Risk factors associated with morbidity and mortality after pulmonary valve replacement in adult patients with previously corrected tetralogy of fallot**. *Pediatr Cardiol* 2012, **33**(4):601-606.

16. Jang W, Kim YJ, Choi K, Lim HG, Kim WH, Lee JR: **Mid-term results of bioprosthetic pulmonary valve replacement in pulmonary regurgitation after tetralogy of fallot repair**. *Eur J Cardio-thorac Surg* 2012, **42**(1):e1-e8.

17. Vohra HA, Whistance RN, Baliulis G, Janusauskas V, Kaarne M, Veldtman GR, Roman K, Vettukattil JJ, Gnanapragasam J, Salmon AP *et al*: **Midterm evaluation of biological prosthetic valves in the pulmonary position of grown-up patients**. *Thorac Cardiovasc Surg* 2012, **60**(3):205-209.

18. Babu-Narayan SV, Diller GP, Gheta RR, Bastin AJ, Karonis T, Li W, Pennell DJ, Uemura H, Sethia B, Gatzoulis MA *et al*: **Clinical outcomes of surgical pulmonary valve replacement after repair of tetralogy of fallot and potential prognostic value of preoperative cardiopulmonary exercise testing**. *Circulation* 2014, **129**(1):18-27.

19. Rotes AS, Eidem BW, Connolly HM, Bonnichsen CR, Rosedahl JK, Schaff HV, Dearani JA, Burkhart HM: **Long-term follow-up after pulmonary valve replacement in repaired tetralogy of fallot**. *Am J Cardiol* 2014, **114**(6):901-908.

20. Bokma JP, Winter MM, Oosterhof T, Vliegen HW, Van Dijk AP, Hazekamp MG, Koolbergen DR, Groenink M, Mulder BJM, Bouma BJ: **Individualised prediction of pulmonary homograft durability in tetralogy of Fallot**. *Heart* 2015, **101**(21):1717-1723.

21. Escarain MC, Giunta G, Favaloro RR: **Ross procedure in adults: is reoperation a real concern?** *J Heart Valve Dis* 2015, **24**(2):247-252.

22. Oliver JM, Garcia-Hamilton D, Gonzalez AE, Ruiz-Cantador J, Sanchez-Recalde A, Polo ML, Aroca A: **Risk factors for prosthetic pulmonary valve failure in patients with congenital heart disease**. *Am J Cardiol* 2015, **116**(8):1252-1256.

23. Dunne B, Suthers E, Xiao P, Xiao J, Litton E, Andrews D: **Medium-term outcomes after pulmonary valve replacement with the freestyle valve for congenital heart disease: A case series**. *Eur J Cardio-thorac Surg* 2016, **49**(5):e105-e111.

24. Kwak JG, Lee C, Lee M, Lee CH, Jang SI, Lee SY, Park SJ, Song MK, Kim SH: **Does implantation of larger bioprosthetic pulmonary valves in young patients guarantee durability in adults? Durability analysis of stented bioprosthetic valves in the pulmonary position in patients with Tetralogy of Fallot**. *Eur J Cardio-thorac Surg* 2016, **49**(4):1207-1212.

25. Haas NA, Bach S, Vcasna R, Laser KT, Sandica E, Blanz U, Jakob A, Dietl M, Fischer M, Kanaan M *et al*: **The risk of bacterial endocarditis after percutaneous and surgical biological pulmonary valve implantation**. *Int J Cardiol* 2018, **268**:55-60.

26. Li WF, Pollard H, Karimi M, Asnes JD, Hellenbrand WE, Shabanova V, Weismann CG: **Comparison of valvar and right ventricular function following transcatheter and surgical pulmonary valve replacement**. *Congenit heart dis* 2018, **13**(1):140-146.

27. Lluri G, Levi DS, Miller E, Hageman A, Sinha S, Sadeghi S, Reemtsen B, Laks H, Biniwale R, Salem M *et al*: **Incidence and outcome of infective endocarditis following percutaneous versus surgical pulmonary valve replacement**. *Catheter Cardiovasc Interventions* 2018, **91**(2):277-284.

28. He F, Feng Z, Chen Q, Jiao Y, Hua Z, Zhang H, Yang K, Pang K, Lu M, Ma K *et al*: **Whether pulmonary valve replacement in asymptomatic patients with moderate or severe regurgitation after tetralogy of fallot repair is appropriate: A case-control study**. *J Am Heart Assoc* 2019, **8**(1).

29. Marathe SP, Bell D, Betts K, Sayed S, Dunne B, Ward C, Whight C, Jalali H, Venugopal P, Andrews D *et al*: **Homografts versus stentless bioprosthetic valves in the pulmonary position: A multicentre propensity-matched comparison in patients younger than 20 years**. *Eur J Cardio-thorac Surg* 2019, **56**(2):377-384.

30. Wijayarathne PM, Skillington P, Menahem S, Thuraisingam A, Larobina M, Grigg L: **Pulmonary Allograft Versus Medtronic Freestyle Valve in Surgical Pulmonary Valve Replacement for Adults Following Correction of Tetralogy of Fallot or Its Variants**. *World J Pediatr Congenit Heart Surg* 2019, **10**(5):543-551.

31. Bove T, Bradt N, Martens T, De Wolf D, François K, de Beco G, Sluysmans T, Rubay J, Poncelet A: **The pulmonary autograft after the Ross operation: Results of 25 year follow-up in a pediatric cohort**. *Ann Thorac Surg* 2020.

32. Feins EN, Chavez M, Callahan R, Del Nido PJ, Emani SM, Baird CW: **Experience and Outcomes of Surgically Implanted Melody Valve in the Pulmonary Position**. 2020.

33. Lee C, Choi ES, Lee CH: **Long-term outcomes of pulmonary valve replacement in patients with repaired tetralogy of Fallot**. *Eur J Cardiothorac Surg* 2020, **58**(2):246-252.

34. Patel PM, Herrmann JL, Rodefeld MD, Turrentine MW, Brown JW: **Bovine jugular vein conduit versus pulmonary homograft in the Ross operation**. *Cardiol Young* 2020, **30**(3):323-327.

35. Pragt H, Schoots MH, Accord RE, Arrigoni SC, Berger RM, Mariani MA, Willems TP, Ebels T, Van Melle JP: **A stented bovine pericardial prosthesis in the pulmonary position**. *J Thorac Cardiovasc Surg* 2020, **159**(3):1063-1071.e1061.

36. Benjacholamas V, Namchaisiri J, Khongphatthanayothin A, Lertsapcharoen P: **Bicuspidized pulmonary homograft for truncus arteriosus repair**. *Asian Cardiovasc Thorac Ann* 2008, **16**(3):189-193.

37. Fiore AC, Rodefeld M, Turrentine M, Vijay P, Reynolds T, Standeven J, Hill K, Bost J, Carpenter D, Tobin C *et al*: **Pulmonary Valve Replacement: A Comparison of Three Biological Valves**. *Ann Thorac Surg* 2008, **85**(5):1712-1718.

38. Tokunaga S, Masuda M, Shiose A, Tomita Y, Morita S, Tominaga R: **Isolated pulmonary valve replacement: analysis of 27 years of experience**. *Journal of Artificial Organs* 2008, **11**(3):130-133.

39. Dave H, Mueggler O, Comber M, Enodien B, Nikolaou G, Bauersfeld U, Jenni R, Bettex D, Prêtre R: **Risk factor analysis of 170 single-institutional contegra implantations in pulmonary position**. *Ann Thorac Surg* 2011, **91**(1):195-203.

40. Chen XJ, Smith PB, Jaggers J, Lodge AJ: **Bioprosthetic pulmonary valve replacement: Contemporary analysis of a large, single-center series of 170 cases**. *J Thorac Cardiovasc Surg* 2013, **146**(6):1461-1466.

41. Voges I, Bräsen JH, Entenmann A, Scheid M, Scheewe J, Fischer G, Hart C, Andrade A, Pham HM, Kramer HH *et al*: **Adverse results of a decellularized tissue-engineered pulmonary valve in humans assessed with magnetic resonance imaging**. *Eur J Cardio-thorac Surg* 2013, **44**(4):e272-e279.

42. Ramanan S, Doll N, Boethig D, Tafer N, Horke A, Roques X, Hemmer WB, Roubertie F: **Pulmonary-valve replacement in adults: Results with the medtronic freestyle valve**. *Ann Thorac Surg* 2015, **100**(3):1047-1053.

43. Shinkawa T, Lu CK, Chipman C, Tang X, Gossett JM, Imamura M: **The Midterm Outcomes of Bioprosthetic Pulmonary Valve Replacement in Children**. *Semin Thorac Cardiovasc Surg* 2015, **27**(3):310-318.

44. Mitropoulos FA, Kanakis MA, Ntellos C, Loukas C, Davlouros P, Kousi T, Chatzis AC: **Pulmonary valve replacement in patients with corrected tetralogy of Fallot**. *J Cardiovasc Thorac Res* 2017, **9**(2):71-77.

45. Seilani P, Nasiri-Brojeni M, Saedi S, Maleki M, Saedi T: **Midterm longevity of bioprosthetic pulmonary valves in congenital heart disease patients**. *Iran Heart J* 2017, **18**(3):21-27.

46. Bell D, Prabhu S, Betts KS, Chen Y, Radford D, Whight C, Ward C, Jalali H, Venugopal P, Alphonso N: **Long-term performance of homografts versus stented bioprosthetic valves in the pulmonary position in patients aged 10–20 years†**. *Eur J Cardio-thorac Surg* 2018, **54**(5):946-952.

47. Robichaud B, Hill G, Cohen S, Woods R, Earing M, Frommelt P, Ginde S: **Bioprosthetic pulmonary valve endocarditis: Incidence, risk factors, and clinical outcomes**. *Congenit Heart Dis* 2018, **13**(5):734-739.

48. Cocomello L, Meloni M, Rapetto F, Baquedano M, Ordoñez MV, Biglino G, Bucciarelli-Ducci C, Parry A, Stoica S, Caputo M: **Long-Term Comparison Between Pulmonary Homograft Versus Bioprosthesis for Pulmonary Valve Replacement in Tetralogy of Fallot**. *J Am Heart Assoc* 2019, **8**(24):e013654.

49. Nichay NR, Zhuravleva IY, Kulyabin YY, Zubritskiy AV, Voitov AV, Soynov IA, Gorbatykh AV, Bogachev-Prokophiev AV, Karaskov AM: **Diepoxy- Versus Glutaraldehyde-Treated Xenografts: Outcomes of Right Ventricular Outflow Tract Reconstruction in Children**. *World J Pediatr Congenit Heart Surg* 2020, **11**(1):56-64.

50. Spigel ZA, Zhu H, Qureshi AM, Penny DJ, Caldarone CA, Heinle JS, Binsalamah ZM: **Durability of the St. Jude Epic Supra Bioprosthetic Valve in the Pulmonary Position**. *Semin Thorac Cardiovasc Surg* 2020.

51. Wasilewski G, Suder B, Kędziora A, Litwinowicz R, Mazur P, Tomkiewicz-Pająk L, Kapelak B: **Outcomes of tetralogy of Fallot reoperation in adults: a single-center experience with bioprosthetic pulmonary valve replacement**. *Kardiol Pol* 2020.

52. Al-Halees Z, Pieters F, Qadoura F, Shahid M, Al-Amri M, Al-Fadley F: **The Ross procedure is the procedure of choice for congenital aortic valve disease**. *J Thorac Cardiovasc Surg* 2002, **123**(3):437-441; discussion 441-432.

53. Sievers H, Dahmen G, Graf B, Stierle U, Ziegler A, Schmidtke C: **Midterm results of the Ross procedure preserving the patient's aortic root**. *Circulation* 2003, **108 Suppl 1**:II55-60.

54. Matalanis G, Durairaj M, Shah P, Buxton B: **Early and midterm results with the Ross procedure: A study of the first 31 cases**. *Asian Cardiovasc Thorac Ann* 2004, **12**(4):336-340.

55. Raja SG, Pozzi M: **Ross operation in children and young adults: The Alder Hey case series**. *BMC Cardiovasc Disord* 2004, **4**.

56. Williams IA, Quaegebeur JM, Hsu DT, Gersony WM, Bourlon F, Mosca RS, Gersony DR, Solowiejczyk DE: **Ross procedure in infants and toddlers followed into childhood**. *Circulation* 2005, **112**(9 Suppl):I390-395.

57. Frigiola A, Ranucci M, Carlucci C, Giamberti A, Abella R, Di Donato M: **The Ross Procedure in Adults: Long-Term Follow-Up and Echocardiographic Changes Leading to Pulmonary Autograft Reoperation**. *Ann Thorac Surg* 2008, **86**(2):482-489.

58. Morales DL, Carberry KE, Balentine C, Heinle JS, McKenzie ED, Fraser CD, Jr.: **Selective application of the pediatric Ross procedure minimizes autograft failure**. *Congenit heart dis* 2008, **3**(6):404-410.

59. Brown JW, Fehrenbacher JW, Ruzmetov M, Shahriari A, Miller J, Turrentine MW: **Ross root dilation in adult patients: is preoperative aortic insufficiency associated with increased late autograft reoperation?** *Ann Thorac Surg* 2011, **92**(1):74-81; discussion 81.

60. Ryan WH, Prince SL, Culica D, Herbert MA: **The Ross procedure performed for aortic insufficiency is associated with increased autograft reoperation**. *Ann Thorac Surg* 2011, **91**(1):64-69; discussion 69-70.

61. Xu Z, Li W, Xu X, Zhou Z, Song S, Ma J, Zhang J: **Long-term follow-up with Ross procedure at a single institution in China**. *Thorac Cardiovasc Surg* 2014, **62**(3):216-221.

62. Christ T, Claus B, Woythal N, Dushe S, Falk V, Grubitzsch H: **The Ross Procedure in Adults: Long-Term Results of Homografts and Stentless Xenografts for Pulmonary Valve Replacement**. *Thorac Cardiovasc Surg* 2017, **65**(8):656-661.

63. Martin E, Mohammadi S, Jacques F, Kalavrouziotis D, Voisine P, Doyle D, Perron J: **Clinical Outcomes Following the Ross Procedure in Adults: A 25-Year Longitudinal Study**. *J Am Coll Cardiol* 2017, **70**(15):1890-1899.

64. Pardo González L, Ruiz Ortiz M, Delgado M, Mesa D, Villalba R, Rodriguez S, Hidalgo FJ, Alados P, Casares J, Suarez de Lezo J: **Pulmonary homograft stenosis in the Ross procedure: Incidence, clinical impact and predictors in long-term follow-up**. *Arch Cardiovasc Dis* 2017, **110**(4):214-222.

65. Alassas K, Mohty D, Clavel MA, Husain A, Hijji T, Aljoufan M, Alhalees Z, Fadel BM: **Transcatheter versus surgical valve replacement for a failed pulmonary homograft in the Ross population**. *J Thorac Cardiovasc Surg* 2018, **155**(4):1434-1444.

66. David TE, Ouzounian M, David CM, Lafreniere-Roula M, Manlhiot C: **Late results of the Ross procedure**. *J Thorac Cardiovasc Surg* 2019, **157**(1):201-208.

67. Guerreiro S, Madeira M, Ribeiras R, Queiroz EMJ, Canada M, Horta E, Reis C, Neves JP, Mendes M: **Long-term assessment of the Ross procedure in adults: Clinical and echocardiographic follow-up at 20 years**. *Rev Port Cardiol* 2019, **38**(5):315-321.

68. Riggs KW, Colohan DB, Beacher DR, Alsaied T, Powell S, Moore RA, Ginde S, Tweddell JS: **Mid-term Outcomes of the Supported Ross Procedure in Children, Teenagers, and Young Adults**. *Semin Thorac Cardiovasc Surg* 2020, **32**(3):498-504.

69. Sharifulin R, Bogachev-Prokophiev A, Demin I, Afanasyev A, Ovcharov M, Pivkin A, Sapegin A, Zhuravleva I, Karaskov A: **Allografts and xenografts for right ventricular outflow tract reconstruction in Ross patients**. *Eur J Cardiothorac Surg* 2020.

70. Skillington PD, Mokhles MM, Takkenberg JJM, O'Keefe M, Grigg L, Wilson W, Larobina M, Tatoulis J: **Twenty-year analysis of autologous support of the pulmonary autograft in the ross procedure**. *Ann Thorac Surg* 2013, **96**(3):823-829.

71. Salehi M, Sattarzadeh R, Soleimani AA, Radmehr H, Mirhosseini J, Sanatkar Far M: **The Ross operation: Clinical results and echocardiographic findings**. *Asian Cardiovasc Thorac Ann* 2007, **15**(1):30-34.

72. Juthier F, Vincentelli A, Pinon C, Banfi C, Ennezat PV, Marchaux S, Prat A: **Reoperation after the ross procedure: Incidence, management, and survival**. *Ann Thorac Surg* 2012, **93**(2):598-605.

73. Mastrobuoni S, de Kerchove L, Solari S, Astarci P, Poncelet A, Noirhomme P, Rubay J, El Khoury G: **The Ross procedure in young adults: over 20 years of experience in our Institution**. *Eur J Cardiothorac Surg* 2016, **49**(2):507-512; discussion 512-503.

74. Miskovic A, Monsefi N, Karimian-Tabrizi A, Zierer A, Moritz A: **A 17-year, single-centre experience with the Ross procedure: fulfilling the promise of a durable option without anticoagulation?** *Eur J Cardiothorac Surg* 2016, **49**(2):514-519; discussion 519.

75. Schneider AW, Putter H, Klautz RJM, Bruggemans EF, Holman ER, Bokenkamp R, Hazekamp MG: **Long-Term Follow-Up After the Ross Procedure: A Single Center 22-Year Experience**. *Ann Thorac Surg* 2017, **103**(6):1976-1983.

76. Van Dijck I, Budts W, Cools B, Eyskens B, Boshoff DE, Heying R, Frerich S, Vanagt WY, Troost E, Gewillig M: **Infective endocarditis of a transcatheter pulmonary valve in comparison with surgical implants**. *Heart* 2015, **101**(10):788-793.

77. Urso S, Rega F, Meuris B, Gewillig M, Eyskens B, Daenen W, Heying R, Meyns B: **The Contegra conduit in the right ventricular outflow tract is an independent risk factor for graft replacement**. *Eur J Cardio-thorac Surg* 2011, **40**(3):603-609.

78. Ugaki S, Rutledge J, Al Aklabi M, Ross DB, Adatia I, Rebeyka IM: **An Increased Incidence of Conduit Endocarditis in Patients Receiving Bovine Jugular Vein Grafts Compared to Cryopreserved Homograft for Right Ventricular Outflow Reconstruction**. *Ann Thorac Surg* 2015, **99**(1):140-147.

79. Tweddell JS, Pelech AN, Frommelt PC, Mussatto KA, Wyman JD, Fedderly RT, Berger S, Frommelt MA, Lewis DA, Friedberg DZ *et al*: **Factors affecting longevity of homograft valves used in right ventricular outflow tract reconstruction for congenital heart disease**. *Circulation* 2000, **102**(19):III130-135.

80. Tierney ESS, Gersony WM, Altmann K, Solowiejczyk DE, Bevilacqua LM, Khan C, Krongrad E, Mosca RS, Quaegebeur JM, Apfel HD: **Pulmonary position cryopreserved homografts: Durability in pediatric Ross and non-Ross patients**. *J Thorac Cardiovasc Surg* 2005, **130**(2):282-286.

81. Thompson LD, McElhinney DB, Reddy VM, Petrossian E, Silverman NH, Hanley FL: **Neonatal repair of truncus arteriosus: Continuing improvement in outcomes**. *Ann Thorac Surg* 2001, **72**(2):391-395.

82. Sinzobahamvya N, Boscheinen M, Blaschczok HC, Kallenberg R, Photiadis J, Haun C, Hraska V, Asfour B: **Survival and reintervention after neonatal repair of truncus arteriosus with valved conduit**. *Eur J Cardio-thorac Surg* 2008, **34**(4):732-737.

83. Sinzobahamvya N, Asfour B, Boscheinen M, Photiadis J, Fink C, Schindler E, Hraska V, Brecher AM: **Compared fate of small-diameter Contegras® and homografts in the pulmonary position**. *Eur J Cardio-thorac Surg* 2007, **32**(2):209-214.

84. Shih T, Gurney JG, Bove EL, Devaney EJ, Hirsch JC, Ohye RG: **Performance of bicuspidized pulmonary allografts compared with standard trileaflet allografts**. *Ann Thorac Surg* 2010, **90**(2):610-612.

85. Shebani SO, McGuirk S, Baghai M, Stickley J, De Giovanni JV, Bu'Lock FA, Barron DJ, Brawn WJ: **Right ventricular outflow tract reconstruction using Contegra® valved conduit: Natural history and conduit performance under pressure**. *Eur J Cardio-thorac Surg* 2006, **29**(3):397-405.

86. Sharma V, Griffiths ER, Eckhauser AW, Gray RG, Martin MH, Zhang C, Presson AP, Burch PT: **Pulmonary Valve Replacement: A Single-Institution Comparison of Surgical and Transcatheter Valves**. *Ann Thorac Surg* 2018, **106**(3):807-813.

87. Sfyridis PG, Avramidis DP, Kirvassilis GV, Zavaropoulos PN, Papagiannis JK, Sarris GE: **The contegra® valved heterograft conduit for right ventricular outflow tract reconstruction: A reliable solution**. *Hell J Cardiol* 2011, **52**(6):501-508.

88. Sekarski N, van Meir H, Rijlaarsdam MEB, Schoof PH, Koolbergen DR, Hruda J, von Segesser LK, Meijboom EJ, Hazekamp MG: **Right Ventricular Outflow Tract Reconstruction With the Bovine Jugular Vein Graft: 5 Years' Experience With 133 Patients**. *Ann Thorac Surg* 2007, **84**(2):599-605.

89. Schubmehl HB, Swartz MF, Atallah-Yunes N, Wittlieb-Weber C, Pratt RE, Alfieris GM: **Sustained Improvement in Right Ventricular Chamber Dimensions 10 Years Following Xenograft Pulmonary Valve Replacement**. *World J Pediatr Congenit Heart Surg* 2017, **8**(1):39-47.

90. Schoenhoff FS, Loup O, Gahl B, Banz Y, Pavlovic M, Pfammatter JP, Carrel TP, Kadner A: **The Contegra bovine jugular vein graft versus the Shelhigh pulmonic porcine graft for reconstruction of the right ventricular outflow tract: A comparative study**. *J Thorac Cardiovasc Surg* 2011, **141**(3):654-661.

91. Bielefeld MR, Bishop DA, Campbell DN, Mitchell MB, Grover FL, Clarke DR: **Reoperative homogtaft right ventricular outflow tract reconstruction**. *Ann Thorac Surg* 2001, **71**(2):482-488.

92. Schiralli MP, Cholette JM, Swartz MF, Vermilion R, Meagher C, Alfieris GM: **Carpentier Edwards porcine valved conduit for right ventricular outflow tract reconstruction**. *J Card Surg* 2011, **26**(6):643-649.

93. Saritas B, Ozker E, Ozkan M, Sarisoy O, Ayabakan C: **Early surgical outcome of pulmonary valve replacement in patients with previous right ventricle outflow tract reconstruction**. *Turk Gogus Kalp Damar Cerrahisi Dergisi-Turkish Journal of Thoracic and Cardiovascular Surgery* 2017, **25**(3):363-367.

94. Sarikouch S, Horke A, Tudorache I, Beerbaum P, Westhoff-Bleck M, Boethig D, Repin O, Maniuc L, Ciubotaru A, Haverich A *et al*: **Decellularized fresh homografts for pulmonary valve replacement: A decade of clinical experience**. *Eur J Cardio-thorac Surg* 2016, **50**(2):281-290.

95. Ruzmetov M, Shah JJ, Geiss DM, Fortuna RS: **Decellularized versus standard cryopreserved valve allografts for right ventricular outflow tract reconstruction: a single-institution comparison**. *J Thorac Cardiovasc Surg* 2012, **143**(3):543-549.

96. Ruzmetov M, Geiss DM, Fortuna RS: **Outcomes of pericardial bovine xenografts for right ventricular outflow tract reconstruction in children and young adults**. *J Heart Valve Dis* 2013, **22**(2):209-214.

97. Rastan AJ, Walther T, Daehnert I, Hambsch J, Mohr FW, Janousek J, Kostelka M: **Bovine Jugular Vein Conduit for Right Ventricular Outflow Tract Reconstruction: Evaluation of Risk Factors for Mid-Term Outcome**. *Ann Thorac Surg* 2006, **82**(4):1308-1315.

98. Prior N, Alphonso N, Arnold P, Peart I, Thorburn K, Venugopal P, Corno AF: **Bovine jugular vein valved conduit: Up to 10 years follow-up**. *J Thorac Cardiovasc Surg* 2011, **141**(4):983-987.

99. Pragt H, van Melle JP, Javadikasgari H, Seo DM, Stulak JM, Knez I, Hörer J, Muñoz-Guijosa C, Dehaki MG, Shin HJ *et al*: **Mechanical valves in the pulmonary position: An international retrospective analysis**. *J Thorac Cardiovasc Surg* 2017, **154**(4):1371-1378 e1371.

100. Perri G, Polito A, Esposito C, Albanese SB, Francalanci P, Pongiglione G, Carotti A: **Early and late failure of tissue-engineered pulmonary valve conduits used for right ventricular outflow tract reconstruction in patients with congenital heart disease**. *Eur J Cardio-thorac Surg* 2012, **41**(6):1320-1325.

101. Bibevski S, Ruzmetov M, Fortuna RS, Turrentine MW, Brown JW, Ohye RG: **Performance of SynerGraft Decellularized Pulmonary Allografts Compared With Standard Cryopreserved Allografts: Results From Multiinstitutional Data**. *Ann Thorac Surg* 2017, **103**(3):869-874.

102. Pawelec-Wojtalik M, Mrówczyński W, Wodziński A, Wojtalik M, Henschke J, Sharma GK: **Mid-term experience with valved bovine jugular vein conduits**. *Asian Cardiovasc Thorac Ann* 2005, **13**(4):361-365.

103. Ong K, Boone R, Gao M, Carere R, Webb J: **Right ventricle to pulmonary artery conduit reoperations in patients with tetralogy of fallot or pulmonary atresia associated with ventricular septal defect**: Elsevier; 2013.

104. Nordmeyer J, Tsang V, Gaudin R, Lurz P, Frigiola A, Jones A, Schievano S, Van Doorn C, Bonhoeffer P, Taylor AM: **Quantitative assessment of homograft function 1 year after insertion into the pulmonary position: Impact of in situ homograft geometry on valve competence**. *Eur Heart J* 2009, **30**(17):2147-2154.

105. Niemantsverdriet MBA, Ottenkamp J, Gauvreau K, Del Nido PJ, Hazenkamp MG, Jenkins KJ: **Determinants of right ventricular outflow tract conduit longevity: A multinational analysis**. *Congenit Heart Dis* 2008, **3**(3):176-184.

106. Morales DLS, Braud BE, Gunter KS, Carberry KE, Arrington KA, Heinle JS, McKenzie ED, Fraser Jr CD: **Encouraging results for the Contegra conduit in the problematic right ventricle-to-pulmonary artery connection**. *J Thorac Cardiovasc Surg* 2006, **132**(3):665-671.

107. Morales DLS, Braud BE, Dibardino DJ, Carberry KE, McKenzie DE, Heinle JS, Fraser Jr CD: **Perimount® bovine pericardial valve to restore pulmonary valve competence late after right ventricular outflow tract repair**. *Congenit Heart Dis* 2007, **2**(2):115-120.

108. Mokhles MM, van de Woestijne PC, de Jong PL, Witsenburg M, Roos-Hesselink JW, Takkenberg JJM, Bogers AJJC: **Clinical outcome and health-related quality of life after right-ventricular-outflow-tract reconstruction with an allograft conduit**. *Eur J Cardio-thorac Surg* 2011, **40**(3):571-578.

109. Meyns B, Van Garsse L, Boshoff D, Eyskens B, Mertens L, Gewillig M, Fieuws S, Verbeken E, Daenen W: **The Contegra conduit in the right ventricular outflow tract induces supravalvular stenosis**. *J Thorac Cardiovasc Surg* 2004, **128**(6):834-840.

110. Batlivala SP, Emani S, Mayer JE, McElhinney DB: **Pulmonary valve replacement function in adolescents: A comparison of bioprosthetic valves and homograft conduits**. *Ann Thorac Surg* 2012, **93**(6):2007-2016.

111. Mercer CW, West SC, Sharma MS, Yoshida M, Morell VO: **Polytetrafluoroethylene conduits versus homografts for right ventricular outflow tract reconstruction in infants and young children: An institutional experience**. *J Thorac Cardiovasc Surg* 2018, **155**(5):2082-2091.e2081.

112. Alsoufi B, Awan A, Al-Omrani A, Al-Ahmadi M, Canver CC, Bulbul Z, Kalloghlian A, Al-Halees Z: **The rastelli procedure for transposition of the great arteries: resection of the infundibular septum diminishes recurrent left ventricular outflow tract obstruction risk**. *Ann Thorac Surg* 2009, **88**(1):137-142; discussion 142-133.

113. Lund AM, Vogel M, Marshall AC, Emani SM, Pigula FA, Tworetzky W, McElhinney DB: **Early reintervention on the pulmonary arteries and right ventricular outflow tract after neonatal or early infant repair of truncus arteriosus using homograft conduits**. *Am J Cardiol* 2011, **108**(1):106-113.

114. Louis C, Swartz MF, Simon BV, Cholette JM, Atallah-Yunes N, Wang H, Gensini F, Alfieris GM: **Modified Repair of Type I and II Truncus Arteriosus Limits Early Right Ventricular Outflow Tract Reoperation**. *Semin Thorac Cardiovasc Surg* 2018, **30**(2):199-204.

115. Alsoufi B, Al-Halees Z, Manlhiot C, McCrindle BW, Kandeel M, Al-Joufan M, Kalloghlian A, Fadel B, Canver CC: **Superior results following the Ross procedure in patients with congenital heart disease**. *J Heart Valve Dis* 2010, **19**(3):269-277; discussion 278.

116. Andreas M, Seebacher G, Reida E, Wiedemann D, Pees C, Rosenhek R, Heinze G, Moritz A, Kocher A, Laufer G: **A single-center experience with the Ross procedure over 20 years**. *Ann Thorac Surg* 2014, **97**(1):182-188.

117. Bansal N, Kumar SR, Baker CJ, Lemus R, Wells WJ, Starnes VA: **Age-related outcomes of the Ross procedure over 20 years**. *Ann Thorac Surg* 2015, **99**(6):2077-2085.

118. Bechtel JF, Stierle U, Sievers HH: **Fifty-two months' mean follow up of decellularized SynerGraft-treated pulmonary valve allografts**. *J Heart Valve Dis* 2008, **17**(1):98-104; discussion 104.

119. Boethig D, Goerler H, Westhoff-Bleck M, Ono M, Daiber A, Haverich A, Breymann T: **Evaluation of 188 consecutive homografts implanted in pulmonary position after 20 years**. *Eur J Cardio-thorac Surg* 2007, **32**(1):133-142.

120. Boethlg D, Westhoff-Bleck M, Hecker H, Ono M, Goerler A, Sarikouch S, Breymann T: **Bovine jugular veins in the pulmonary position in adults - 5 years' experience with 64 implantations**. *Thorac Cardiovasc Surg* 2009, **57**(4):196-201.

121. Bramer S, Mokhles MM, Takkenberg JJM, Bogers AJJC: **Long-term outcome of right ventricular outflow tract reconstruction with bicuspidalized homografts**. *Eur J Cardio-thorac Surg* 2011.

122. Breymann T, Blanz U, Wojtalik MA, Daenen W, Hetzer R, Sarris G, Stellin G, Planche C, Tsang V, Weissmann N *et al*: **European Contegra multicentre study: 7-year results after 165 valved bovine jugular vein graft implantations**. *Thorac Cardiovasc Surg* 2009, **57**(5):257-269.

123. Brown JW, Elkins RC, Clarke DR, Tweddell JS, Huddleston CB, Doty JR, Fehrenbacher JW, Takkenberg JJM: **Performance of the CryoValve* SG human decellularized pulmonary valve in 342 patients relative to the conventional CryoValve at a mean follow-up of four years**. *J Thorac Cardiovasc Surg* 2010, **139**(2):339-348.

124. Brown JW, Ruzmetov M, Rodefeld MD, Eltayeb O, Yurdakok O, Turrentine MW: **Contegra versus pulmonary homografts for right ventricular outflow tract reconstruction: a ten-year single-institution comparison**. *World J Pediatr Congenit Heart Surg* 2011, **2**(4):541-549.

125. Brown JW, Ruzmetov M, Rodefeld MD, Vijay P, Turrentine MW: **Right ventricular outflow tract reconstruction with an allograft conduit in non-ross patients: risk factors for allograft dysfunction and failure**. *Ann Thorac Surg* 2005, **80**(2):655-663; discussion 663-654.

126. Burch PT, Kaza AK, Lambert LM, Holubkov R: **Clinical performance of decellularized cryopreserved valved allografts compared with standard allografts in the right ventricular outflow tract**: Elsevier; 2010.

127. Chen H, Shi G, Qiu L, Wang S, Chen H, Xu Z: **Outcomes of Prosthetic Valved Conduits for Right Ventricular Outflow Tract Reconstruction**. *Pediatr Cardiol* 2019.

128. Christenson JT, Sierra J, Colina Manzano NE, Jolou J, Beghetti M, Kalangos A: **Homografts and xenografts for right ventricular outflow tract reconstruction: long-term results**. *Ann Thorac Surg* 2010, **90**(4):1287-1293.

129. Cleuziou J, Vitanova K, Kasnar-Samprec J, Hörer J, Lange R, Schreiber S: **Durability of down-sized homografts for the reconstruction of the right ventricular outflow tract**. *Eur J Cardio-thorac Surg* 2016, **49**(5):1421-1425.

130. Concha M, Pradas G, Juffé A, Caffarena JM, Montero A, Aranda PJ: **Comprehensive experience with the Ross operation in Spain**. *Eur J Cardio-thorac Surg* 2003, **24**(4):521-526.

131. Corno AF, Dawson AG, Bolger AP, Mimic B, Shebani SO, Skinner GJ, Speggiorin S: **Trifecta St. Jude medical aortic valve in pulmonary position**. *Nano Rev Exp* 2017, **8**(1):1299900.

132. Dekens E, Van Damme E, Jashari R, Van Hoeck B, François K, Bové T: **Durability of pulmonary homografts for reconstruction of the right ventricular outflow tract: How relevant are donor-related factors?** *Interact Cardiovasc Thorac Surg* 2019, **28**(4):503-509.

133. Dohmen PM, Lembcke A, Holinski S, Kivelitz D, Braun JP, Pruss A, Konertz W: **Mid-Term Clinical Results Using a Tissue-Engineered Pulmonary Valve to Reconstruct the Right Ventricular Outflow Tract During the Ross Procedure**. *Ann Thorac Surg* 2007, **84**(3):729-736.

134. Elder RW, Quaegebeur JM, Bacha EA, Chen JM, Bourlon F, Williams IA: **Outcomes of the infant Ross procedure for congenital aortic stenosis followed into adolescence**. *J Thorac Cardiovasc Surg* 2013, **145**(6):1504-1511.

135. Etnel JRG, Suss PH, Schnorr GM, Veloso M, Colatusso DF, Filho EMB, Costa FDAD: **Fresh decellularized versus standard cryopreserved pulmonary allografts for right ventricular outflow tract reconstruction during the Ross procedure: A propensity-matched study**. *Eur J Cardio-thorac Surg* 2018, **54**(3):434-440.

136. Falchetti A, Demanet H, Dessy H, Melot C, Pierrakos C, Wauthy P: **Contegra versus pulmonary homograft for right ventricular outflow tract reconstruction in newborns**. *Cardiol Young* 2019, **29**(4):505-510.

137. Fiore AC, Brown JW, Turrentine MW, Ruzmetov M, Huynh D, Hanley S, Rodefeld MD: **A Bovine Jugular Vein Conduit: A Ten-Year Bi-Institutional Experience**. *Ann Thorac Surg* 2011, **92**(1):183-192.

138. Fiore AC, Ruzmetov M, Huynh D, Hanley S, Rodefeld MD, Turrentine MW, Brown JW: **Comparison of bovine jugular vein with pulmonary homograft conduits in children less than 2 years of age**. *Eur J Cardio-thorac Surg* 2010, **38**(3):318-325.

139. Francoisa K, De Groote K, Vandekerckhove K, De Wilde H, De Wolf D, Bove T: **Small-sized conduits in the right ventricular outflow tract in young children: bicuspidalized homografts are a good alternative to standard conduits**. *Eur J Cardio-Thorac Surg* 2018, **53**(2):409-415.

140. Fullerton DA, Fredericksen JW, Sundaresan RS, Horvath KA, Calhoon JH, Drinkwater DC, Oswalt JD: **The Ross procedure in adults: Intermediate-term results**. *Ann Thorac Surg* 2003, **76**(2):471-477.

141. Giamberti A, Chessa M, Reali M, Varrica A, Nuri H, Isgrò G, Frigiola A, Ranucci M: **Porcine bioprosthetic valve in the pulmonary position: Mid-term results in the right ventricular outflow tract reconstruction**. *Pediatr Cardiol* 2013, **34**(5):1190-1193.

142. Gröning M, Tahri NB, Søndergaard L, Helvind M, Ersbøll MK, Ørbæk Andersen H: **Infective endocarditis in right ventricular outflow tract conduits: a register-based comparison of homografts, Contegra grafts and Melody transcatheter valves**. *Eur J Cardio-thorac Surg* 2019, **56**(1):87-93.

143. Holmes A, Co S, Human D, LeBlanc J, Campbell A: **The Contegra conduit: Late outcomes in right ventricular outflow tract reconstruction**. *Ann Pediatr Cardiol* 2012, **5**(1):27-33.

144. Homann M, Haehnel JC, Mendler N, Paek SU, Holper K, Meisner H, Lange R: **Reconstruction of the RVOT with valved biological conduits: 25 years experience with allografts and xenografts**. *Eur J Cardiothorac Surg* 2000, **17**(6):624-630.

145. Hoxha S, Torre S, Rungatscher A, Sandrini C, Rossetti L, Barozzi L, Faggian G, Luciani GB: **Twenty-Year Outcome After Right Ventricular Outflow Tract Repair Using Heterotopic Pulmonary Conduits in Infants and Children**. *Artif Organs* 2016, **40**(1):50-55.

146. Hunter J, Rosenkranz E, Li H, Swaminathan S: **Assessment of the longevity of valves placed in the pulmonary position in patients with congenital heart disease**. *Prog Pediatr Cardiol* 2016, **43**:133-139.

147. Jussli-Melchers J, Scheewe J, Hansen JH, Grothusen C, Steer J, Voges I, Logoteta J, Dütschke P, Kramer HH, Attmann T: **Right ventricular outflow tract reconstruction with the Labcor® stentless valved pulmonary conduit**. *Eur J Cardiothorac Surg* 2019.

148. Kalavrouziotis G, Purohit M, Ciotti G, Corno AF, Pozzi M: **Truncus Arteriosus Communis: Early and Midterm Results of Early Primary Repair**. *Ann Thorac Surg* 2006, **82**(6):2200-2206.

149. Kalfa D, Feier H, Loundou A, Fraisse A, Macé L, Metras D, Kreitmann B: **Cryopreserved homograft in the ross procedure: Outcomes and prognostic factors**. *J Heart Valve Dis* 2011, **20**(5):571-581.

150. Kalfa DM, Loundou A, de gorce YN, Fraisse A, Metras DR, Macé L, Kreitmann B: **Pulmonary position cryopreserved homograft in non-Ross patients: How to improve the results?** *Eur J Cardio-thorac Surg* 2012, **42**(6):981-987.

151. Kallio M, Pihkala J, Sairanen H, Mattila I: **Long-term results of the ross procedure in a population-based follow-up**. *Eur J Cardio-thorac Surg* 2015, **47**(5):e164-e170.

152. Kanter KR, Budde JM, Parks WJ, Tam VKH, Sharma S, Williams WH, Fyfe DA: **One hundred pulmonary valve replacements in children after relief of right ventricular outflow tract obstruction**. *Ann Thorac Surg* 2002, **73**(6):1801-1807.

153. Kanter KR, Fyfe DA, Mahle WT, Forbess JM, Kirshbom PM: **Results with the Freestyle Porcine Aortic Root for Right Ventricular Outflow Tract Reconstruction in Children**. *Ann Thorac Surg* 2003, **76**(6):1889-1895.

154. Karaskov A, Sharifulin R, Zheleznev S, Demin I, Lenko E, Bogachev-Prokophiev A: **Results of the ross procedure in adults: A single-centre experience of 741 operations**. *Eur J Cardio-thorac Surg* 2016, **49**(5):e97-e104.

155. Kim YJ, Choi YS, Lee JR, Rho JR: **Ventricular outflow tract reconstruction with polystan valved conduit**. *Asian Cardiovasc Thorac Ann* 2001, **9**(3):187-191.

156. Konertz W, Angeli E, Tarusinov G, Christ T, Kroll J, Dohmen PM, Krogmann O, Franzbach B, Napoleone CP, Gargiulo G: **Right ventricular outflow tract reconstruction with decellularized porcine xenografts in patients with congenital heart disease**. *J Heart Valve Dis* 2011, **20**(3):341-347.

157. Kuo JA, Hamby T, Munawar MN, Erez E, Tam VKH: **Midterm outcomes of right ventricular outflow tract reconstruction using the Freestyle xenograft**. *Congenit Heart Dis* 2019, **14**(4):651-656.

158. Lo Rito M, Davies B, Brawn WJ, Jones TJ, Khan N, Stickley J, Barron DJ: **Comparison of the Ross/Ross-Konno aortic root in children before and after the age of 18 months**. *Eur J Cardio-thorac Surg* 2014, **46**(3):450-457.

159. McBrien A, Chaudhari M, Crossland DS, Aspey H, Heads-Baister A, Griselli M, O'Sullivan J, Hasan A: **Single-centre experience of 101 paediatric and adult Ross procedures: Mid-term results**. *Interact Cardiovasc Thorac Surg* 2012, **14**(5):570-574.

160. Mokhles MM, Charitos E, Stierle U, Rajeswaran J, Blackstone E, Bogers AJC, Takkenberg JM, Sievers HH: **The fate of pulmonary conduits after the Ross procedure: Longitudinal analysis of the German-Dutch Ross registry experience**. *Heart* 2013, **99**(24):1857-1866.

161. Mookhoek A, Charitos EI, Hazekamp MG, Bogers AJJC, Hörer J, Lange R, Hetzer R, Sachweh JS, Riso A, Stierle U *et al*: **Ross Procedure in Neonates and Infants: A European Multicenter Experience**. *Ann Thorac Surg* 2015, **100**(6):2278-2284.

162. Nelson JS, Pasquali SK, Pratt CN, Yu S, Donohue JE, Loccoh E, Ohye RG, Bove EL, Hirsch-Romano JC: **Long-term survival and reintervention after the Ross procedure across the pediatric age spectrum**. *Ann Thorac Surg* 2015, **99**(6):2086-2095.

163. Palma G, Mannacio VA, Mastrogiovanni G, Russolillo V, Cioffi S, Mucerino M, Vosa C: **Bovine valved venous xenograft in pulmonary position: Medium term evaluation of risk factors for dysfunction and failure after 156 implants**. *J Cardiovasc Surg* 2011, **52**(2):285-291.

164. Pasquali SK, Shera D, Wernovsky G, Cohen MS, Tabbutt S, Nicolson S, Spray TL, Marino BS: **Midterm outcomes and predictors of reintervention after the Ross procedure in infants, children, and young adults**. *J Thorac Cardiovasc Surg* 2007, **133**(4):893-899.

165. Perri G, Polito A, Gandolfo F, Albanese SB, Carotti A: **Outcome of Standard and Bicuspidalized Cryopreserved Homografts for Primary Right Ventricular Outflow Tract Reconstruction**. *J Heart Valve Dis* 2015, **24**(1):83-88.

166. Ruffer A, Wittmann J, Potapov S, Purbojo A, Glockler M, Koch AM, Dittrich S, Cesnjevar RA: **Mid-term experience with the Hancock porcine-valved Dacron conduit for right ventricular outflow tract reconstruction**. *Eur J Cardiothorac Surg* 2012, **42**(6):988-995.

167. Ruzmetov M, Geiss DM, Shah JJ, Fortuna RS, Welke KF: **Does the Homograft for RVOT Reconstruction in Ross: Patients Fare Better than for Non-Ross Patients? A Single-Center Experience**. *J Heart Valve Dis* 2015, **24**(4):478-483.

168. Sandica E, Boethig D, Blanz U, Goerg R, Haas NA, Laser KT, Kececioglu D, Bertram H, Sarikouch S, Westhoff-Bleck M *et al*: **Bovine Jugular Veins versus Homografts in the Pulmonary Position: An Analysis across Two Centers and 711 Patients-Conventional Comparisons and Time Status Graphs as a New Approach**. *Thorac Cardiovasc Surg* 2016, **64**(1):25-35.

169. Schmid FX, Keyser A, Wiesenack C, Holmer S, Birnbaum DE: **Stentless xenografts and homografts for right ventricular outflow tract reconstruction during the Ross operation**. *Ann Thorac Surg* 2002, **74**(3):684-688.

170. Takkenberg JJM, Dossche KME, Hazekamp MG, Nijveld A, Jansen EWL, Waterbolk TW, Bogers AJJC: **Report of the Dutch experience with the Ross procedure in 343 patients**. *Eur J Cardio-thorac Surg* 2002, **22**(1):70-77.

171. Yang JH, Jun TG, Sung K, Kim WS, Lee YT, Park PW: **Midterm Results of Size-Reduced Cryopreserved Homografts for Right Ventricular Outflow Tract Reconstruction**. *Ann Thorac Surg* 2010, **89**(6):1821-1826.

172. Yuen CS, Lee KFL, Bhatia I, Yam N, Rocha BA, Yung TC, Chow PC, Au WKT: **Porcine Versus Pericardial Pulmonary Valve Replacement in Adults With Prior Congenital Cardiac Surgery: Midterm Outcomes**. *World J Pediatr Congenit Heart Surg* 2019, **10**(2):197-205.

173. Zhang HF, Chen G, Ye M, Yan XG, Tao QL, Jia B: **Mid- to long-term outcomes of bovine jugular vein conduit implantation in Chinese children**. *J Thorac Dis* 2017, **9**(5):1234-1239.

174. Zimmermann C, Attenhofer Jost C, Prêtre R, Mueller C, Greutmann M, Seifert B, Valsangiacomo Büchel E, Kretschmar O, Dave HH, Weber R: **Mid-term Outcome of 100 Consecutive Ross Procedures: Excellent Survival, But Yet to Be a Cure**. *Pediatr Cardiol* 2018, **39**(3):595-603.

175. Zubairi R, Malik S, Jaquiss RDB, Imamura M, Gossett J, Morrow WR: **Risk factors for prosthesis failure in pulmonary valve replacement**. *Ann Thorac Surg* 2011, **91**(2):561-565.

176. Cho JM, Puga FJ, Danielson GK, Dearani JA, Mair DD, Hagler DJ, Julsrud PR, Ilstrup DM: **Early and long-term results of the surgical treatment of tetralogy of Fallot with pulmonary atresia, with or without major aortopulmonary collateral arteries**. *J Thorac Cardiovasc Surg* 2002, **124**(1):70-81.

177. Christ T, Paun AC, Grubitzsch H, Holinski S, Falk V, Dushe S: **Long-term results after the ross procedure with the decellularized autotissue matrix P® bioprosthesis used for pulmonary valve replacement**. *Eur J Cardio-thorac Surg* 2019, **55**(5):885-892.

178. Erez E, Tam VKH, Doublin NA, Stakes J: **Repeat right ventricular outflow tract reconstruction using the medtronic freestyle porcine aortic root**. *J Heart Valve Dis* 2006, **15**(1):92-96.

179. Lee C, Kim YM, Lee CH, Kwak JG, Park CS, Song JY, Shim WS, Choi EY, Lee SY, Baek JS: **Outcomes of pulmonary valve replacement in 170 patients with chronic pulmonary regurgitation after relief of right ventricular outflow tract obstruction: Implications for optimal timing of pulmonary valve replacement**. *J Am Coll Cardiol* 2012, **60**(11):1005-1014.

180. Pratap H, Agarwal S, Moharana M, Singh S, Satsangi DK: **Right ventricular outflow tract reconstruction using Contegra® conduit in Tetralogy of Fallot: single centre experience**. 2010:1-4.

181. Allen BS, El-Zein C, Cuneo B, Cava JP, Barth MJ, Ilbawi MN: **Pericardial tissue valves and Gore-Tex conduits as an alternative for right ventricular outflow tract replacement in children**. *Ann Thorac Surg* 2002, **74**(3):771-777.

182. Kumar AS, Talwar S, Mohapatra R, Saxena A, Singh R: **Aortic valve replacement with the pulmonary autograft: Mid-term results**. *Ann Thorac Surg* 2005, **80**(2):488-494.

183. Adamson GT, McElhinney DB, Lui G, Meadows AK, Rigdon J, Hanley FL, Maskatia SA: **Secondary repair of incompetent pulmonary valves after previous surgery or intervention: Patient selection and outcomes**. *J Thorac Cardiovasc Surg* 2019.

184. Albanesi F, Sekarski N, Lambrou D, Von Segesser LK, Berdajs DA: **Incidence and risk factors for contegra graft infection following right ventricular outflow tract reconstruction: Long-term results**. *Eur J Cardio-thorac Surg* 2014, **45**(6):1070-1074.

185. Baskett RJ, Nanton MA, Warren AE, Ross DB: **Human leukocyte antigen-DR and ABO mismatch are associated with accelerated homograft valve failure in children: implications for therapeutic interventions**. *J Thorac Cardiovasc Surg* 2003, **126**(1):232-239.

186. Buchholz C, Mayr A, Purbojo A, Glöckler M, Toka O, Cesnjevar RA, Röffer A: **Performance of stented biological valves for right ventricular outflow tract reconstruction**. *Interact Cardiovasc Thorac Surg* 2016, **23**(6):933-939.

187. Danton MHD, Barron DJ, Stumper O, Wright JG, De Giovannni J, Silove ED, Brawn WJ: **Repair of truncus arteriosus: A considered approach to right ventricular outflow tract reconstruction**. *Eur J Cardio-thorac Surg* 2001, **20**(1):95-104.

188. Fan C, Yang Y, Xiong L, Yin N, Wu Q, Tang M, Yang J: **Reconstruction of the pulmonary posterior wall using in situ autologous tissue for the treatment of pulmonary atresia with ventricular septal defect**. *J Cardiothorac Surg* 2017, **12**(1):12.

189. Hartz RS, Deleon SY, Lane J, Dorotan J, Joyce J, Urbina E, Ross-Ascuitto N, Ascuitto R: **Medtronic Freestyle Valves in Right Ventricular Outflow Tract Reconstruction**. *Ann Thorac Surg* 2003, **76**(6):1896-1900.

190. Horer J, Schreiber C, Dworak E, Cleuziou J, Prodan Z, Vogt M, Holper K, Lange R: **Long-term results after the Rastelli repair for transposition of the great arteries**. *Ann Thorac Surg* 2007, **83**(6):2169-2175.

191. Javadpour H, Veerasingam D, Wood AE: **Calcification of homograft valves in the pulmonary circulation - Is it device or donation related?** *Eur J Cardio-thorac Surg* 2002, **22**(1):78-81.

192. Raju V, Myers PO, Quinonez LG, Emani SM, Mayer JE, Pigula FA, Del Nido PJ, Baird CW: **Aortic root translocation (Nikaidoh procedure): Intermediate follow-up and impact of conduit type**. *J Thorac Cardiovasc Surg* 2015, **149**(5):1349-1355.

193. Sievers HH, Stierle U, Charitos EI, Takkenberg JJM, Hörer J, Lange R, Franke U, Albert M, Gorski A, Leyh RG *et al*: **A multicentre evaluation of the autograft procedure for young patients undergoing aortic valve replacement: Update on the German Ross Registry**. *Eur J Cardio-thorac Surg* 2016, **49**(1):212-218.

194. Talwar S, Malankar D, Garg S, Choudhary SK, Saxena A, Velayoudham D, Kumar AS: **Aortic valve replacement with biological substitutes in children**. *Asian Cardiovasc Thorac Ann* 2012, **20**(5):518-524.

195. Vitanova K, Cleuziou J, Hörer J, Kasnar-Samprec J, Vogt M, Schreiber C, Lange R: **Which type of conduit to choose for right ventricular outflow tract reconstruction in patients below 1 year of age?** *Eur J Cardio-thorac Surg* 2014, **46**(6):961-966.

196. Weimar T, Charitos EI, Liebrich M, Roser D, Tzanavaros I, Doll N, Hemmer WB: **Quo vadis pulmonary autograft - The ross procedure in its second decade: A single-center experience in 645 patients**. *Ann Thorac Surg* 2014, **97**(1):167-174.

197. Dang Van S, Pavy C, Guimbretière G, Boulanger J, Maminirina P, Guerin P, Baron O: **Results of large pulmonary homograft implantation for right ventricular outflow tract reconstruction**. *J Card Surg* 2021, **36**(2):442-448.

198. Schlein J, Ebner BE, Geiger R, Simon P, Wollenek G, Moritz A, Gamillscheg A, Base E, Laufer G, Zimpfer D: **Long-term outcomes after the paediatric Ross and Ross-Konno procedures**. *Interact Cardiovasc Thorac Surg* 2021.

199. Ghiselli S, Carro C, Uricchio N, Annoni G, Marianeschi SM: **Mid-to long-term follow-up of pulmonary valve replacement with BioIntegral injectable valve**. *Eur J Cardio-thorac Surg* 2021, **59**(2):325-332.

200. Romeo JLR, Papageorgiou G, Da Costa FFD, Sievers HH, Bogers AJJC, El-Hamamsy I, Skillington PD, Wynne R, Mastrobuoni S, El Khoury G *et al*: **Long-term Clinical and Echocardiographic Outcomes in Young and Middle-aged Adults Undergoing the Ross Procedure**. *JAMA Cardiol* 2021, **6**(5):539-548.

201. Lenoir M, Chenu C, Amrous A, Casalta AC, Guidon C, Aldebert P, Macé L: **Right ventricular remodelling after endo-exclusion during pulmonary valve replacement: evaluation by cardiac magnetic resonance**. *Eur J Cardiothorac Surg* 2021.

202. Derridj N, Villemain O, Khoshnood B, Belhadjer Z, Gaudin R, Raisky O, Bonnet D: **Outcomes after common arterial trunk repair: Impact of the surgical technique**. *J Thorac Cardiovasc Surg* 2020.

203. Hoashi T, Ichikawa H, Hirose K, Horio N, Sakurai T, Matsuhisa H, Ohsima Y, Sakurai H, Kasahara S, Sakamoto K: **Mid-term outcomes of Contegra implantation for the reconstruction of the right ventricular outflow tract to proximal branch pulmonary arteries: Japan multicentre study**. *Interact Cardiovasc Thorac Surg* 2021.

204. Bové T, Bradt N, Martens T, De Wolf D, François K, de Beco G, Sluysmans T, Rubay J, Poncelet A: **The Pulmonary Autograft After the Ross Operation: Results of 25-Year Follow-Up in a Pediatric Cohort**. *Ann Thorac Surg* 2021, **111**(1):159-167.

205. Chauvette V, Bouhout I, Lefebvre L, Tarabzoni M, Chamberland ME, Poirier N, Demers P, Chu MWA, Perron J, El-Hamamsy I: **The Ross procedure is a safe and durable option in adults with infective endocarditis: a multicentre study**. *Eur J Cardiothorac Surg* 2020, **58**(3):537-543.

206. Fernandez-Carbonell A, Rodriguez-Guerrero E, Merino-Cejas C, Conejero-Jurado MT, Villalba-Montoro R, Romero-Morales MDC, Alados-Arboledas P, Casares-Mediavilla J, Fernandez-Carbonell M, Lopez-Cillero P *et al*: **Predictive Factors for Pulmonary Homograft Dysfunction After Ross Surgery: A 20-Year Follow-up**. *Ann Thorac Surg* 2021, **111**(4):1338-1344.

207. Murin P, Weixler VHM, Moulla-Zeghouane J, Romanchenko O, Schleiger A, Lorenzen V, Sinzobahamvya N, Zacek P, Photiadis J, Cho MY: **Subcoronary Ross/Ross-Konno operation in children and young adults: initial single-centre experience**. *Eur J Cardiothorac Surg* 2021, **59**(1):226-233.

208. Georgiev S, Ewert P, Eicken A, Hager A, Hörer J, Cleuziou J, Meierhofer C, Tanase D: **Munich Comparative Study: Prospective Long-Term Outcome of the Transcatheter Melody Valve Versus Surgical Pulmonary Bioprosthesis with up to 12 Years of Follow-Up**. *Circ Cardiovasc Interventions* 2020, **13**(7).

209. Arribas-Leal JM, Garcia-Vieites M, Jimenez-Aceituna A, Canovas-Lopez S, Gutierrez F, Bautista-Hernandez V: **Results of Pulmonary Valve Replacement with a Newly Introduced Bioprosthesis in Children and Young Adults with Congenital Heart Disease**. *Struct Heart* 2021, **5**(1):75-78.

210. Callahan CP, Jegatheeswaran A, Blackstone EH, Karamlou T, Baird CW, Ramakrishnan K, Herrmann JL, Brown JW, Nelson JS, Polimenakos AC *et al*: **Time-related risk of pulmonary conduit re-replacement: a Congenital Heart Surgeons' Society Study**. *Ann Thorac Surg* 2021.

211. Maeda K, Lui GK, Zhang Y, Maskatia SA, Romfh A, Yarlagadda VV, Hanley FL, McElhinney DB: **Durability of Pulmonary Valve Replacement with Large Diameter Stented Porcine Bioprostheses**. *Semin Thorac Cardiovasc Surg* 2021.

212. Naimo PS, Bell D, Fricke TA, d'Udekem Y, Brizard CP, Alphonso N, Konstantinov IE: **Truncus arteriosus repair: A 40-year multicenter perspective**. *J Thorac Cardiovasc Surg* 2021, **161**(1):230-240.

213. Raja J, Menon S, Sameer M, Ramanan S, Baruah SD, Gopalakrishnan A, Dharan BS: **Midterm results of homografts in pulmonary position: a retrospective single-center study**. *Indian J Thorac Cardiovasc Surg* 2021, **37**(2):129-137.

214. Selcuk A, Kilic Y, Korun O, Yurdakok O, Cicek M, Altin HF, Altuntas Y, Yilmaz EH, Sasmazel A, Aydemir NA: **High incidence of fever in patients after biointegral pulmonic valved conduit implantation**. 2021.

215. Dorobantu DM, Sharabiani MTA, Taliotis D, Parry AJ, Tulloh RMR, Bentham JR, Caputo M, Van Doorn C, Stoica SC: **Age over 35 years is associated with increased mortality after pulmonary valve replacement in repaired tetralogy of Fallot: Results from the UK National Congenital Heart Disease Audit database**. *Eur J Cardio-thorac Surg* 2020, **58**(4):825-831.

216. Kim DH, Choi ES, Kwon BS, Yun TJ, Cha SG, Baek JS, Yu JJ, Kim YH, Park CS: **Pulmonary valve replacement following repair of tetralogy of Fallot: comparison of outcomes between bio- and mechanical prostheses**. *Eur J Cardiothorac Surg* 2021.

217. Tominaga Y, Taira M, Kido T, Kanaya T, Araki K, Watanabe T, Sakaniwa R, Toda K, Kuratani T, Ueno T *et al*: **Persistent end-diastolic forward flow after pulmonary valve replacement in patients with repaired tetralogy of Fallot**. *Eur J Cardiothorac Surg* 2021.

218. Yasukawa T, Hoashi T, Imai K, Okuda N, Fukuda T, Ohuchi H, Kurosaki K, Ichikawa H: **The reduced left ventricular stroke volume does not fully recover after pulmonary valve replacement in patients with repaired tetralogy of Fallot**. *Eur J Cardiothorac Surg* 2021.
